# Synthesis of rhamnosylated arginine glycopeptides and determination of the glycosidic linkage in bacterial elongation factor P†
†Electronic supplementary information (ESI) available: Materials and methods, compound characterization, 1D and 2D NMR and mass spectrometry data of glycopeptides. See DOI: 10.1039/c6sc03847f



**DOI:** 10.1039/c6sc03847f

**Published:** 2016-12-12

**Authors:** Siyao Wang, Leo Corcilius, Phillip P. Sharp, Andrei Rajkovic, Michael Ibba, Benjamin L. Parker, Richard J. Payne

**Affiliations:** a School of Chemistry , The University of Sydney , Sydney , NSW 2006 , Australia . Email: richard.payne@sydney.edu.au; b ACRF Chemical Biology Division , Walter and Eliza Hall Institute of Medical Research , 1G Royal Parade , VIC3052 , Australia; c Department of Microbiology and Center for RNA Biology , Ohio State University , Columbus , Ohio , USA; d Charles Perkins Centre , The University of Sydney , NSW 2006 , Australia

## Abstract

A new class of N-linked protein glycosylation – arginine rhamnosylation – has recently been discovered as a critical modification for the function of bacterial elongation factor P (EF-P). Herein, we describe the synthesis of suitably protected α- and β-rhamnosylated arginine amino acid “cassettes” that can be directly installed into rhamnosylated peptides. Preparation of a proteolytic fragment of *Pseudomonas aeruginosa* EF-P bearing both α- and β-rhamnosylated arginine enabled the unequivocal determination of the native glycosidic linkage to be α through 2D NMR and nano-UHPLC-tandem mass spectrometry studies.

## Abstract 2

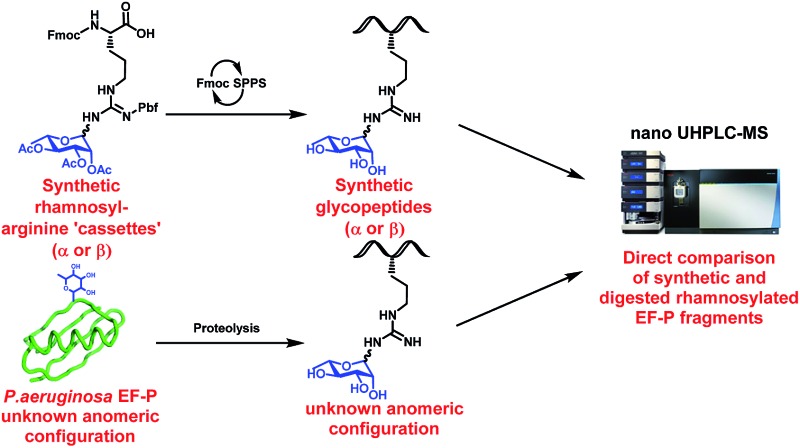
We describe the synthesis and incorporation of α- and β-configured rhamnosyl arginine cassettes into *Pseudomonas aeruginosa* elongation factor P-derived glycopeptides. These were used to unequivocally determine the native anomeric configuration of the rhamnose moiety in EF-P.

Protein glycosylation is the most ubiquitous post-translational modification in nature, estimated to occur on more than 50% of human proteins.^1^ In eukaryotes, glycosylation is a key modulator of biological recognition events, including cell adhesion, differentiation and growth.^2^ In addition, the presence of glycans can affect the structure and activity of a given protein and aberrant glycan structures have been implicated in a number of serious illnesses including cancer^3^ and autoimmune diseases.^4^ Eukaryotic protein glycosylation can be divided into two main classes: *O*-glycosides, whereby a glycan is covalently linked to the hydroxyl moiety of serine, threonine, tyrosine, hydroxylysine or hydroxyproline with α- or β-anomeric configuration,^5,6^ or *N*-glycosides, in which *N*-acetylglucosamine is β-linked to the amide side chain of an asparagine residue (within an Asn-Xaa-Ser/Thr consensus sequence).^6,7^ Importantly, the number of eukaryotic proteins known to be glycosylated has dramatically increased in recent years owing, in major part, to improvements in glycoproteomic mass spectrometry methodologies.^8–10^

While glycosylation was initially deemed to be a modification exclusive to higher eukaryotic organisms, it is now established that widespread O- and N-linked glycosylation of bacterial proteins also occurs with similar linkage types to the eukaryotic counterparts.^11,12^ However, since the field of bacterial protein glycosylation is still in its infancy, the effect of glycosylation on structure and function is still not well understood. Very recently, a number of new glycosylation motifs have been discovered in bacteria, including *S*-glycosylation^13–15^ of cysteine and *N*-glycosylation of the guanidine side chain of arginine (Arg) residues.^16–19^ This latter modification was first observed as a β-*N*-acetyl-d-glucosamine (β-GlcNAc) modification on specific Arg residues within human proteins bearing the death domain motif, including TRADD, FADD, TNFR1 and the kinase RIPK1.^16,17^ Intriguingly, this modification was shown to be performed by a type III secretion system effector protein called NleB produced by enteropathogenic strains of *Escherichia coli*. Functionally, β-GlcNAcylation of specific Arg residues within death domains by NleB prevents cell death pathways by blocking downstream signalling that would normally lead to apoptosis or necroptosis of *E. coli*-infected cells.^16,17^

In more recent work, Lassak and Jung and co-workers and Rajkovic *et al.* discovered l-rhamnosylation of Arg residues within bacterial translation elongation factor P (EF-P), a protein required for preventing ribosome stalling when translating polyproline motifs.^18,19^ To date, the modification has been discovered on EF-P of the γ-proteobacterium *Shewanella oneidensis*,^18^ the pathogenic bacteria *Pseudomonas aeruginosa* ^19^ and *Neisseria meningitidis*.^20^ In all three bacteria rhamnosylation of Arg-32 was shown to be critical for the function of EF-P in assisting translation and for the pathogenicity of *P. aeruginosa in vitro*.^18^ The glycosylation was also shown to be performed by EarP, a rhamnosyltransferase that employs dTDP-l-rhamnose as the substrate. An important piece of information that was not revealed in these studies was the stereochemistry at the anomeric center of l-rhamnose appended to the side chain of Arg-32. Establishing the stereochemical configuration of this centre is critical to determine the conformational effect of the carbohydrate on the underlying peptide backbone and to ascertain whether EarP is an inverting or retaining glycosyltransferase. In this work we were interested in developing an efficient synthetic route toward suitably protected rhamnosylated arginine amino acids with both α- and β-configuration. In doing so we would be able to synthesize glycopeptides bearing both rhamnosyl configurations for unequivocal determination of the anomeric configuration of the rhamnose moiety on EF-P. While this work was in progress, Li *et al.* reported an NMR study on recombinant *S. oneidensis* EF-P that on the basis of a *J*_C1,H1_ coupling constant in an undecoupled HSQC experiment (*J* = 167 Hz) and the absence of a H1–H5 NOE (Nuclear Overhauser Effect) would suggest that the rhamnose is α-configured at the anomeric centre.^21^ Herein, we report a comprehensive analysis of the stereochemistry of the rhamnosylation of *P. aeruginosa* EF-P through 2D NMR spectroscopy, HPLC, nanoLC and tandem mass spectrometric comparison of synthetic rhamnosylated peptides with the proteolytic fragment of isolated *P. aeruginosa* EF-P and recombinant *P. aeruginosa* EF-P both bearing the modification. Our studies provide strong evidence that independently supports the notion that rhamnose is α-linked to arginine in bacterial EF-P proteins. We also reveal that the α-anomer of rhamnosylated arginine is configurationally labile and undergoes base-promoted endocyclic ring opening anomerization to the β-anomer which, to our knowledge has not been previously observed in native N-linked glycosides.

We began by targeting proteolytic fragments of *P. aeruginosa* EF-P bearing both α- and β-configured rhamnosylated Arg. Hu and co-workers have reported the synthesis of peptides containing *N*-GlcNAcylated^22^ and *N*-rhamnosylated^21^ Arg through a solid-phase guanidinylation strategy whereby the requisite glycosyl isothiourea was reacted with a resin-bound ornithine-containing peptide. While the direct glycosylation of resin-bound peptides is attractive from a synthetic standpoint, we were concerned about the configurational stability of the rhamnose linkage under the acidic and basic conditions employed for cleavage and deprotection of the glycopeptide following solid-phase peptide synthesis (SPPS). Therefore we opted for a building block strategy, in which pre-fabricated α- and β-rhamnosylated arginine building blocks **1** and **2** could be robustly interrogated by 2D NMR techniques and the stability of the individual glycosylamino acids examined under acidic and basic conditions before direct incorporation into glycopeptides as ‘cassettes’ through Fmoc-strategy SPPS (Scheme 1).

**Scheme 1:**
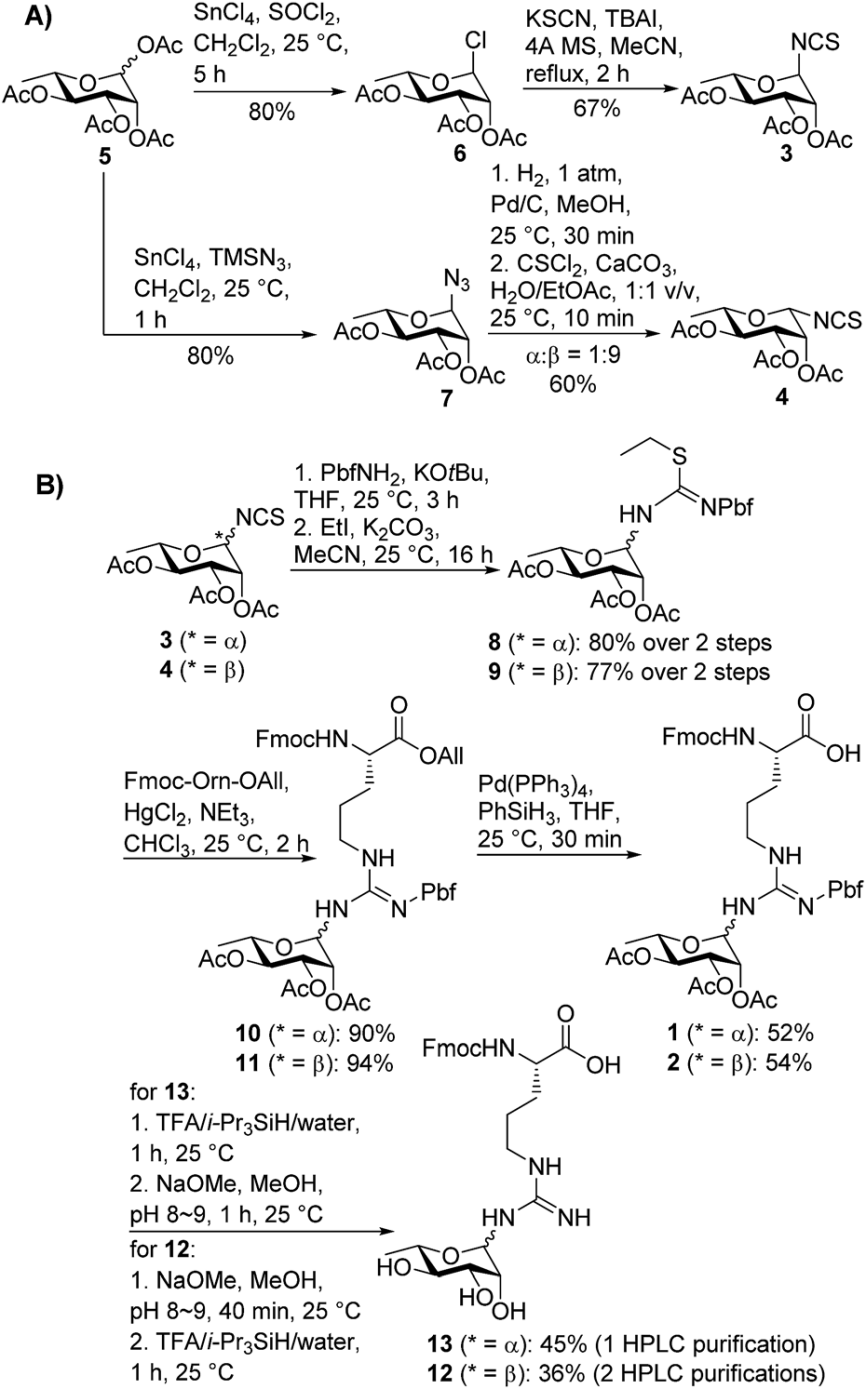
(A) Stereoselective synthesis of α- and β-configured rhamnosyl isothiocyanates **3** and **4**; (B) synthesis of suitably protected rhamnosylated arginine building blocks **1** and **2** with α- and β-anomeric configuration, respectively.

In order to mask the nucleophilicity of the guanidinium side chain of Arg during SPPS, glycosylamino acid building blocks **1** and **2** were designed with an acid-labile 2,2,4,6,7-pentamethyldihydrobenzofuran-5-sulfonyl (Pbf) side chain protecting group, commonly employed for the protection of the parent amino acid in Fmoc-SPPS.^23^ Synthesis of the two rhamnosylated arginine building blocks **1** and **2** began with preparation of the α- and β-configured glycosyl isothiocyanates **3** ^21^ and **4**, both of which were accessed from rhamnosyl acetate **5** ^24^ as a common precursor (Scheme 1A). To access α-isothiocyanate **3**, rhamnosyl chloride **6** ^25^ was treated with KSCN in the presence of catalytic TBAI in refluxing acetonitrile. Under these conditions complete 1,2-trans (α) stereoselectivity was afforded due to anchimeric assistance from the neighbouring acyloxy substituent.^26^ Alternatively, treatment of the glycosyl acetate **5** with SnCl_4_ and TMSN_3_ provided glycosyl azide **7**.^27^ This azide was reduced to the glycosylamine using H_2_,Pd/C and, without purification, was treated with thiophosgene and CaCO_3_ to afford the glycosyl isothiocyanate **4** as predominantly the β-isomer (α : β 1 : 9).^28^ In this case β-selectivity was presumed to result from base-catalysed isomerization of the α-glycosylamine to the thermodynamically favoured equatorial (β) isomer.^27^ Importantly, 2D NOESY NMR experiments of the two glycosyl isothiocyanates **3** and **4** confirmed the respective anomeric configurations (see ESI†).

With the two rhamnosylated isothiocyanates **3** and **4** in hand, we next sought to introduce the Pbf-protected guanidine moiety (Scheme 1B). Direct treatment of isothiocyanates **3** and **4** with deprotonated Pbf-NH_2_ proved to be the optimal means to generate-Pbf protected thioureas from **3** and **4** (Scheme 1B).^29^ These were not isolated, but rather treated directly with EtI and K_2_CO_3_ in MeCN to afford ethyl isothioureas **8** and **9** in good yields. Subsequent Hg(ii)-promoted guanidinylation of each of the isothioureas with Fmoc-Orn-OAll then gave the corresponding glycosylamino acid derivatives **10** and **11** in excellent yields. Finally, deprotection of the allyl ester moiety with tetrakis(triphenylphosphine)palladium(0) and phenylsilane afforded the suitably protected rhamnosylated arginine building blocks **1** and **2** ready for direct incorporation into glycopeptides by Fmoc-SPPS.

At this stage, we set out to fully characterize building blocks **1** and **2** *via* NMR spectroscopy. Unfortunately, despite screening a number of solvents and temperatures in NMR experiments, proton signals from the pyranosyl ring were extremely broad, thus making it difficult to assign the configuration of the anomeric centre through 2D NMR experiments. This presumed rotameric (or tautomeric) effect was alleviated by deprotection of both the acetyl and Pbf moieties on building blocks **1** and **2** (*via* acidolytic deprotection of the Pbf followed by deacetylation by treatment with sodium methoxide in MeOH). These conditions were selected to mirror the intended deprotection sequence of the rhamnosylated peptide following assembly by Fmoc-SPPS. Deprotection of building block **2** (with β-anomeric configuration) proceeded smoothly to afford triol **12** in 36% yield following HPLC purification. The anomeric configuration of **12** was unequivocally determined through 1D and 2D NMR analysis (see ESI†). Unexpectedly, significant anomerization was observed when the same deprotection sequence was applied to glycosylamino acid **1** bearing α-configuration at the anomeric centre. Specifically, upon treatment of **1** with TFA followed by methoxide, a 4 : 5 α : β mixture of anomers was generated (see ESI†). Upon further examination, it was determined that the anomerization resulted from the deacetylation step, suggesting a base-catalyzed mutarotation event. Thankfully, reversal of the deprotection sequence (*i.e.* treatment with NaOMe in MeOH prior to treatment with TFA) led to almost complete retention of the anomeric stereochemistry (α : β 9 : 1), suggesting that the presence of the electron-withdrawing Pbf protecting group was necessary to suppress base-catalysed anomerization. The configuration of the anomeric linkage was able to be confirmed by 2D NOESY analysis of the deprotected glycosylamino acids **12** and **13** (see ESI† for details). Importantly, β-configured rhamnosylated Arg **12** exhibited a distinct NOE between H1 and H5 as well as between H1 and H3, whereas these NOEs were absent in the corresponding α-anomer **13** (see ESI†). HSQC experiments without ^1^H decoupling were also performed to determine the ^1^H–^13^C coupling constant at C-1 (*J*_C1,H1_ = 167 Hz for **13** and 154 Hz for **12**, see ESI† for details). Deprotected glycosylamino acids **12** and **13** were also found to be stable in 0.1% TFA in MeCN/H_2_O, which is a common eluent system used to purify peptides and glycopeptides. The above finding that the deprotected rhamnosyl arginine isomerizes under basic conditions highlights a unique behavior of the Arg-N-linked glycan motif as the more common Asn-N-linked and O-linked glycans are not susceptible to anomerization under these conditions. Mechanistically, we propose that anomerization of α-rhamnosylated arginine proceeds through endocyclic ring opening that is facilitated by a lone pair on one of the guanidine ω-nitrogen atoms (see Scheme 2). This mechanistic proposal is supported by previous observations that mannosyl thiourea derivatives are susceptible to base-catalysed endocyclic ring opening anomerization.^30^

**Scheme 2:**
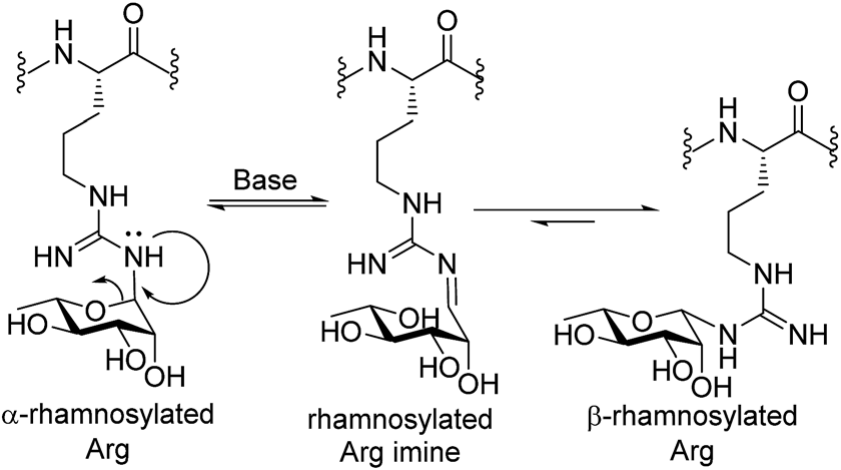
Proposed base-catalyzed anomerization of α-rhamosylated Arg to β-rhamnosylated Arg.

With the two target rhamnosyl arginine building blocks **1** and **2** in hand, we began assembly of target peptide fragments **14** and **15** bearing α- and β-configured rhamnosyl arginine, respectively *via* Fmoc-SPPS (Scheme 3). These glycopeptides correspond to the Lys-C proteolysed fragment of *P. aeruginosa* EF-P that has been characterized *via* MS(MS^3^).^19^ Synthesis of these glycopeptides began by pre-loading of Chemmatrix® Trityl-OH resin with Fmoc-Lys(Boc)-OH, followed by standard Fmoc-SPPS protocols to generate resin-bound hexapeptide **16**. Building blocks **1** or **2** (2.2 equiv.) were then coupled onto the resin-bound peptide using 1-[bis(dimethylamino)methylene]-1*H*-1,2,3-triazolo[4,5-*b*]pyridinium 3-oxide hexafluorophosphate (HATU, 2.0 equiv.), 1-hydroxy-7-azabenzotriazole (HOAt, 3.0 equiv.)^31^ and sym-collidine (2.4 equiv.) as the base. These conditions facilitated complete incorporation of the glycosylamino acids (as judged by HPLC-MS analysis) to afford resin-bound glycopeptide **17** and **18**, which were further elongated to the desired resin bound nonapeptides **19** and **20** following standard Fmoc-SPPS protocols. Deprotection of the β-rhamnosylated peptide **20** was carried out through acidolytic side chain deprotection and cleavage from the resin followed by Zemplén deacetylation at pH 8–9 to afford peptide **15** in 30% isolated yield (based on the original resin loading). In contrast, the configurationally unstable α-rhamnosylated peptide **19** was cleaved from the resin using a weakly acidic solution of hexafluoroisopropanol (HFIP) in DCM (30% v/v) to afford the fully side-chain protected peptide, which was then carefully deacetylated under Zémplen conditions before global side chain deprotection with TFA:i-Pr_3_SiH/H_2_O to afford predominantly the α-glycopeptide **14** (9 : 1 α : β). This Zémplen deacetylation reaction required careful monitoring by UPLC-MS as either excessively high pH (>9) or extended reaction time still led to significant anomerization. Indeed, as with amino acid **13**, treatment of α-rhamnosylated EF-P glycopeptide **14** with dilute methoxide (pH 9.0) for 2 h led to significant anomerization to the corresponding β-configured glycopeptide **15** (1 : 2 ratio **14** : **15**, see Scheme 3 and ESI†). Unfortunately, an attempt to avoid anomerization completely through on-resin deacetylation of **19** with 10 vol% hydrazine monohydrate in DMF, followed by cleavage from the resin with an acidic cocktail, led to significant anomerization [1 : 1 **14**(α) : **15**(β), see ESI†]. Thus, careful off-resin treatment with sodium methoxide in MeOH was deemed optimal for deacetylation of the α-configured glycopeptide. The optimal deprotection sequence of resin-bound **19** therefore involved release of the side chain protected glycopeptide from the resin using HFIP, careful deacetylation with methoxide (pH 8–9) followed by global deprotection with an acidolytic cocktail comprising TFA/i-Pr_3_SiH/H_2_O to afford predominantly α-rhamnosylated peptide **14** (α : β 9 : 1) in 17% yield based on the original resin loading. It should be noted that during the above experiments we were also able to confirm the identity of the proposed imine intermediate that forms *via* the base-catalysed endocyclic ring opening anomerization (Scheme 2). Specifically, following co-treatment of **14** with methoxide and sodium borohydride, the ring opened imine could be trapped as the corresponding amine (see ESI†).

**Scheme 3:**
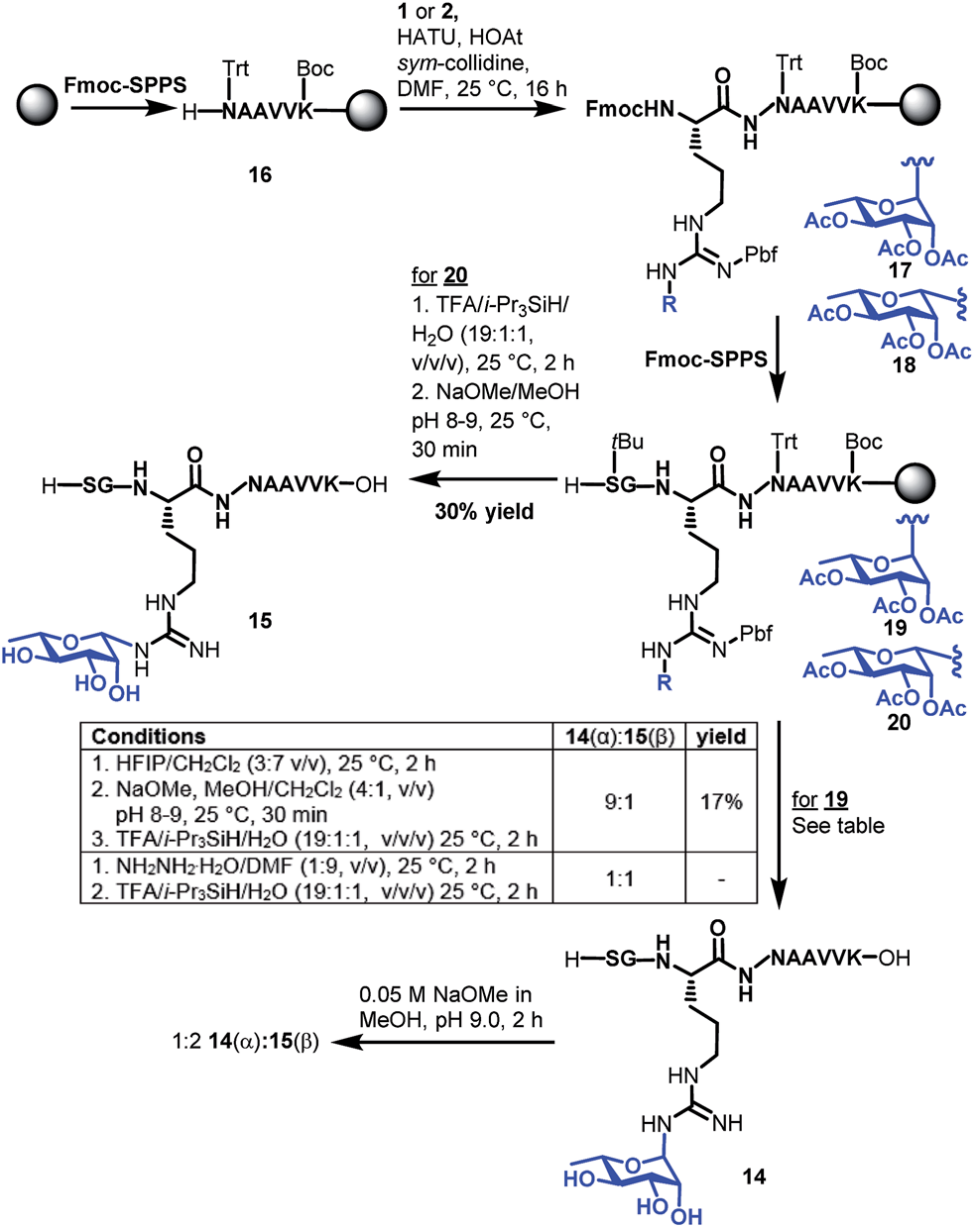
Synthesis of Lys-C proteolytic fragments of *P. aeruginosa* EF-P bearing α-rhamose (**14**) and β-rhamnose (**15**) anomeric configurations.

We next extensively characterized glycopeptides **14** and **15** with a view to performing comparative studies with native *P. aeruginosa* EF-P for determination of the anomeric configuration of the rhamnose moiety in the native protein. 2D NMR experiments were initially used to unequivocally confirm that the rhamnopyranosyl moiety of glycopeptides **14** and **15** was α- and β-configured, respectively. We first performed ^1^H coupled HSQC experiments that provided a ^1^H–^13^C coupling constant at the anomeric centre of 167 Hz for the α-linkage of **14** and 156 Hz for the β-linkage of **15**, very similar to the values obtained for the parent rhamnosylamino acids **1** and **2** (*vide supra*) (Fig. 1A). It should be noted that the coupling constant for **14** was also in agreement with that determined for recombinant *S. oneidensis* EF-P homologue (^1^ *J*_C–H_ = 167 Hz) reported by Li *et al.* ^21^ Selective ROESY irradiation at H1 was also performed which showed a NOE with H5 of **15** (with a diaxial H1–H5 relationship, see Fig. 1B) but not for **14** (see Fig. 1C). Finally, we developed analytical HPLC-MS conditions to separate the two anomeric glycopeptides **14** and **15** with a retention time difference of ∼1 min (see Fig. 1D for individual and overlayed HPLC traces).

**Fig. 1:**
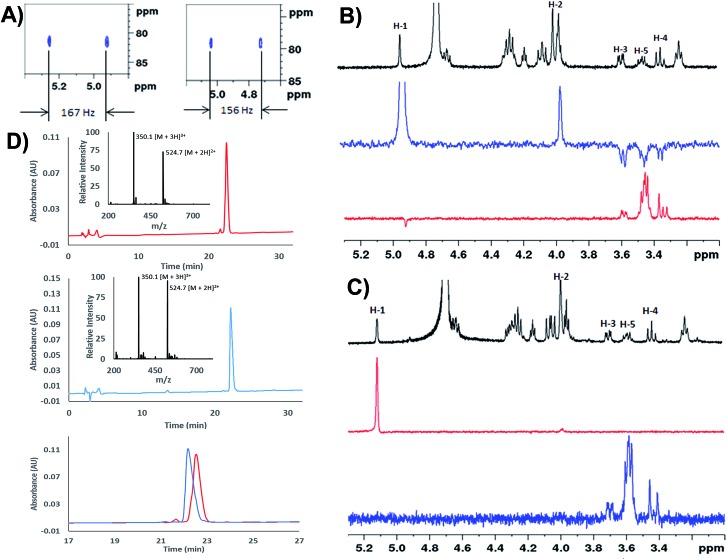
(A) Zoom in of the ^1^H–^13^C HSQC without proton decoupling for: H-SGR(α-Rha)NAAVVK-OH (**14**), ^1^ *J*_CH_ = 167 Hz (left) and H-SGR(β-Rha)NAAVVK-OH (**15**), ^1^ *J*_CH_ = 156 Hz (right); (B) selective ROESY experiments for H-SGR(β-Rha)NAAVVK-OH (**15**). Black = zoom in of ^1^H NMR spectrum, blue = selected ROESY by irradiating H-1; red: selected ROESY by irradiating H5. The through-space NOE relationship between diaxial H5 and H1 can be observed; (C) selective ROESY experiments for H-SGR(α-Rha)NAAVVK-OH (**14**). Black = zoom in of ^1^H NMR spectrum, blue = selected ROESY by irradiating H-5; red: selected ROESY by irradiating H1. No NOE was observed; (D) analytical HPLC traces of H-SGR(α-Rha)NAAVVK-OH **14** (top), H-SGR(β-Rha)NAAVVK-OH **15** (middle), overlay of HPLC chromatograms of **14** and **15** (bottom); gradient: 100% A for 2 min then 0–20% B over 30 min, Waters Atlantis® T3 C18 Column at 0.2 mL min^–1^.

Having prepared the two possible anomers of the Lys-C proteolysed glycopeptides of *P. aeruginosa* EF-P (**14** and **15**) and confirmed their anomeric stereochemistry, we next performed comparative analysis to the Lys-C glycopeptide produced from isolated *P. aeruginosa* EF-P prepared as reported previously^19^ *via* nano-ultra high pressure liquid chromatography (nanoUHPLC) coupled to tandem mass spectrometry (see ESI†). Separation of the two anomeric rhamnosylated glycopeptides **14** and **15** was achieved with nanoUHPLC using isocratic elution on a 50 cm column (Fig. 2A). Analysis of the Lys-C proteolysed glycopeptide fragment of purified *P. aeruginosa* EF-P clearly showed a retention time alignment with the α-rhamnosylated glycopeptide **14** providing convincing evidence that the rhamnose moiety appended to Arg-32 of *P. aeruginosa* EF-P is α-linked. We further confirmed this retention time-based evidence by spiking either the synthetic α-rhamnosylated (**14**) or β-rhamnosylated glycopeptide (**15**) into a sample of Lys-C proteolysed *P. aeruginosa* EF-P, followed by analysis by nanoUHPLC-MS/MS (see Fig. 81, ESI†). Addition of the α-rhamnosylated (**14**) glycopeptide increased the intensity of a single chromatographic peak while spiking in the β-rhamnosylated glycopeptide (**15**) produced two peaks of similar intensity, thus further supporting the α-anomeric configuration of rhamnose in *P. aeruginosa* EF-P. Interestingly, we also observed <5% of the β-rhamnosylated peptide in the proteolysed sample. This observation is consistent with the endocyclic ring opening anomerization observed during preparation of the synthetic glycopeptides. It is important to note that this anomerization occurred despite only neutral buffer conditions being used and suggests that rhamnosylated peptides and proteins (whether expressed, isolated or synthesised) must be handled carefully during isolation, purification and other manipulations to prevent extensive anomerization. In order to further strengthen our observations on the anomeric configuration of rhamnose in EF-P, we produced recombinant *P. aeruginosa* EF-P in *E. coli* and performed *in vitro* rhamnosylation with EarP using dTDP-l-rhamnose as a substrate (see ESI†).^18,19^ Analysis of the Lys-C proteolysed glycopeptide fragment also suggested α-configuration of the rhamnose moiety based on retention time analysis with synthetic **14** and **15** by nanoUHPLC. Finally, we performed fragmentation of the rhamnosylated glycopeptide from *P. aeruginosa* with ETD MS/MS which localised the glycosylation site to Arg32^19^ (see Fig. 82, ESI†).

**Fig. 2:**
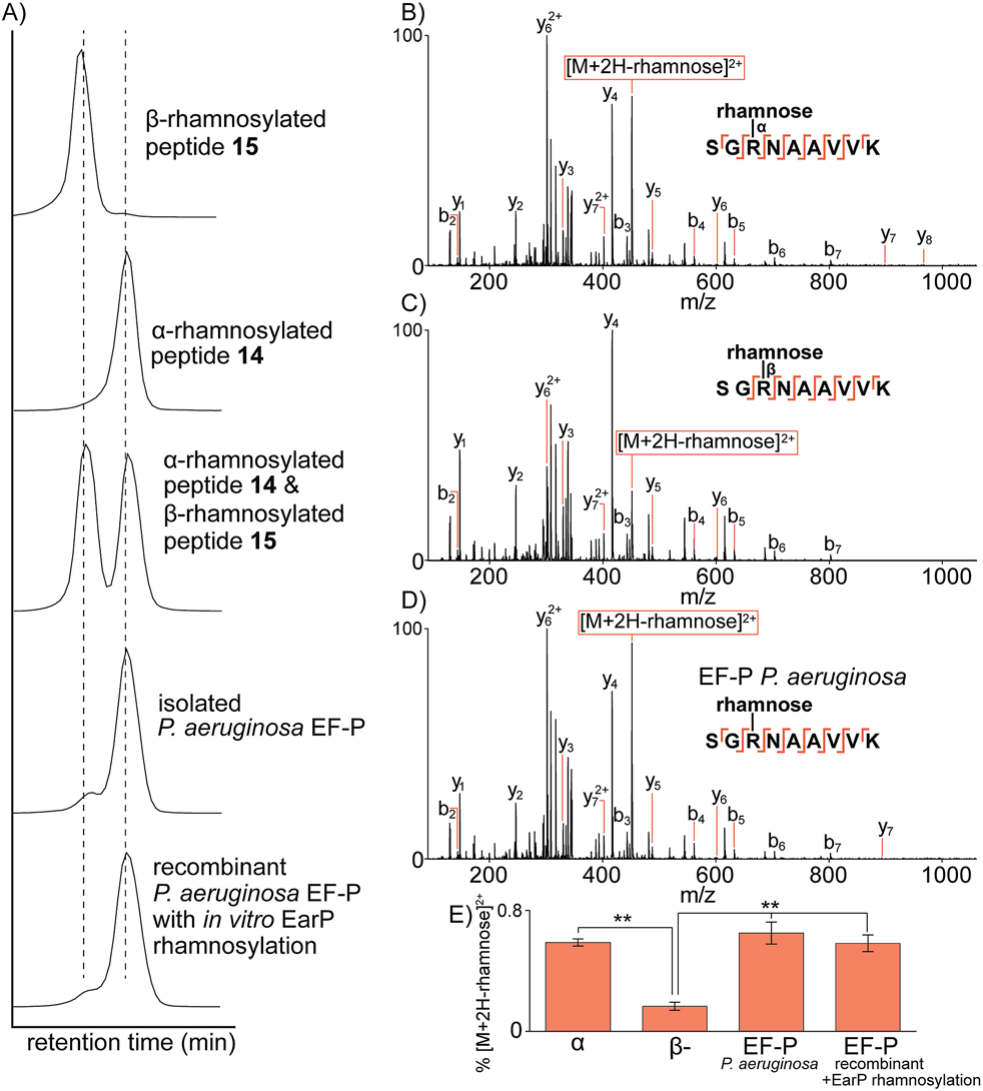
Characterization of rhamnosylated glycopeptides by nanoUHPLC-MS/MS. (A) Extracted-ion chromatographic elution profiles of synthetic glycopeptides **14** and **15**, an equimolar mixture of **14** and **15**, and Lys-C digested EF-P purified from *P. aeruginosa* or recombinant EF-P (from *E. coli*) with *in vitro* rhamnosylation by EarP. The extracted ion mass window was 349.86–349.87 *m*/*z* corresponding to the triply charged glycopeptide. (B) Ion-trap CID MS/MS analysis of the α-linked synthetic glycopeptide. (C) Ion-trap CID MS/MS analysis of the β-linked synthetic glycopeptide. (D) Ion-trap CID MS/MS analysis of the glycopeptide identified from *P. aeruginosa* purified EF-P. (E) Distribution of the diagnostic fragment ion [M + 2H-rhamnose]^2+^ normalized to the precursor ion intensity. **P < 0.01 (students *t*-test). All b- and y-type ions are annotated within 0.6 Da and represent the fragment containing the rhamnose glycan still attached *via* glycosidic linkage to the fragment ion.

To further confirm the α-configurational assignment of EF-P rhamnosylation, MS/MS analysis was performed on glycopeptides **14** and **15** bearing α- and β-configuration, respectively, and the data compared to glycopeptides produced from Lys-C digestion of isolated *P. aeruginosa* EF-P and the recombinant protein with *in vitro* EarP-transferred rhamnose. Subtle differences were observed in the fragmentation patterns between the two anomeric rhamnosylated glycopeptides **14** and **15** (Fig. 2B and C). This included a difference in the intensity of the doubly charged fragment arising from a loss of the rhamnose sugar from the triply charged precursor-ion under collision-induced dissociation (CID). The difference in stability of the α- verses β-linkage prompted us to investigate relative fragment ion distributions under CID MS/MS as a further tool to differentiate between the two anomers. The synthetic α- and β-rhamnosylated glycopeptides were analysed by CID and higher collisional dissociation (HCD) MS/MS with increasing normalised collision energies (NCEs), specifically 10–40 NCE with steps of 5 NCE. As expected, no fragment ion masses were unique to the α- or β-linkage with either fragmentation approach. However, we observed subtle differences in the intensity of the fragment-ion distributions. The intensity of the doubly charged fragment arising from a loss of the rhamnose sugar from the triply charged precursor-ion was the major ion significantly different for the two anomers with either CID or HCD. We hypothesise that this diagnostic fragment ion may arise from differences in the C–N bond strength at the anomeric centre. Specifically, due to back donation of electron density from the endocyclic oxygen into the σ* of the C1–N bond in the α-anomer (*i.e.* the endo anomeric effect) the C–N bond of this anomer would be expected to be longer and weaker than the corresponding β-anomer, thus leading to more extensive loss of rhamnose. The optimal fragmentation energy to maximize the intensity differences and distinguish between α- and β-anomers was 10–15% NCE and 30–40% NCE for HCD and CID, respectively (see Fig. 83, ESI†). However, the fragmentation efficiency of HCD at 15 NCE was only approximately 20% with the un-fragmented precursor ion being the major ion in MS/MS (see Fig. 84, ESI†). Therefore, all further work was performed with CID at 35 NCE which produces the maximal difference in the diagnostic fragment ion intensity between α- and β-anomers, and produces extensive peptide backbone fragmentation efficiency.

CID MS/MS analysis of the Lys-C glycopeptide fragment from isolated *P. aeruginosa* EF-P displayed very similar fragmentation patterns to the α-rhamnosylated glycopeptide **14** (Fig. 2D). Quantification of this fragment-ion intensity (normalized to the precursor-ion intensity) highlighted a significant difference between the α- and β-configured glycopeptides (Fig. 2E). The intensity of this fragment-ion from glycopeptides produced from *P. aeruginosa* EF-P and the *in vitro* rhamnosylated recombinant EF-P are clearly consistent with the α-configuration. Taken together, the NMR, UHPLC and tandem mass spectrometry data provide strong evidence that rhamnosylation of Arg-32 in *P. aeruginosa* EF-P (and by extension the conserved Arg residue of other bacterial EF-P proteins^21^) is α-configured.

## Conclusions

N-Linked rhamnosylation has recently been uncovered as an unusual modification of a conserved Arg residue in bacterial EF-P proteins that is critical for its role in preventing ribosome stalling during polyproline translation. In this work we have described an efficient synthetic route to suitably protected α- and β-rhamnosylated arginine ‘cassettes’ that can be directly incorporated into Fmoc-SPPS for the preparation of rhamnosylated peptides. We also describe the synthesis of Lys-C proteolytic fragments of *P. aeruginosa* EF-P bearing both α- and β-rhamnosylated arginine. Through a combination of 2D NMR and nano-UHPLC coupled to tandem mass spectrometry and using isolated and recombinant *P. aeruginosa* EF-P as standards we provide evidence that strongly suggests that the native protein bears an α-configured rhamnose residue on Arg-32. These findings complement a recent anomeric coupling constant analysis on the full length *S. oneidensis* homologue.^21^ Finally, we demonstrate that the α-rhamnosylated arginine moiety can undergo unusual endocyclic ring opening anomerization to the β-anomer. As such, synthetic or recombinant glycopeptides and glycoproteins bearing α-rhamnosyl arginine must be carefully handled to prevent unwanted loss of configurational purity. Importantly, the work described here lays the foundation for future synthetic and biochemical studies on bacterial glycopeptides and glycoproteins, including those bearing the rhamnosylated arginine motif, with a view to understanding the importance of glycosylation for bacterial protein structure and function.
