# Supplementary material for: Synthesis of rhamnosylated arginine glycopeptides and determination of the glycosidic linkage in bacterial elongation factor P
Source: Chem Sci. 2016 Dec 12;8(3):2296–302. doi: 10.1039/c6sc03847f (PMC5363394; doi:10.1039/c6sc03847f)
Supplement: Supplementary file 1 [file SC-008-C6SC03847F-s001.pdf]

Electronic Supplementary Material (ESI) for Chemical Science.
This journal is © The Royal Society of Chemistry 2016

# Supplementary Information

## Synthesis of Rhamnosylated Arginine Glycopeptides and Determination of the Glycosidic Linkage in Bacterial Elongation Factor P

Siyao Wang, Leo Corcilius, Patrick P. Sharp, Andrei Rajkovic, Michael Ibba, Benjamin L. Parker and Richard J. Payne*

School of Chemistry, The University of Sydney, NSW 2006, Australia

* Corresponding author. Tel.: +61-2-9351-5877; fax: +61-2-9351-3329; e-mail: richard.payne@sydney.edu.au.

# Table of Contents

**1. General experimental procedures** **S4**

**2. Synthesis and Characterisation of Rhamnosylated Arginine Building Blocks 1 and 2** **S5**

2,3,4-tri-*O*-acetyl-6-deoxy-α-L-mannopyranos-1-yl chloride **6** S5

2,3,4-tri-*O*-acetyl-6-deoxy-α-L-mannopyranos-1-yl isothiocyanate **3** S5

1-(2,2,4,6,7-Pentamethyldihydrobenzofuran-5-sulfonyl)-3-(2,3,4-tri-O-acetyl-6-deoxy-α-L-mannopyranos-1-yl)-2-ethylisothiourea **8** S6

*N*α-[(9*H*-fluoren-9-yl)-methoxycarbonyl]-*N*ω-(2,2,4,6,7-pentamethyldihydrobenzofuran-5-sulfonyl)-*N*ω’(2,3,4-tri-*O*-acetyl-6-deoxy-α-L-mannopyranos-1-yl)-L-arginine Allyl Ester **10** S10

*N*α-[(9*H*-fluoren-9-yl)-methoxycarbonyl]-*N*ω-(2,2,4,6,7-pentamethyldihydrobenzofuran-5-sulfonyl)-*N*ω’-(2,3,4-tri-*O*-acetyl-6-deoxy-α-L-mannopyranos-1-yl)-L-arginine **1** S14

*N*α-[(9*H*-fluoren-9-yl)-methoxycarbonyl]-*N*ω-(2,2,4,6,7-pentamethyldihydrobenzofuran-5-sulfonyl)-*N*ω’-(6-deoxy-α-L-mannopyranos-1-yl)-L-arginine trifluoroacetate salt **13** S18

2,3,4-tri-*O*-acetyl-6-deoxy-α-L-mannopyranosyl azide **7** S23

2,3,4-tri-*O*-acetyl-6-deoxy-β-L-mannopyranos-1-yl isothiocyanate **4** S25

1-(2,2,4,6,7-Pentamethyldihydrobenzofuran-5-sulfonyl)-3-(2,3,4-tri-*O*-acetyl-6-deoxy-β-L-mannopyranos-1-yl)-2-ethylisothiourea **9** S29

*N*α-[(9*H*-fluoren-9-yl)-methoxycarbonyl]-*N*ω-(2,2,4,6,7-pentamethyldihydrobenzofuran-5-sulfonyl)-*N*ω’(2,3,4-tri-*O*-acetyl-6-deoxy-β-L-mannopyranos-1-yl)-L-arginine Allyl Ester **11** S33

*N*α-[(9*H*-fluoren-9-yl)-methoxycarbonyl]-*N*ω-(2,2,4,6,7-pentamethyldihydrobenzofuran-5-sulfonyl)-*N*ω’(2,3,4-tri-*O*-acetyl-6-deoxy-β-L-mannopyranos-1-yl)-L-arginine **2** S37

*N*α-[(9*H*-fluoren-9-yl)-methoxycarbonyl]-*N*ω-(2,2,4,6,7-pentamethyldihydrobenzofuran-5-sulfonyl)-*N*ω’(2,3,4-tri-*O*-acetyl-6-deoxy- β -L-mannopyranos-1-yl)-L-arginine trifluoroacetate salt **12** S41

**3. General Procedures for Peptide Synthesis** **S46**

3.1 Preloading of Chemmatrix® Trtyl-OH Resin S46

3.2 Fmoc Deprotection S46

3.3 Coupling of General Amino Acid Building Blocks S46

3.4 Coupling of Rhamnosylated Arginine Building Blocks S46

3.5 Capping S46

3.6 Deprotection of α-Rhamnosylated Peptide S47

3.7 Deprotection of β-Rhamnosylated Peptide S47

**4. Synthesis and Characterization of Rhamnosylated Peptides** **S48**

4.1 α-Rhamnosylated Peptide **14** S48

4.2 β-Rhamnosylated Peptide **15** S52

4.3 Resolution of Glycopeptides **14** and **15** with HPLC system S56

**5. Analysis of Anomerization under Acidic and Basic Conditions** **S58**

5.1 Analysis of **1** and **2** during conventional Pbf-deprotection and deacetylation sequence S58

5.2 Anomerization of α-Rhamnosylated Glycopeptide during Deacetylation with Hydrazine (off resin) S61

5.3 Anomerization of α-Rhamnosylated Glycopeptide during Deacetylation with Hydrazine (on resin)

S62
5.4 Examination of β-Rhamnosylated Glycopeptide during Deacetylation with Hydrazine (off resin) S63
5.5 Trapping of Imine Intermediate during Glycopeptide Anomerization S65
5.6 Anomerization of α-Rhamnosylated Glycopeptide during Treatment with NaOMe. S66

**6. His tagged purification of Pseudomonas aeruginosa EF-P S68**

**7. *in vitro* rhamnosylation of EF-P. S68**

**8. Protease digestion and peptide preparation S69**

**9. nanoUHPLC-MS/MS S69**

**10. References S74**

# 1. General experimental procedures

All reactions were carried out under an argon atmosphere. Commercially available reagents were obtained from Sigma Aldrich, Merck, AK Scientifc Inc. or GL Biochem. All commercial materials were used as received without further purification unless otherwise noted. Flash chromatography was performed on silica gel (0.040 – 0.060 mm) manufactured by Grace. Solid-phase peptide synthesis (SPPS) was carried out in polypropylene syringes equipped with Teflon filters, purchased from Torviq. Dichloromethane was purchased from Merck. Peptide synthesis grade DMF was purchased from Labscan. Chemmatrix® Trtyl-OH resin was purchased from PCAS Biomatrix Inc.

All NMR spectra were recorded at 300 K using a Bruker Avance DRX400 or DRX500 spectrometer. Chemical shifts are reported in parts per million (ppm) and are referenced to solvent residue signals.[1] Proton assignments were made with the assistance of COSY, HSQC and HMBC spectra. Anomeric configurations were assigned on the basis of 2D NOESY or 1D selective ROESY NMR spectra (H1-H3 and H1-H5 NoEs were present for β anomers and absent for α anomers)[2] and $J_{C1\text{-}H1}$ values ($J_{C1\text{-}H1}$ ≈ 170 Hz for α and ≈ 160 Hz for β)[3], which were obtained from $^{1}H$-$^{13}C$ HSQC spectra without proton decoupling. For 2D NOESY and HSQC spectra contours, black represents positive cross peaks and red represents negative cross peaks.

Infrared (IR) absorption spectra were recorded on a Bruker ALPHA Spectrometer with Attenuated Total Reflection (ATR) capability using OPUS 6.5 software. Compounds were deposited as films on the ATR plate *via* solid compression. Optical rotations were recorded at ambient temperature (293K) on a Perkin–Elmer 341 polarimeter at 589 nm (sodium D line) with a cell path length of 1 dm, and the concentrations are reported in g/100 mL.

Ultra-pressure liquid chromatography-mass spectrometry (UPLC-MS) was performed on a Shimadzu Nexera X2 UPLC-MS 2020 system using a LC-30AD Pump and a SPD-M30A detector. High Resolution ESI mass spectra were obtained on a Bruker Apex Qe 7T Fourier Transform Ion Cyclotron Resonance Mass spectrometer equipped with a ESI/MALDI (Nd:YAG) dual source.

Preparative reverse-phase HPLC was performed on a Waters 2535 quaternary gradient module, equipped with a Waters 2489 UV detector operating at 214 nm using Waters Empower 3 software. All separations were performed on a Waters XBridge OBD C18 preparative column at a flow rate of 7 mL/min using a gradient from 100% water to 100% MeCN over 30 min (buffered with 0.1% TFA). Analytical HPLC analysis of glycopeptides **14** and **15** was performed on a Waters Atlantis® T3 C18 Column using a flow rate of 0.2 mL/min or ResTek Raptor fluorophenyl column with the flow rate as specified.

## 2. Synthesis and Characterisation of Rhamnosylated Arginine Building Blocks 1 and 2

2,3,4-tri-*O*-acetyl-6-deoxy-α-L-mannopyranos-1-yl chloride **6**

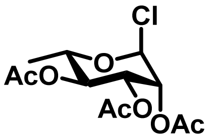

Rhamnosyl chloride **6** was prepared according to Wang *et. al.*[4] as described below.

Rhamnosyl acetate **5** (1.0 g, 3.0 mmol) was dissolved in dry dichloromethane (30 mL). The solution was cooled to 0 °C. Thionyl chloride (0.87 mL, 12.0 mmol) and stannic chloride (1 M in dichloromethane solution, 6.0 mL, 6.0 mmol) was added into the reaction. The resulting solution was stirred at 25 °C under argon for 5 h. After the reaction was complete (monitored by TLC), it was quenched by addition of saturated aqueous $NaHCO_3$ solution (30 mL). The organic layer was then separated; while the aqueous layer was extracted with dichloromethane (2 x 50 mL). The combined organic extracts were dried over $MgSO_4$ and then concentrated under reduced pressure. The crude residue was purified through flash chromatography (3:1 v/v *n*-hexane:ethyl acetate) to yield rhamnosyl chloride **6**. (0.73 g, 2.37 mmol, 80%). **$^1$H NMR** (400 MHz, $CDCl_3$): δ 5.91 (d, 1H, $J_{1,2}$ = 1.5 Hz, H-1), 5.54, (dd, 1H, $J_{2,3}$ = 3.4 Hz, $J_{3,4}$ = 10.2 Hz, H-3), 5.36 (dd, 1H, $J_{1,2}$ = 1.7 Hz, $J_{2,3}$ = 3.4 Hz, H-2), 5.11 ($t_{apt}$, 1H, $J_{3,4}$ = $J_{4,5}$ = 10.1 Hz, H-4), 4.14 (dq, 1H, $J_{4,5}$ = 9.9 Hz, $J_{5,6}$ = 6.2 Hz, H-5), 2.14 (s, 3H, OAc), 2.05 (s, 3H, OAc), 1.98 (s, 3H, OAc), 1.24 (d, 3H, $J_{5,6}$ = 6.2 Hz, H-6) ppm. **$^{13}$C NMR** (100 MHz, $CDCl_3$): δ 167.0, 169.9, 169.8, 89.2, 72.0, 70.5, 69.6, 67.9, 20.9, 20.8, 20.7, 17.2 ppm. These data are in agreement with those previously reported by Wang *et al.*[4]

2,3,4-tri-*O*-acetyl-6-deoxy-α-L-mannopyranos-1-yl isothiocyanate **3**

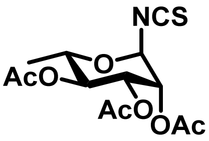

Rhamnosyl isothiocyanate **3** was prepared in a similar manner to that reported by Li *et. al.*[5]

A suspension of KSCN (0.53 g, 5.5 mmol), TBAI (1.01 g, 2.73 mmol) and activated powdered 4Å molecular sieves in MeCN (10 mL) was stirred at 25 °C under argon for 16 h. A solution of rhamnosyl chloride **6** (0.73 g, 2.73 mmol) in MeCN (15 mL) was added into the reaction. The mixture was heated at reflux for 2 h. The mixture was then filtered through celite and the filtrate was concentrated under vacuum. The

residue was purified through column chromatography (3:1 v/v *n*-hexane:ethyl acetate) to yield rhamnosyl-α-isothiocyanate **3** as white solid following lyophilization (0.61 g, 1.82 mmol, 67%). $[\alpha]_D$ -7.7 (c 30, acetone). **IR:** 2014 (NCS), 1749 (C=O) $cm^{-1}$. **$^1$H NMR** (500 MHz, d6-Acetone): δ 5.74 (d, 1H, $J_{1,2}$ = 1.9 Hz, H-1), 5.34, (dd, 1H, $J_{1,2}$ = 1.9 Hz, $J_{2,3}$ = 3.4 Hz, H-2), 5.22, (dd, 1H, $J_{2,3}$ = 3.4 Hz, $J_{3,4}$ = 10.2 Hz, H-3), 5.03, ($t_{apt}$, 1H, $J_{3,4}$ = $J_{4,5}$ = 10.2 Hz, H-4), 4.02, (dq, 1H, $J_{4,5}$ = 10.1 Hz, $J_{5,6}$ = 6.2 Hz, H-5), 2.11 (s, 3H, OAc), 2.05 (s, 3H, OAc), 1.94 (s, 3H, OAc), 1.23 (d, 3H, $J_{5,6}$ = 6.2 Hz, H-6) ppm. **$^{13}$C NMR** (125 MHz, d6-Acetone): δ 169.5, 169.4, 169.4, 141.7, 83.4, 70.0, 69.8, 69.7, 68.5, 20.0, 19.9, 19.8, 16.9 ppm. **HRMS**: (+ESI) m/z, $[M + Na]^+$, Calcd: 354.0619, Found: 354.0618.

1-(2,2,4,6,7-Pentamethyldihydrobenzofuran-5-sulfonyl)-3-(2,3,4-tri-O-acetyl-6-deoxy-α-L-mannopyranos-1-yl)-2-ethylisothiourea **8**

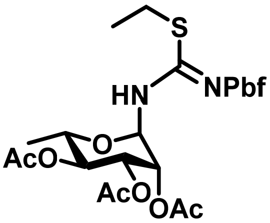


A solution of Pbf-$NH_2$ (147 mg, 0.55 mmol) and KO*t*Bu (60 mg, 0.51 mmol) in anhydrous THF (20 mL) was stirred under argon for 1 h. A solution of rhamnosyl isothiocyanate **3** (140 mg, 0.43 mmol) in THF (10 mL) was added dropwise into the mixture. After 3 h, the solution was neutralized with Amberlite® IR-120 ion exchange resin. After filtration, the filtrate was concentrated under reduced pressure. The crude mixture dissolved in anhydrous MeCN under argon. Anhydrous $K_2CO_3$ (0.60 g, 4.3 mmol) was added into the reaction followed by EtI (89 µL, 1.5 mmol). The mixture was stirred at 25 °C for 16 h. After the reaction was complete (monitored by LCMS), the mixture was concentrated and purified through flash chromatography with a gradient from 2:1 v/v *n*-hexane:ethyl acetate to 1:2 v/v *n*-hexane:ethyl acetate yielding the desired isothiourea **8** (210 mg, 0.42 mmol, 80%) as a white foam. $[\alpha]_D$ -1.9 (c 1.2, acetone). **IR:** 3263 (N-H), 1748 (C=O), 1369 (S=O), 1155 (S=O) $cm^{-1}$. **$^1$H NMR** (500 MHz, d6-Acetone): δ 8.71 (br, NH), 5.41 (dd, 1H, $J_{1,2}$ = 2.8 Hz, $J_{1,NH}$ = 7.5 Hz, H-1), 5.31 ($t_{apt}$, 1H, $J_{1,2}$ = $J_{2,3}$ = 3.0 Hz, H-2), 5.06 (dd, 1H, $J_{2,3}$ = 3.1 Hz, $J_{3,4}$ = 9.0 Hz, H-3), 5.00 ($t_{apt}$, 1H, $J_{3,4}$ = $J_{4,5}$ = 8.9 Hz, H-4), 3.74 ($quintet_{apt}$, 1H, $J_{4,5}$ = $J_{5,6}$ = 6.7 Hz, H-5), 3.05 (s, 2H, Ar-C$\underline{H_2}$), 3.00 (m, 2H, S-C$\underline{H_2}$-$CH_3$), 2.59 (s, 3H, Ar-C$\underline{H_3}$), 2.53 (s, 3H, Ar-C$\underline{H_3}$), 2.11 (s, 3H, OAc), 2.09 (s, 3H, Ar-C$\underline{H_3}$), 2.08 (s, 3H, OAc), 1.99 (s, 3H, OAc), 1.47 (s, 3H, C-C$\underline{H_3}$), 1.46 (s, 3H, C-C$\underline{H_3}$), 1.22 (m, 6H, $J_{5,6}$ = 6.7 Hz, H-6 and S-$CH_2$-C$\underline{H_3}$) ppm. **$^{13}$C NMR** (125 MHz, d6-Acetone): δ 170.3, 170.2 (2 C),168.0, 160.1, 139.6, 133.8, 132.5, 126.2, 118.3, 87.7, 79.7, 71.1, 69.5, 69.1, 43.4, 28.7, 26.4, 20.7, 20.6, 20.5, 19.4, 18.2, 17.6, 14.4, 12.4 ppm. **HRMS**: (+ESI) m/z, $[M + H]^+$, Calcd: 629.2197, Found: 629.2202

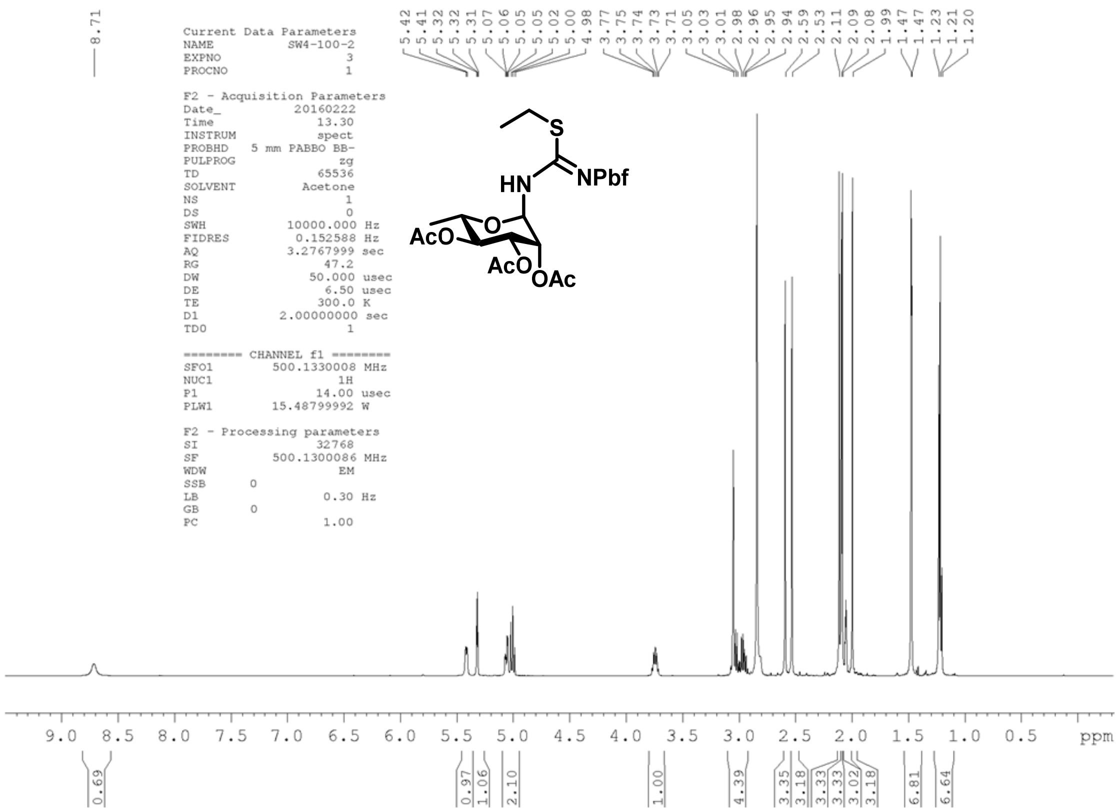


Figure 1: $^{1}$H NMR spectrum for **8**

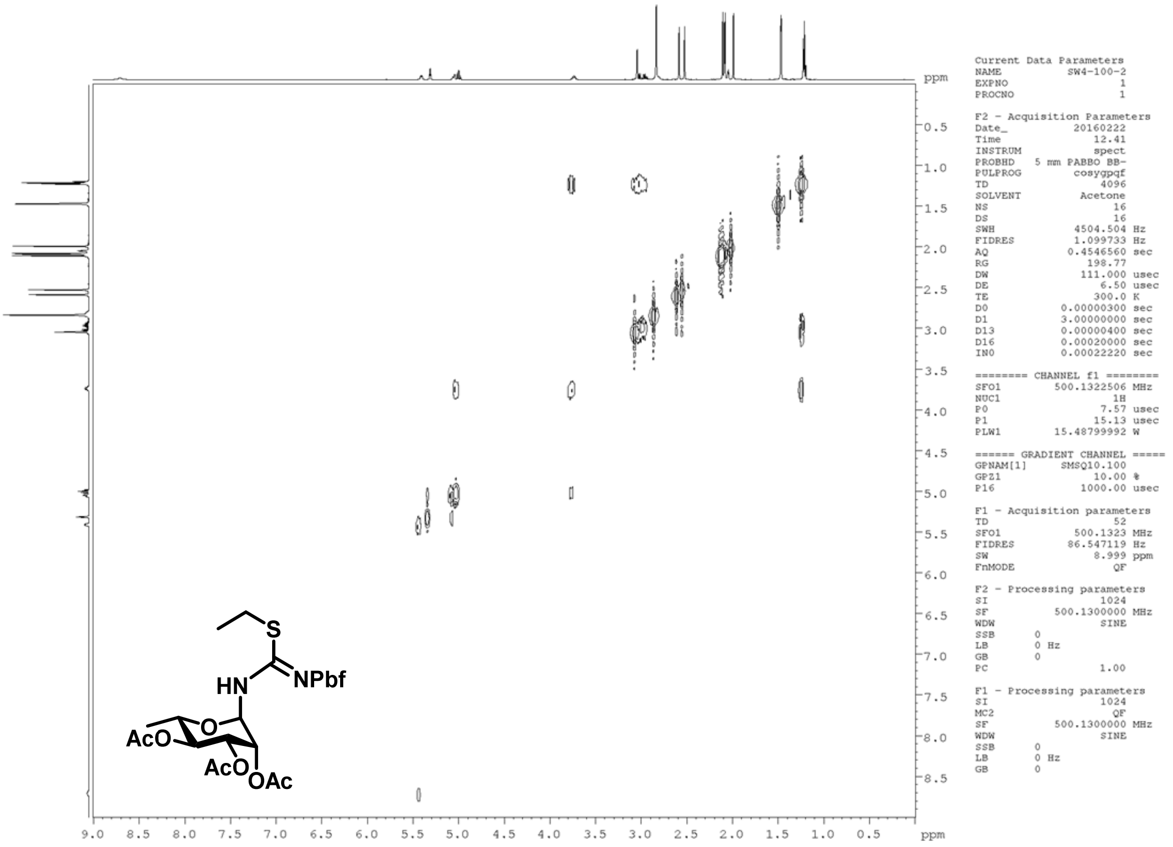


Figure 2: COSY NMR spectrum for **8**

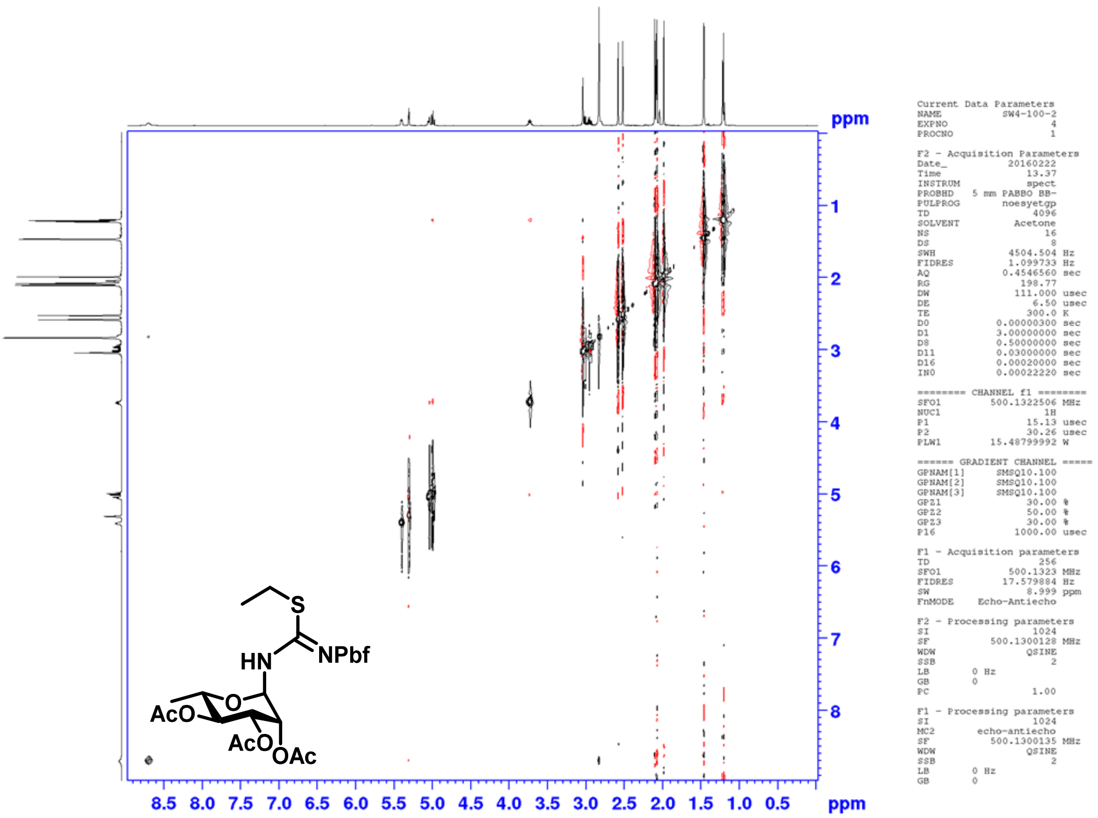


Figure 3: NOESY NMR spectrum for **8**

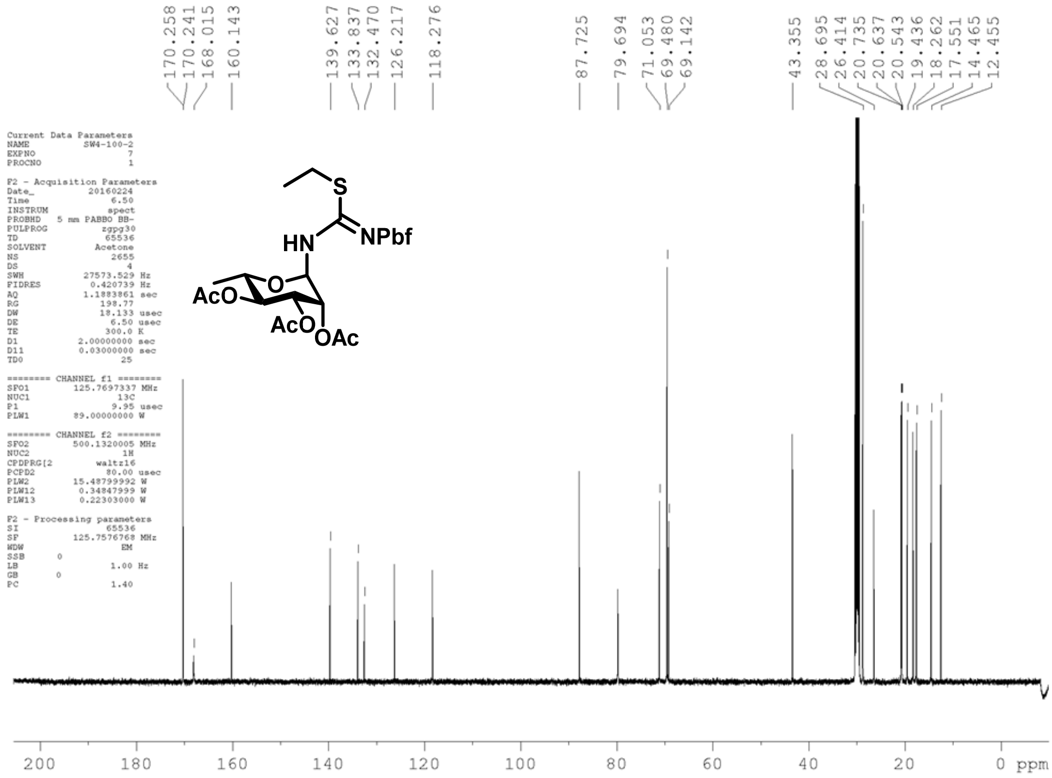


Figure 4: $^{13}C$ NMR spectrum for **8**

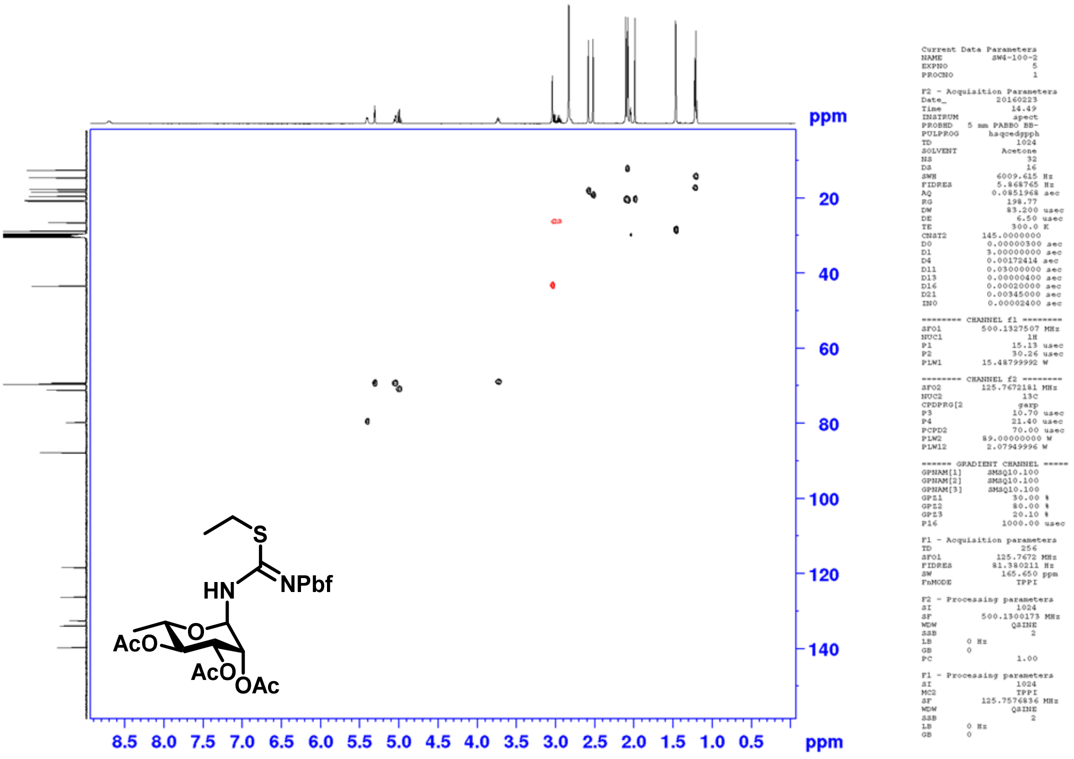

Figure 5: $^{1}H$-$^{13}C$ HSQC NMR spectrum for **8**

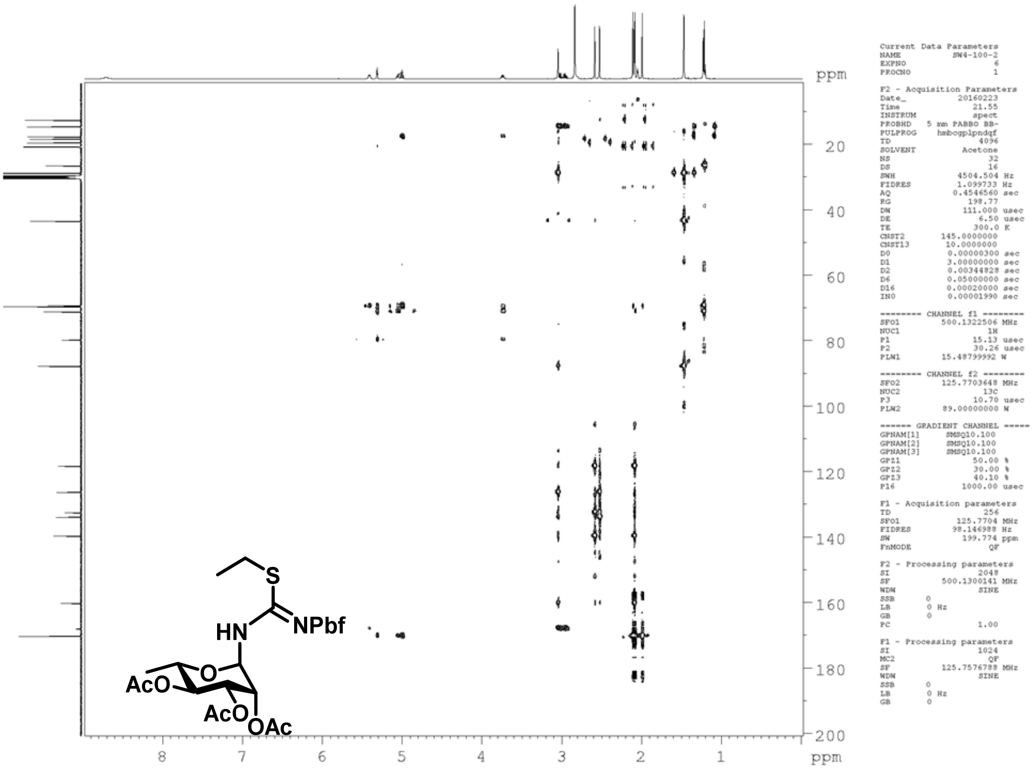

Figure 6: $^{1}H$-$^{13}C$ HMBC NMR spectrum for **8**

*N*α-[(9*H*-fluoren-9-yl)-methoxycarbonyl]-*N*ω-(2,2,4,6,7-pentamethyldihydrobenzofuran-5-sulfonyl)-*N*ω'(2,3,4-tri-*O*-acetyl-6-deoxy-α-L-mannopyranos-1-yl)-L-arginine Allyl Ester **10**

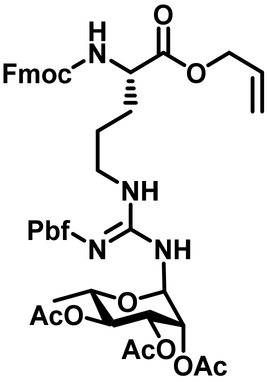


To a solution of isothiourea **8** (0.172 g, 0.27 mmol), Fmoc-Orn-OAll (0.240 g, 0.6 mmol) and $NEt_3$ (140 μL, 1.0 mmol) in chloroform (10 mL) was added $HgCl_2$ (0.11 g, 0.40 mmol). The mixture was stirred at 25 °C for 2 h. After completion (monitored by TLC), the mixture was filtered through a pad of Celite®. The filtrate was concentrated and purified through flash chromatography (2:1 v/v ethyl acetate:*n*-hexane) to yield the desired α-rhamnosylated arginine **10** (0.233 g, 0.24 mmol, 90%). $[\alpha]_D$ -17 (c 0.65, acetone). **IR:** 3344 (N-H), 1745 (C=O), 1369 (S=O) $cm^{-1}$. **$^1$H NMR** (400 MHz, d6-Acetone): δ 7.87 (d, 2H, *J* = 7.5 Hz, Ar-H), 7.72 (d, 2H, *J* = 7.3 Hz, Ar-H), 7.43 ($t_{apt}$, 2H, *J* = 7.4 Hz, Ar-H), 7.34 ($t_{apt}$, 2H, *J* = 7.5 Hz, Ar-H), 6.86 (d, 1H, *J* = 8.0 Hz, Fmoc-NH), 5.95 (m, 1H, O-$CH_2$-C<u>H</u>=$CH_2$), 5.34 (dq, 1H, $J_1$ = 17.3 Hz, $J_2$ = 1.6 Hz, O-$CH_2$-CH=C$\underline{H_2}$), 5.30 (b, 1H, H-2), 5.21 (dq, 1H, $J_1$ = 10.5 Hz, $J_2$ = 1.4 Hz O-$CH_2$-CH=C$\underline{H_2}$), 5.10-5.02 (b, 2H, H-3, H-4), 4.62 (d, 2H, *J* = 5.4 Hz, O-C$\underline{H_2}$-CH=$CH_2$), 4.36 (m, 2H, *J* = 7.0 Hz, FmocCH-C$\underline{H_2}$-O-CO), 4.30 (m, 1H, Arg-Hα,) 4.26 (m, 1H, = 7.0 Hz, FmocC<u>H</u>-$CH_2$-O-CO), 3.77 (b, 1H, H-5), 3.42 (m, 2H, Arg-Hδ), 3.01 (s, 2H, Ar-C$\underline{H_2}$), 2.64 (s, 3H, Ar-C$\underline{H_3}$), 2.57 (s, 3H, Ar-C$\underline{H_3}$), 2.10 (3H, OAc), 2.09 (s, 3H, Ar-C$\underline{H_3}$), 2.07 (s, 3H, OAc), 1.99 (s, 3H, OAc), 1.91 (m, 1H, Arg-Hβ1), 1.77 (m, 1H, Arg-Hβ2), 1.75 (m, 2H, Arg-Hγ), 1.45 (s, 6H, C-(C$\underline{H_3}$)$_2$), 1.22 (d, 3H, $J_{5,6}$ = 6.3 Hz, H-6) ppm. **$^{13}$C NMR** (100 MHz, d6-Acetone): 171.7, 169.4, 169.3, 158.4, 156.2, 144.2, 144.1, 141.2, 138.0, 133.9, 132.4, 132.2, 127.7, 127.1, 125.3, 125.2, 124.8, 119.9, 117.4, 116.9, 86.3, 78.3, 70.1, 68.8, 68.7, 67.1, 66.3, 65.1, 53.8, 47.1, 42.7, 40.6, 27.9, 25.6, 19.9, 19.7, 19.7, 18.7, 17.5, 16.9, 11.7 ppm. **HRMS**: (+ESI) m/z, $[M + Na]^+$, Calcd: 983.3719, Found: 983.3713

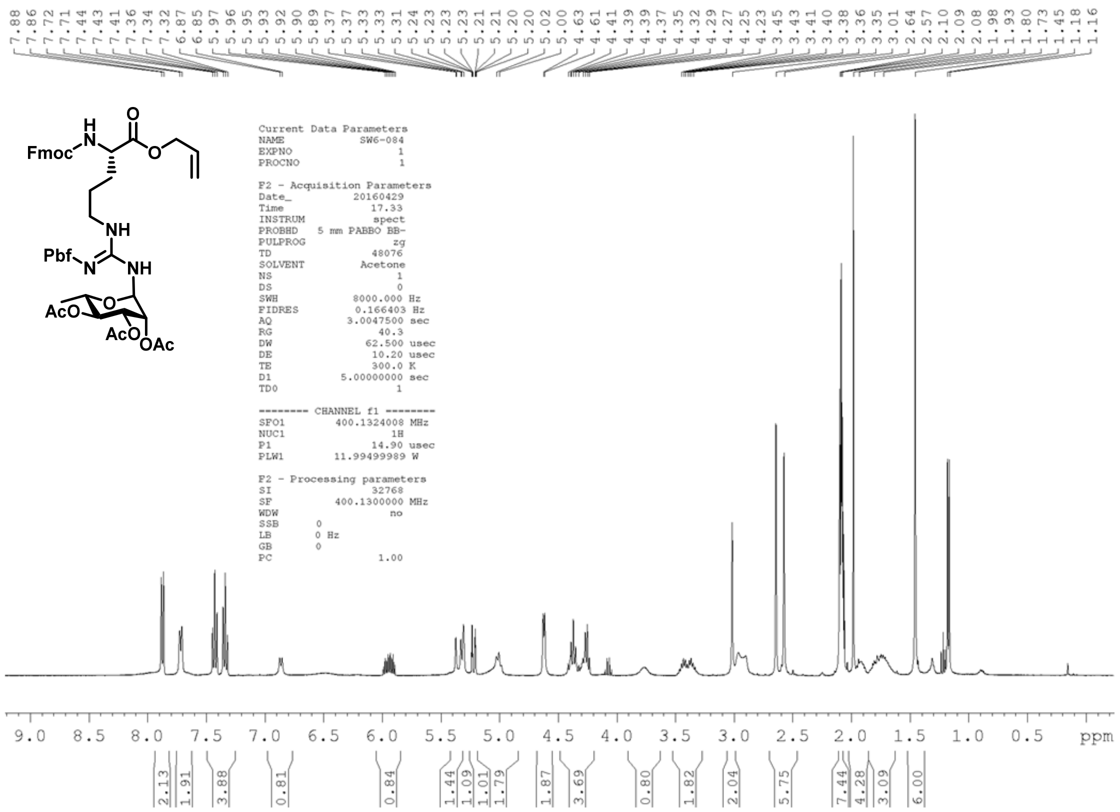


Figure 7: $^{1}$H NMR spectrum for **10**

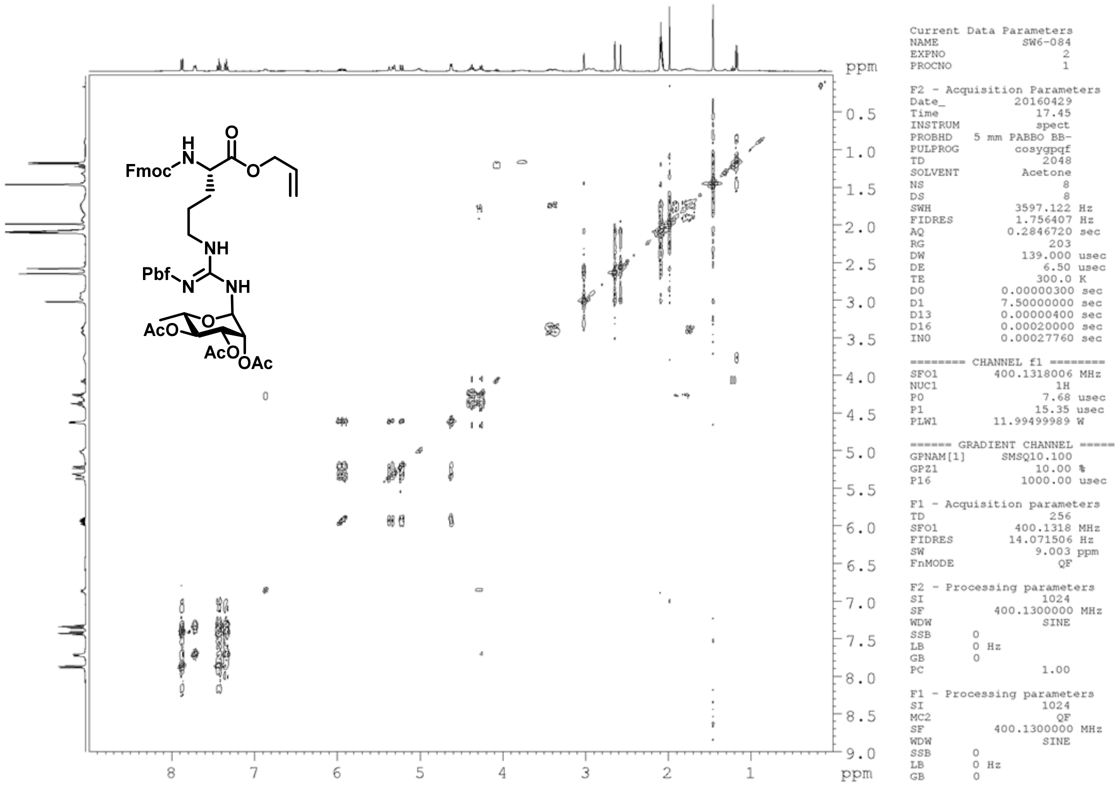


Figure 8: COSY NMR spectrum for **10**

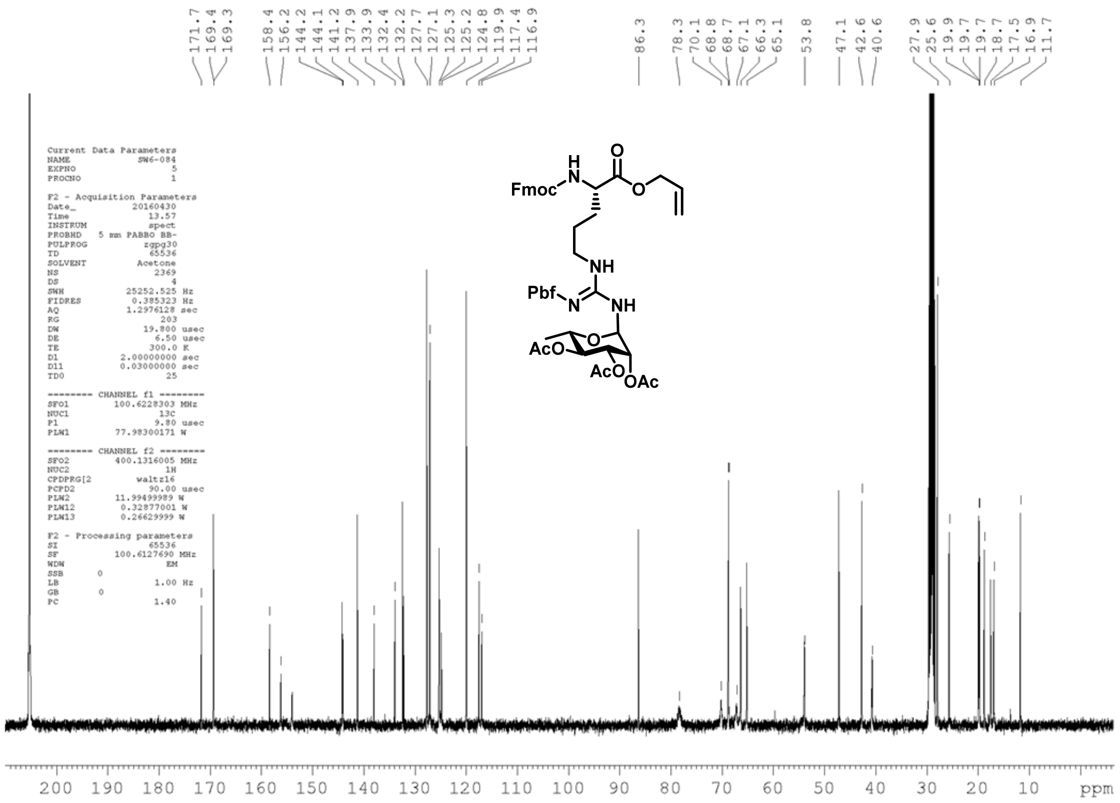

Figure 9: $^{13}$C NMR spectrum for **10**

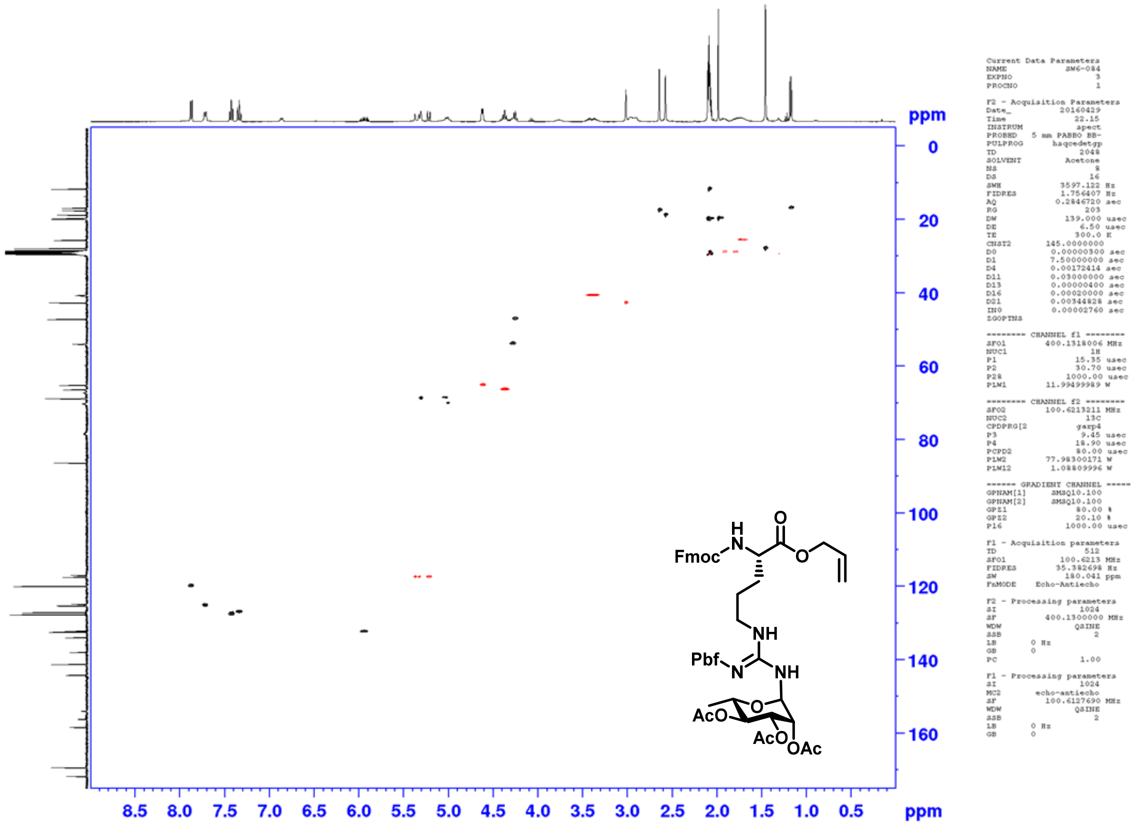

Figure 10: $^{1}$H-$^{13}$C HSQC NMR spectrum for **10**

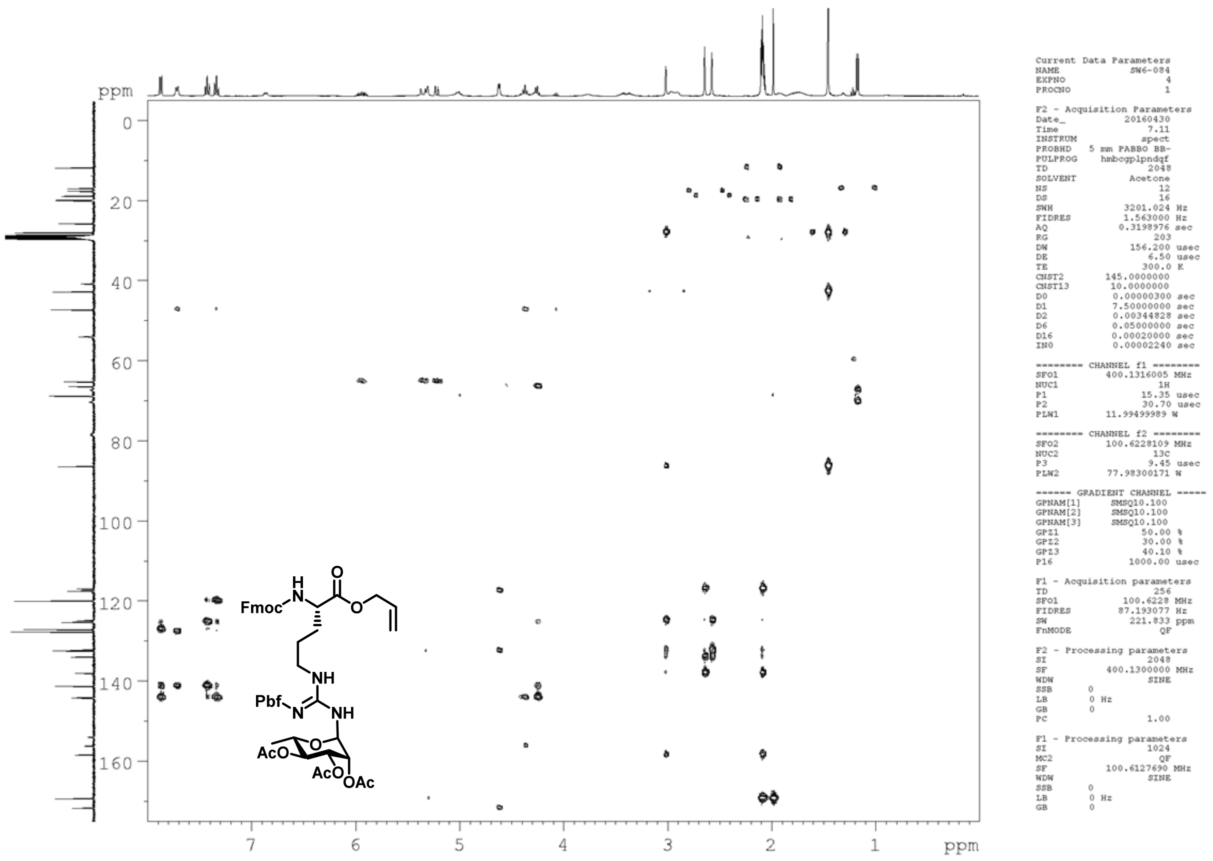


Figure 11: $^{1}$H-$^{13}$C HMBC NMR spectrum for **10**

*N*α-[(9*H*-fluoren-9-yl)-methoxycarbonyl]-*N*ω-(2,2,4,6,7-pentamethyldihydrobenzofuran-5-sulfonyl)-*N*ω'-(2,3,4-tri-*O*-acetyl-6-deoxy-α-L-mannopyranos-1-yl)-L-arginine **1**

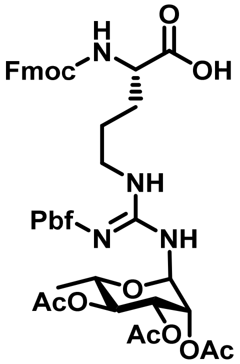


The allyl ester **10** (150 mg, 0.16 mmol) was dissolved in anhydrous THF (10 mL). $Pd(PPh_3)_4$ (150 mg, 0.13 mmol) was added to the solution followed by phenylsilane (0.2 mL, 1.5 mmol). The mixture was stirred at 25 °C for 30 min. After the reaction was complete (monitored by TLC), the solution was concentrated under reduced pressure. The mixture was then filtered through a pad of silica with 1% AcOH in ethyl acetate as eluent. The combined filtrate was decolorized over activated charcoal, concentrated and purified through reverse phase HPLC to yield the desired α-rhamnosylated arginine building block **1** (78 mg, 0.084 mmol, 52%) as a white solid following lyophilization. $[\alpha]_D$ -15 (c 0.73, acetone). **IR:** 3332 (N-H), 1747 (C=O), 1369 (S=O), 1166 (S=O) $cm^{-1}$. **$^1$H NMR** (400 MHz, $CD_3OD$): δ 7.79 (d, 2H, *J* = 8.66 Hz, Ar-H), 7.68 (dd, 2H, $J_1$ = 7.3 Hz, $J_2$ = 3.08 Hz, Ar-H), 7.38 ($t_{apt}$, 2H, *J* = 7.3 Hz, Ar-H), 7.29 (tdd, 2H, $J_1$ = 7.3 Hz, $J_2$ = 3.08 Hz, $J_3$ = 1.1 Hz, Ar-H), 5.26 (m, 2H, Rhm-H), 4.94, (m, 2H, Rhm-H), 4.35 (m, 2H, *J* = 7.3 Hz, FmocCH-C$\underline{H_2}$-O-CO), 4.23 (m, 1H, *J* = 7.0 Hz, FmocC$\underline{H}$-$CH_2$-O-CO), 4.11 (m, 1H, Arg-Hα), 3.52 (b, 1H, H-5), 3.31 (m, 2H, Arg-Hδ), 2.97 (s, 2H, Ar-C$\underline{H_2}$), 2.60 (s, 3H, Ar-C$\underline{H_3}$), 2.54 (s, 3H, Ar-C$\underline{H_3}$), 2.10-2.03 (m, 9H, 2 OAc and Ar-C$\underline{H_3}$), 1.98 (s, 3H, OAc), 1.85 (m, 1H, Arg-Hβ1), 1.68 (m, 1H, Arg-Hβ2), 1.60 (m, 2H, Arg-Hγ), 1.42 (s, 6H, C-(C$\underline{H_3}$)$_2$), 1.11 (d, 3H, $J_{5,6}$ = 6.2 Hz, H-6) ppm. **$^{13}$C NMR** (100 MHz, $CD_3OD$): 171.5, 171.4, 160.2, 158.6, 145.4, 145.2, 142.6, 139.5, 133.8, 128.8, 128.2, 126.3, 120.9, 118.8, 87.9, 70.3, 70.0, 68.0, 55.4, 43.8, 42.1, 30.1, 28.8, 26.9, 20.8, 20.5, 19.7, 18.6, 17.7, 12.5 ppm. **HRMS**: (+ESI) m/z, $[M + H]^+$, Calcd: 921.3587, Found: 921.3581

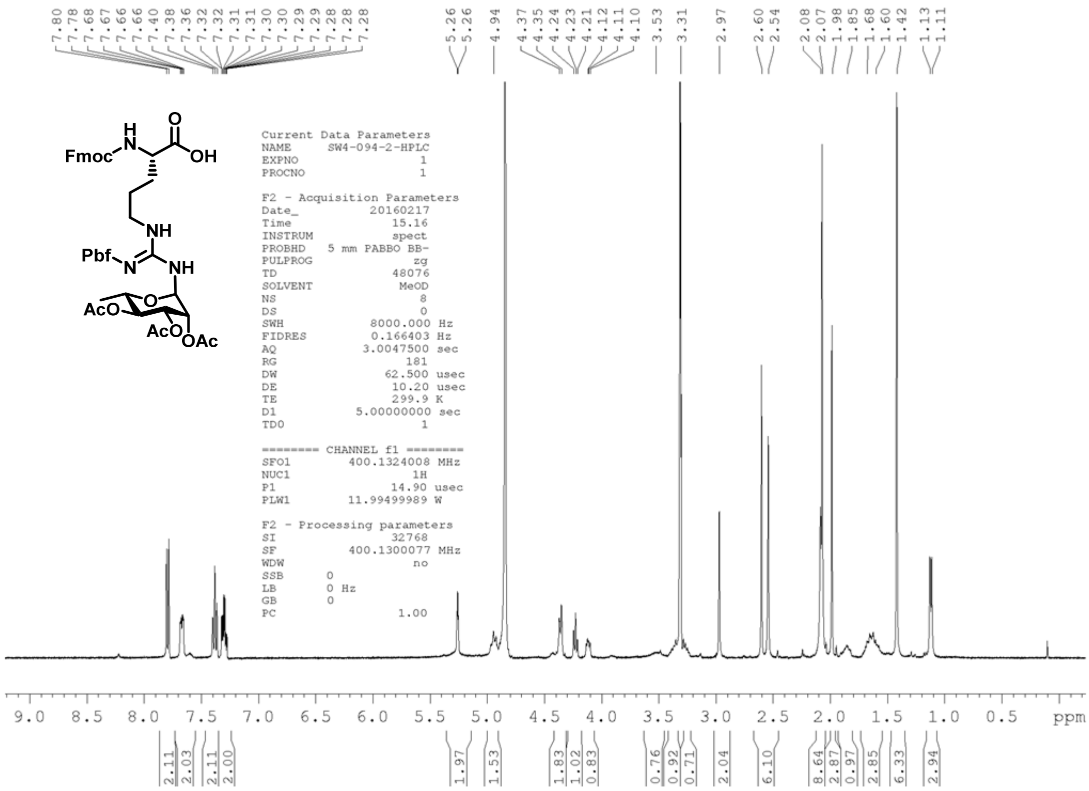


Figure 12: $^{1}$H NMR spectrum for **1**

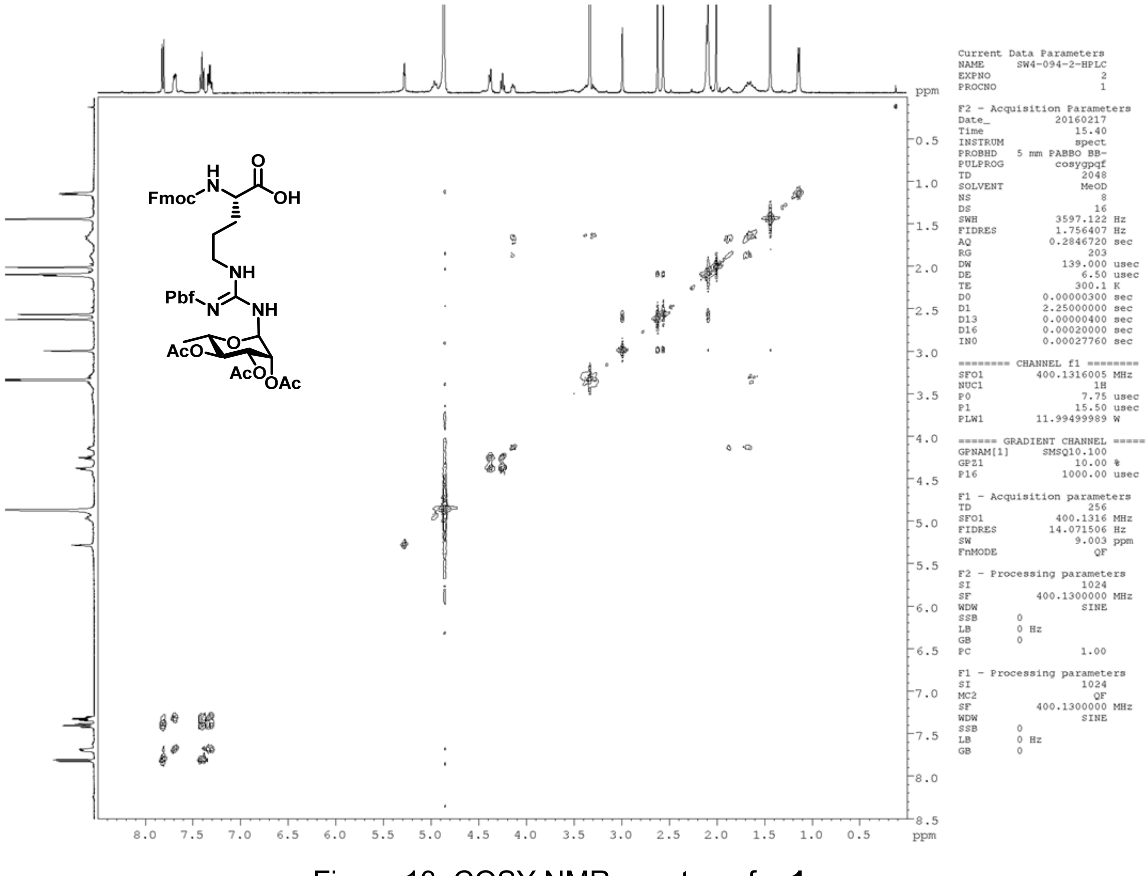


Figure 13: COSY NMR spectrum for **1**

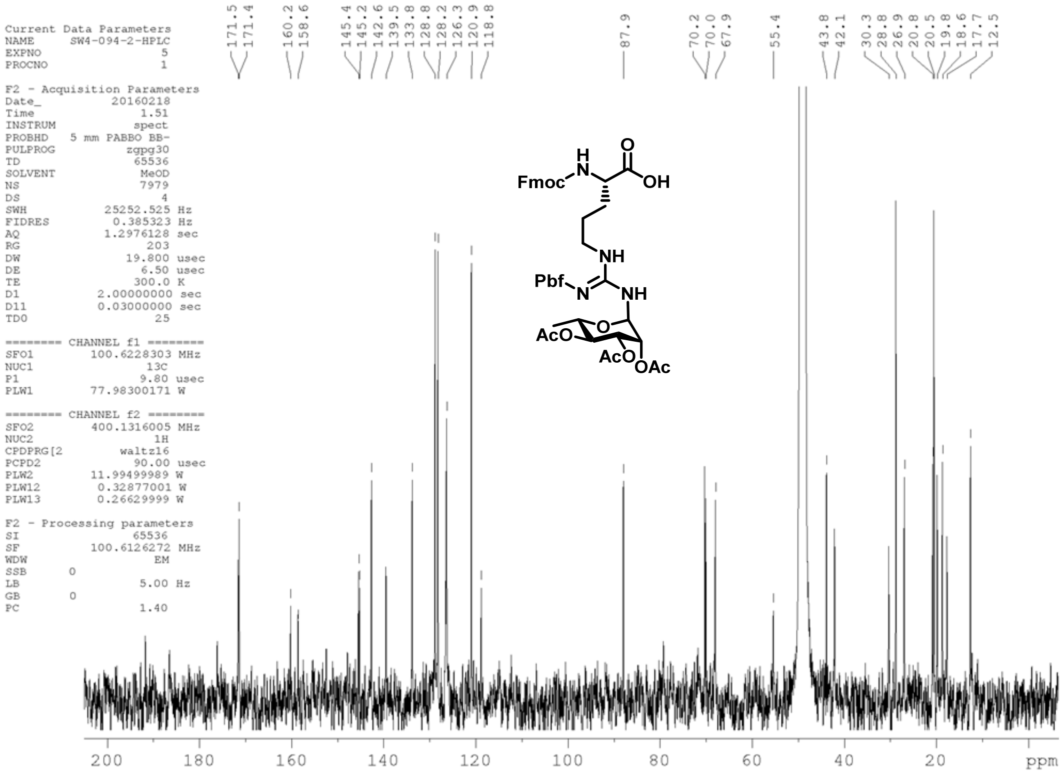


Figure 14: $^{13}$C NMR spectrum for **1**

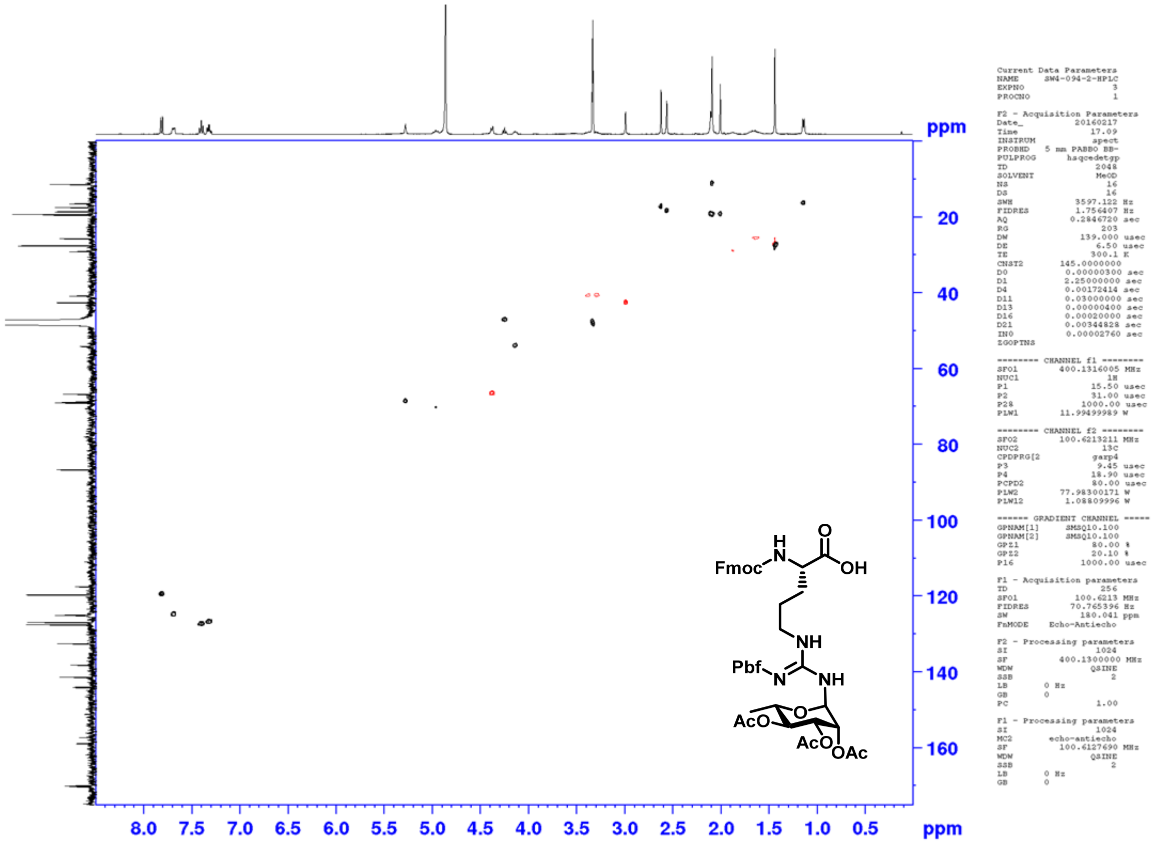


Figure 15: $^{1}$H-$^{13}$C HSQC NMR spectrum for **1**

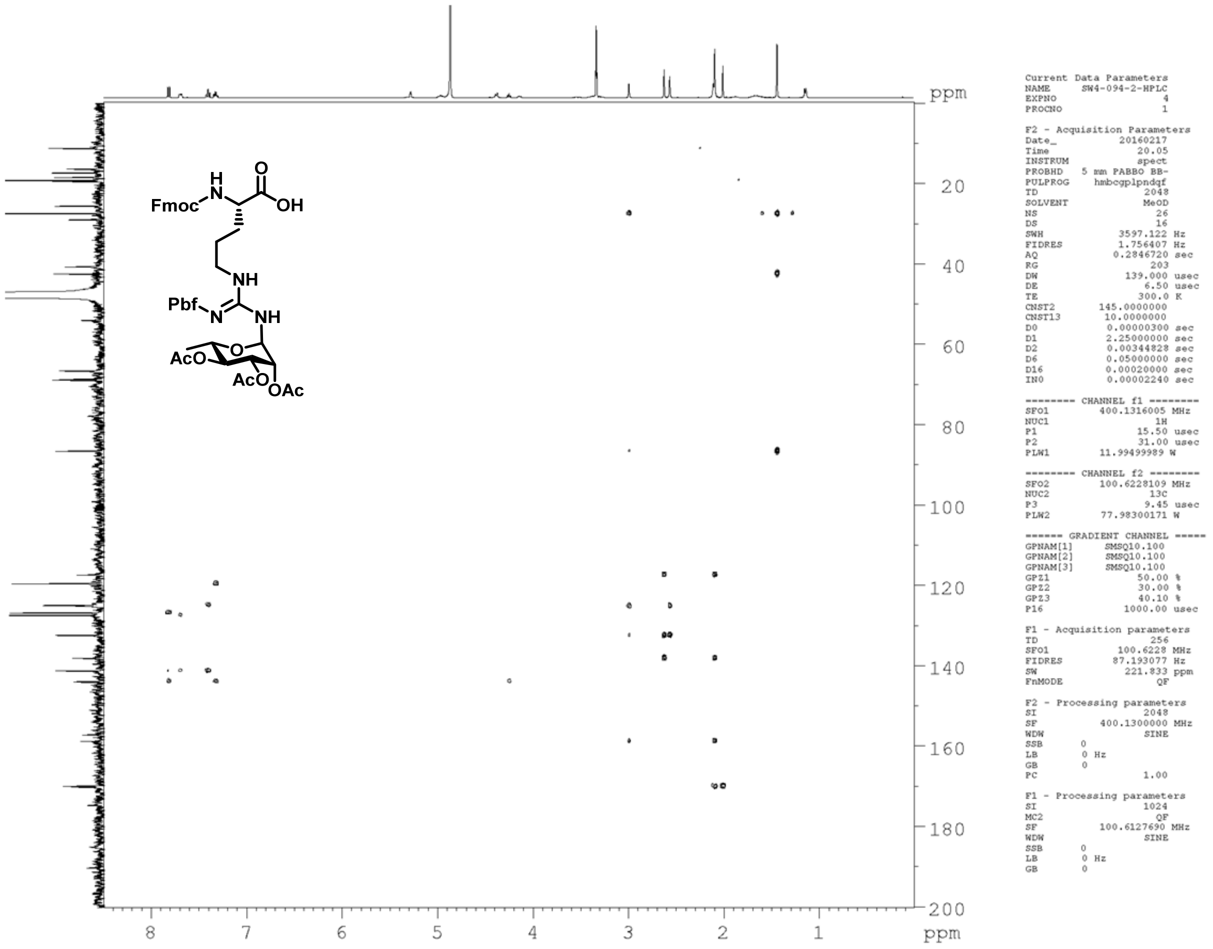


Figure 16: $^{1}H$-$^{13}C$ HMBC NMR spectrum for **1**

*N*α-[(9*H*-fluoren-9-yl)-methoxycarbonyl]-*N*ω-(2,2,4,6,7-pentamethyldihydrobenzofuran-5-sulfonyl)-*N*ω'-(6-deoxy-α-L-mannopyranos-1-yl)-L-arginine trifluoroacetate salt **13**

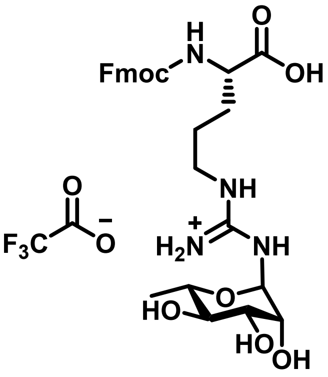


Rhammosylated arginine building block **1** (100 mg, 0.11 mmol) was dissolved in dry MeOH (5 mL). NaOMe (0.5 M in MeOH, 0.32 mL, 0.16 mmol) was added into the solution to obtain a final pH of ~8 (as judged by moist universal indicator strips). The mixture was stirred at 25 °C. After 30 min, the reaction was judged to be complete (UPLC-MS monitoring). The solution was adjusted to pH 4 by careful addition of TFA before concentrating under reduced pressure. The residue was then suspended in TFA/*i*-$Pr_3SiH/H_2O$ (18:1:1 v/v/v, 10 mL) at 25 °C with vigorous mixing. After 90 min, the mixture was concentrated and then purified through RP-HPLC to afford deprotected α-rhamnosylated arginine **13** as a white solid following lyophilization (45 mg, 0.053 mmol, 45%). $[\alpha]_D$ -1.2 (c 9.5, methanol). **IR:** 3360 (N-H, O-H), 1678 (C=O) $cm^{-1}$. **$^1$H NMR** (500 MHz, $CD_3OD$): δ 7.80 (d, 2H, *J* = 7.4 Hz, Ar-H), 7.66 ($t_{apt}$, 2H, *J* = 8.7 Hz, Ar-H), 7.39 ($t_{apt}$, 2H, *J* = 7.5 Hz, Ar-H), 7.31 ($t_{apt}$, 2H, *J* = 7.5 Hz, Ar-H), 5.11 (d, 1H, *J* = 1.8 Hz, H-1), 4.40 (m, 2H, FmocCH-C<u>H<sub></sub></u>$\underline{H_2}$-O-CO), 4.23 (m, 1H, *J* = 7.0 Hz, FmocC$\underline{H}$-$CH_2$-O-CO), 4.20 (m, 1H, Arg-Hα), 3.88 (m, 1H, H-2), 3.60 (dd, 1H, $J_{2,3}$ = 3.2 Hz, $J_{3,4}$ = 8.7 Hz, H-3), 3.50 (dq, 1H, $J_{4,5}$ = 9.1 Hz, $J_{5,6}$ = 6.2 Hz, H-5), 3.43 ( $t_{apt}$, $J_{3,4}$ = $J_{4,5}$ = 9.1 Hz, H-4), 3.28 (m, 2H, Arg-Hδ), 1.93 (m, 1H, Arg-Hβ1), 1.73 (m, 1H, Arg-Hβ2), 1.68 (m, 2H, Arg-Hγ), 1.28 (d, 3H, $J_{5,6}$ = 5.8 Hz, H-6) ppm. **$^{13}$C NMR** (125 MHz, $CD_3OD$): 174.0, 158.0 (q, $\underline{C}OCF_3$), 157.4, 156.3, 143.9, 143.7, 141.2, 127.4, 126.8, 126.7, 124.8, 124.8, 119.5, 115.4 (q, $\underline{C}F_3$), 81.4 ($J_{C1-H1}$ = 167 Hz), 72.2, 70.6, 69.8, 69.6, 66.7, 66.6, 53.4, 40.9, 28.5, 24.9, 16.6 ppm. **HRMS**: (+ESI) m/z, $[M + H]^+$, Calcd: 543.2446, Found: 543.2448

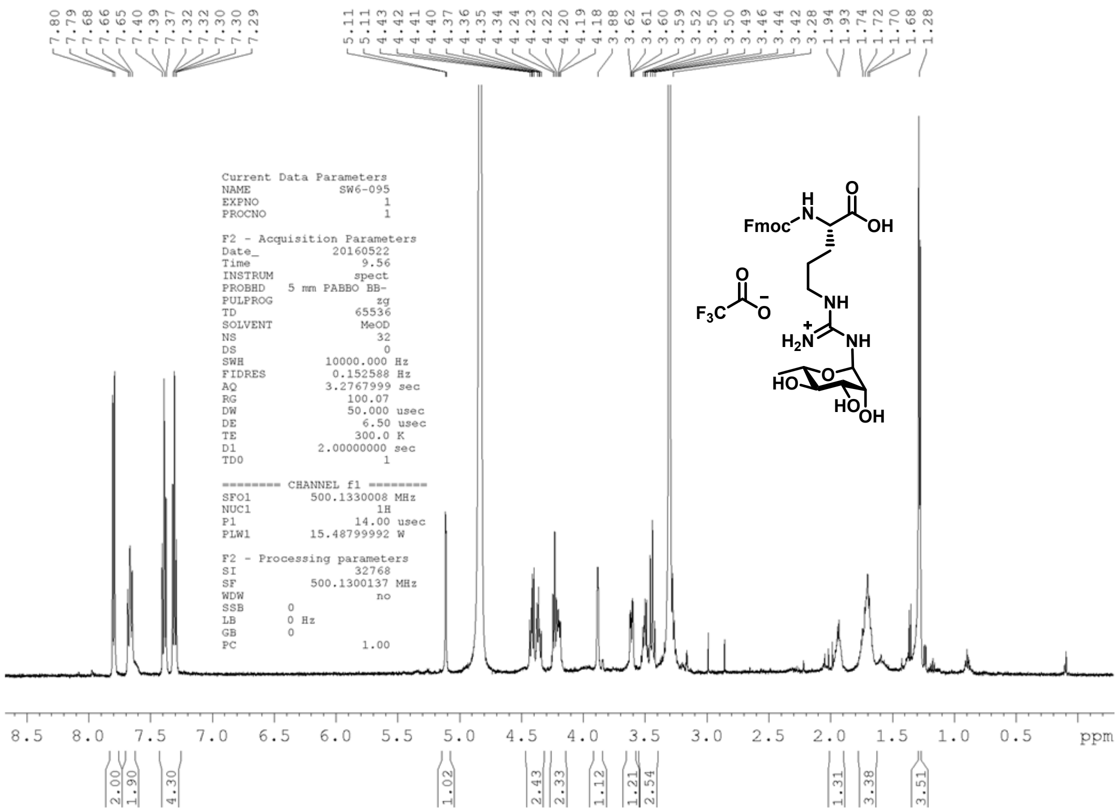

Figure 17: $^{1}$H NMR spectrum for **13**

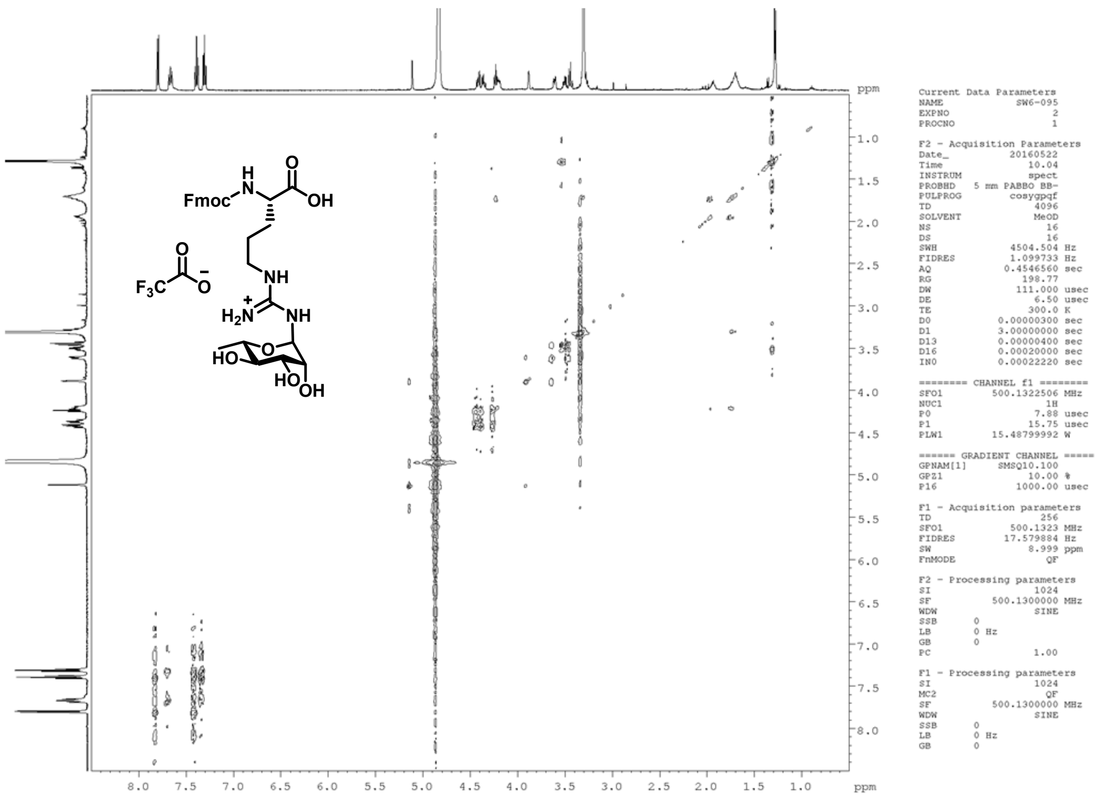

Figure 18: COSY NMR spectrum for **13**

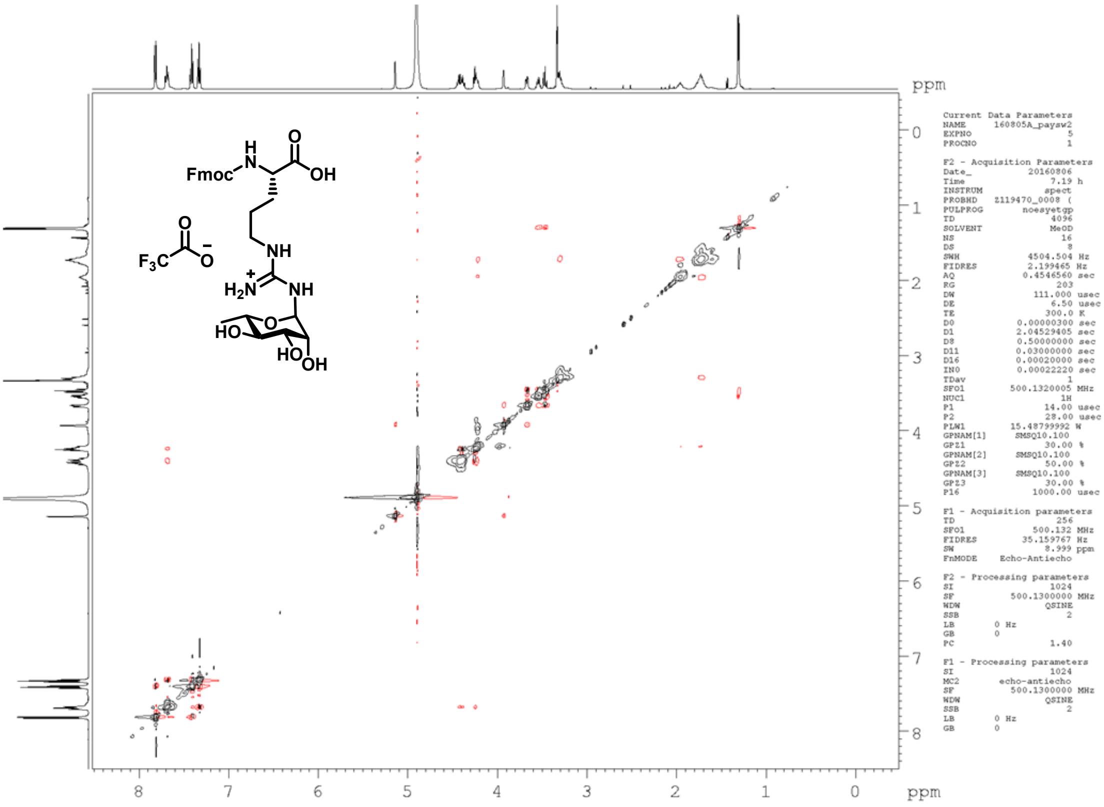


Figure 19: NOESY NMR spectrum for **13**

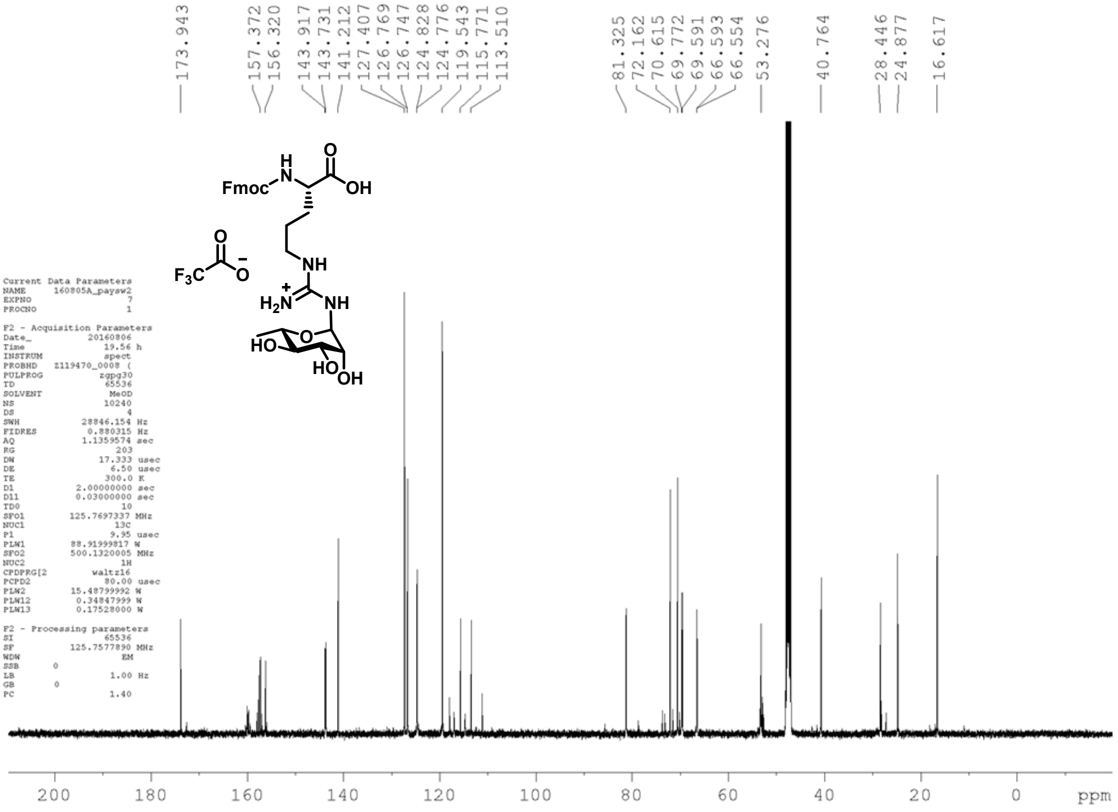


Figure 20: $^{13}$C NMR spectrum for **13**

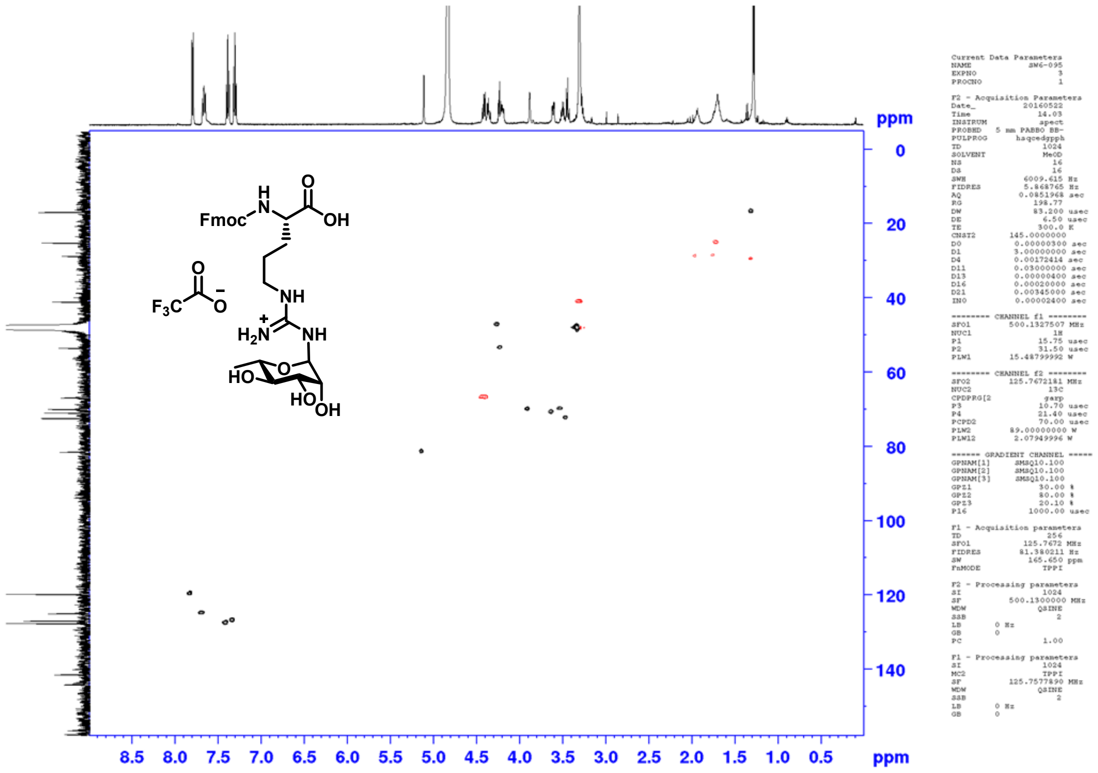

Figure 21: $^{1}H$-$^{13}C$ HSQC NMR spectrum for **13**

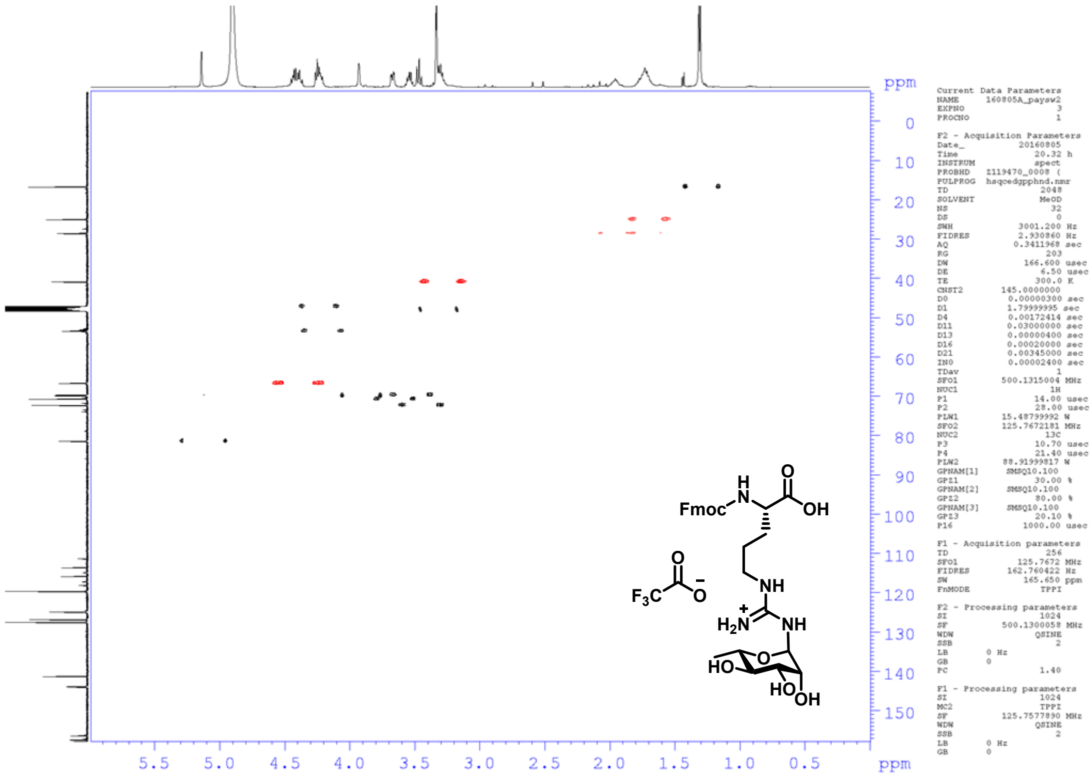

Figure 22: $^{1}H$-$^{13}C$ HSQC without proton decoupling NMR spectrum for **13**

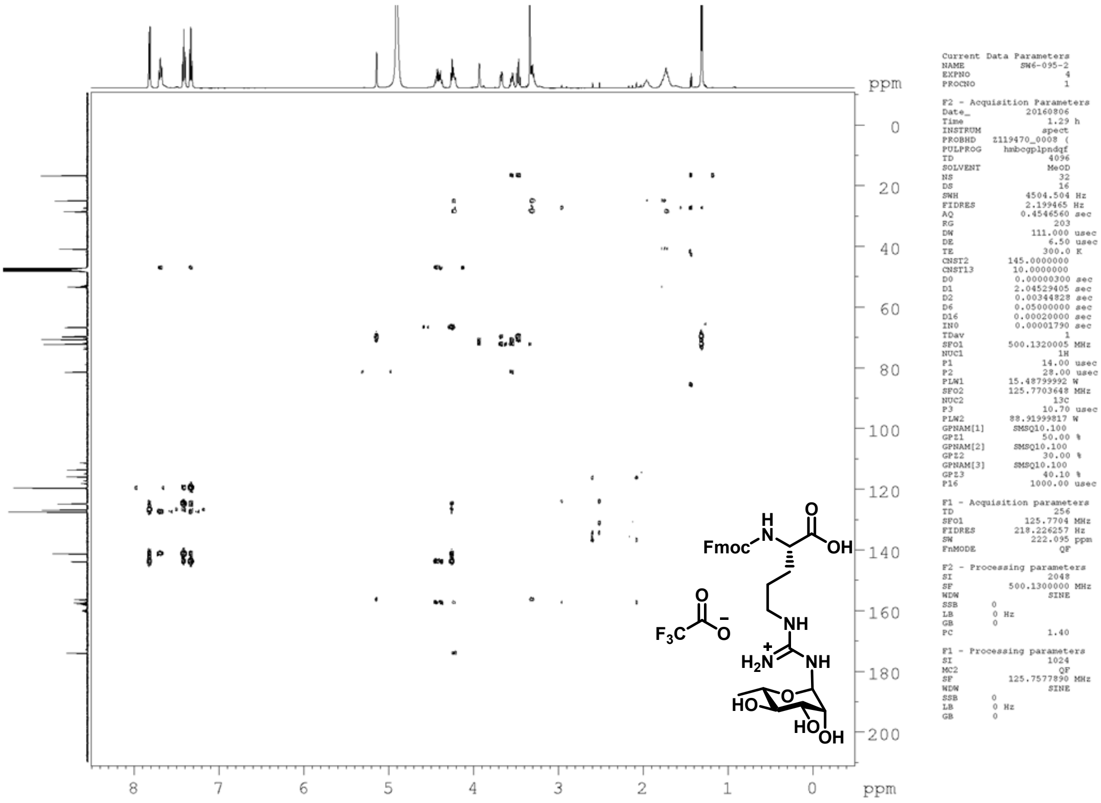


Figure 23: $^1H$-$^{13}C$ HMBC NMR spectrum for **13**

2,3,4-tri-*O*-acetyl-6-deoxy-α-L-mannopyranosyl azide **7**

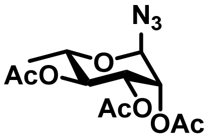


Rhamnosyl acetate **5** (1.0 g, 3.0 mmol) was dissolved in dry dichloromethane (30 mL). After cooling to 0 °C, $TMSN_3$ (0.87 mL, 12.0 mmol) and stannic chloride (1 M in dichloromethane solution, 6.0 mL, 6.0 mmol) were added to the reaction. The resulting solution was stirred at 25 °C under argon for 1 h. After the reaction was complete (monitored by TLC), it was quenched through addition of saturated $NaHCO_3$ solution (30 mL). The organic layer was then separated and the aqueous layer was extracted with dichloromethane (2 x 50 mL). The combined organic extracts were dried over $MgSO_4$ and then concentrated under reduced pressure. The residue was purified through flash chromatography (3:1 v/v *n*-hexane:ethyl acetate) to yield rhamnosyl azide **7** (0.73 g, 2.37 mmol, 80%). $[\alpha]_D$ -110 (c 2.66, acetone). **IR:** 2112 ($N_3$), 1745 (C=O) $cm^{-1}$. **$^1$H NMR** (400 MHz, $CDCl_3$): δ 5.30 (d, 1H, $J_{1,2}$ = 1.9 Hz, H-1), 5.20 (dd, 1H, $J_{2,3}$ = 3.4 Hz, $J_{3,4}$ = 10.0 Hz, H-3), 5.14 (dd, 1H, $J_{1,2}$ = 1.9 Hz, $J_{2,3}$ = 3.4 Hz, H-2), 5.03 ($t_{apt}$, 1H, $J_{3,4}$ = $J_{4,5}$ = 10.0 Hz, H-4), 4.03 (dq, 1H, $J_{4,5}$ = 9.6 Hz, $J_{5,6}$ = 6.2 Hz, H-5), 2.15 (3H, OAc), 2.05 (3H, OAc), 1.98 (3H, OAc), 1.27 (d, 3H, $J_{5,6}$ = 6.2 Hz, H-6) ppm. **$^{13}$C NMR** (100 MHz, $CDCl_3$): δ 169.9, 169.8, 169.8, 87.5, 70.5, 69.5, 68.6, 68.3, 20.8, 20.7, 20.6, 17.4 ppm. **HRMS**: (+ESI) m/z, $[M + Na]^+$, Calcd: 338.0959, Found: 338.0962.

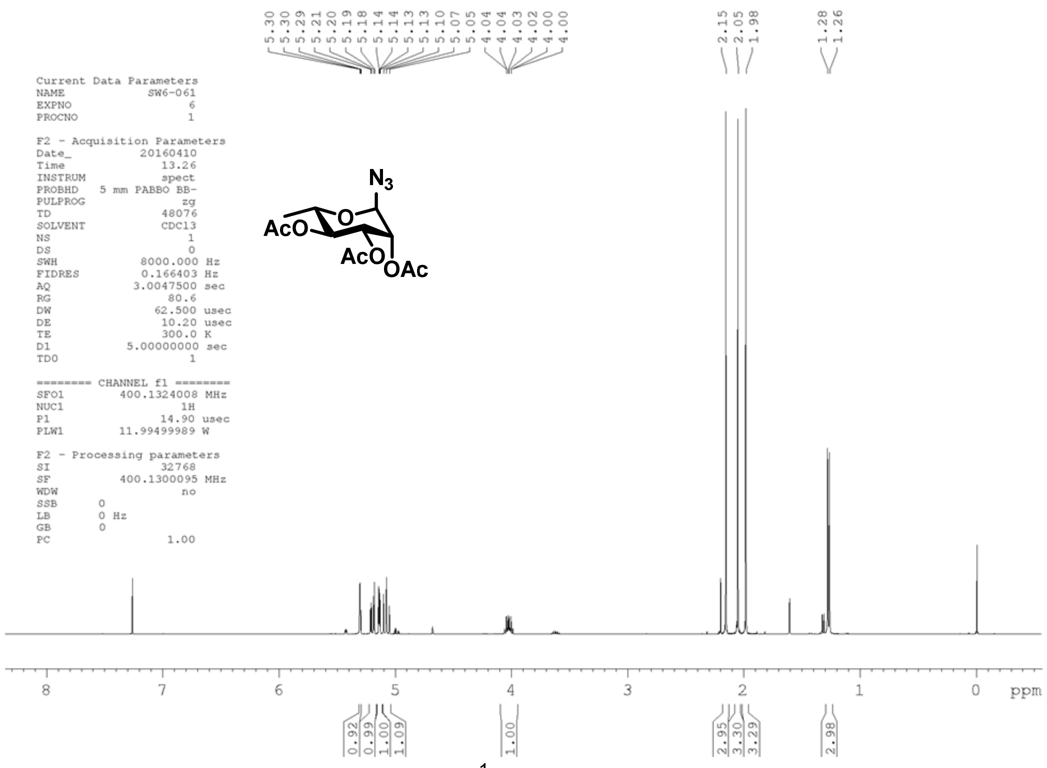


Figure 24: $^{1}$H NMR spectrum for **7**

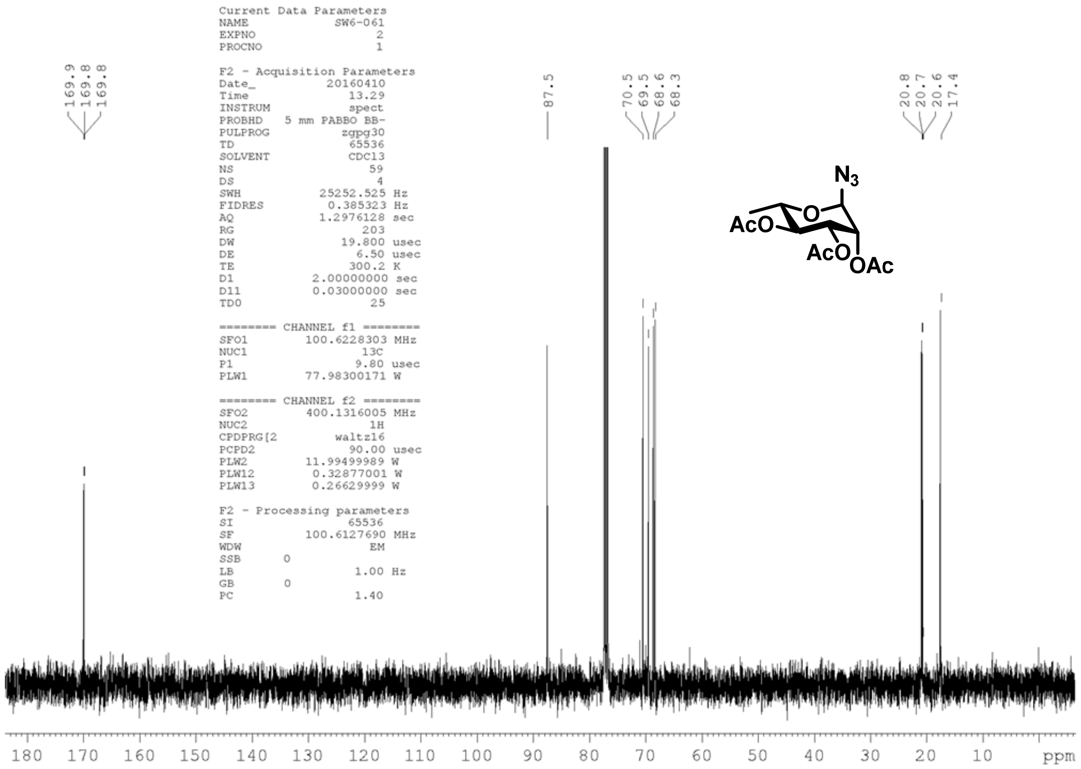


Figure 25: $^{13}$C NMR spectrum for **7**

2,3,4-tri-*O*-acetyl-6-deoxy-β-L-mannopyranos-1-yl isothiocyanate **4**

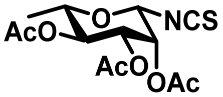


Rhamnosyl azide **7** (0.92 g, 2.85 mmol) was dissolved in MeOH. Pd (10% on charcoal, 0.1 g) was added to the solution. The mixture was stirred under hydrogen (1 atm) at 25 °C for 30 min. After the reaction was complete (monitored by TLC), the mixture was filtered through a pad of Celite®. The filtrate was concentrated and redissolved in ethyl acetate (10 mL). $H_2O$ (10 mL) and $CaCO_3$ (1.1 g, 11.1 mmol) was added into the reaction, followed by the dropwise addition of thiophosgene (436 µL. 5.7 mmol). The resulting mixture was stirred at 25 °C for 10 min. After the reaction was complete (monitored by TLC), the reaction was quenched by addition of saturated aqueous $NaHCO_3$ solution (10 mL) with vigorous stirring for 20 min. The mixture was then diluted with ethyl acetate (50 mL). The organic layer was separated and washed with $H_2O$ (3 x 25 mL), brine (25 mL), dried over $MgSO_4$ and concentrated. The resulting residue was purified through flash chromatography with a gradient from 5:1 v/v *n*-hexane:ethyl acetate to 3:1 v/v *n*-hexane:ethyl acetate to yield rhamnosyl-β-isothiocyanate **4** (0.53 g, 1.6 mmol, 60%). $[\alpha]_D$ +4.2 (c 24, acetone). **IR:** 2016 (NCS), 1742 (C=O) $cm^{-1}$. **$^1$H NMR** (500 MHz, d6-Acetone): δ 5.50 (dd, 1H, $J_{1,2}$ = 1.6 Hz, $J_{2,3}$ = 3.4 Hz, H-2), 5.36 (d, 1H, $J_{1,2}$ = 1.6 Hz, H-1), 5.14 (dd, 1H, $J_{2,3}$ = 3.4 Hz, $J_{3,4}$ = 10.2 Hz, H-3), 4.97 ($t_{apt}$, 1H, $J_{3,4}$ = $J_{4,5}$ = 10.0 Hz, H-4), 3.81 (dq, 1H, $J_{4,5}$ = 10.1 Hz, $J_{5,6}$ = 6.2 Hz, H-5), 2.18 (s, 3H, OAc), 2.02 (s, 3H, OAc), 1.92 (s, 3H, OAc), 1.22 (d, 3H, $J_{5,6}$ = 6.2 Hz, H-6) ppm. **$^{13}$C NMR** (125 MHz, d6-Acetone): δ 169.7, 169.5, 169.4, 143.3, 82.7, 72.5, 70.5, 69.9, 69.8, 20.0, 19.9, 19.7, 16.9 ppm. **HRMS** (+ESI) m/z, $[M + Na]^+$, Calcd: 354.0618, Found: 354.0623

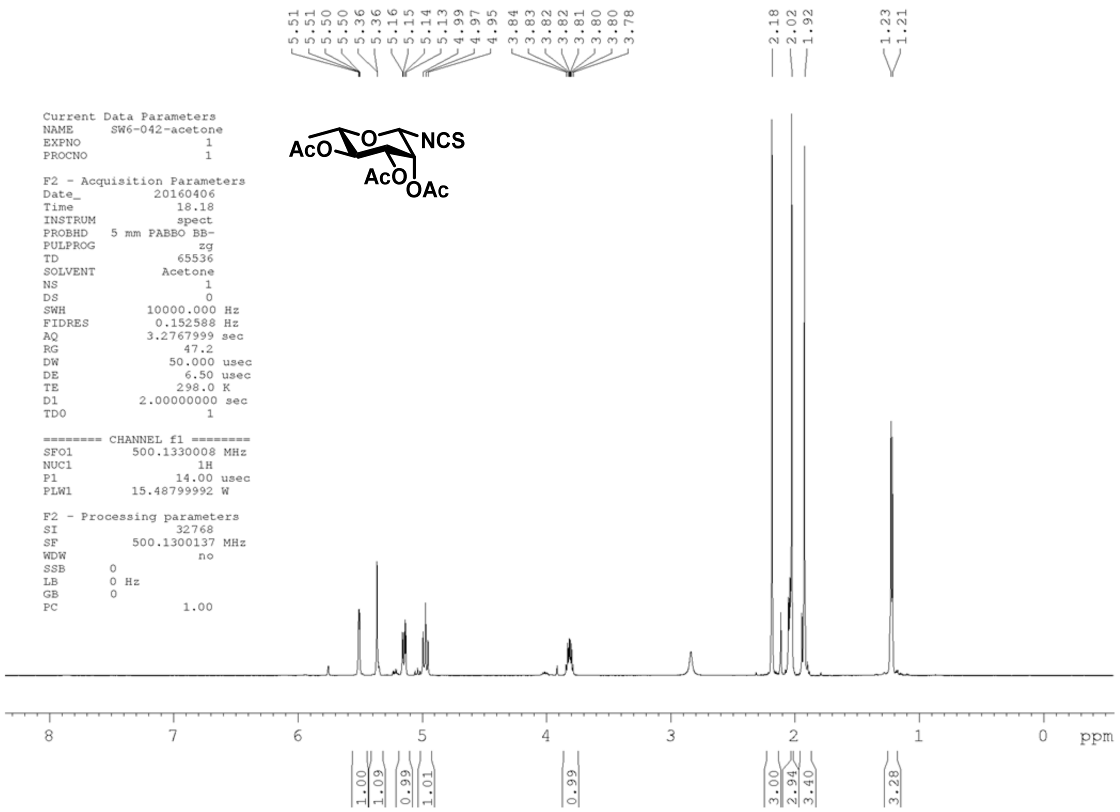


Figure 26: $^{1}$H NMR spectrum for **4**

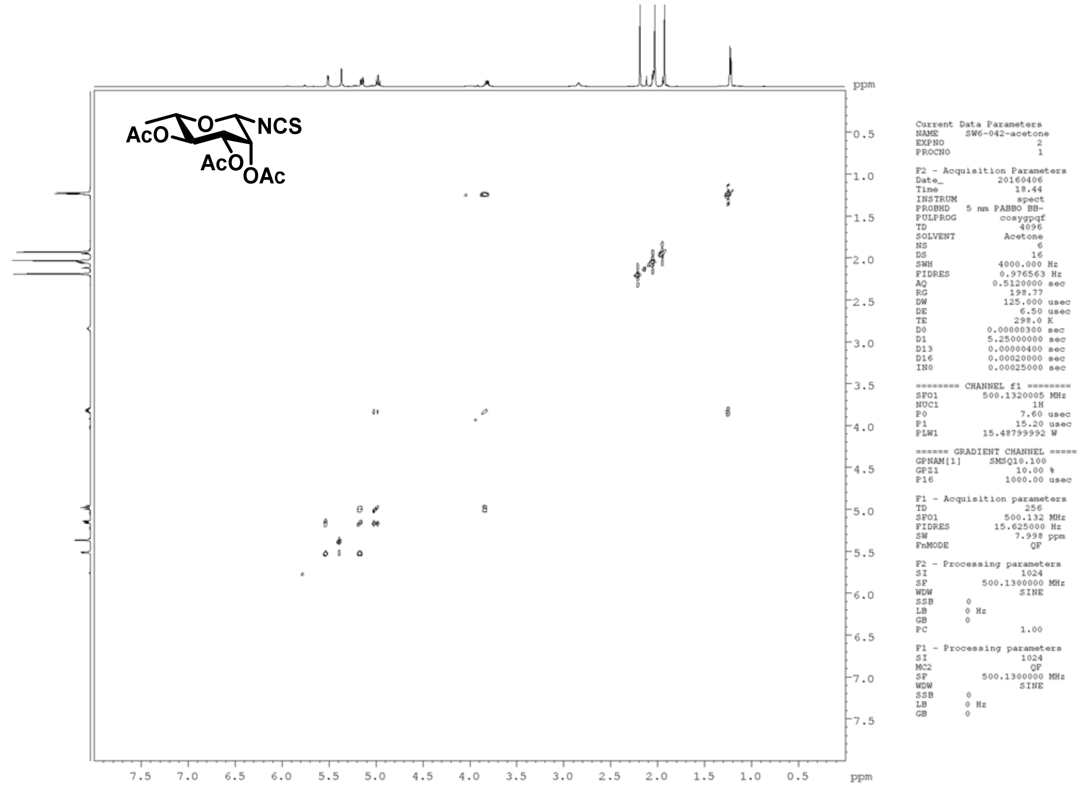


Figure 27: COSY NMR spectrum for **4**

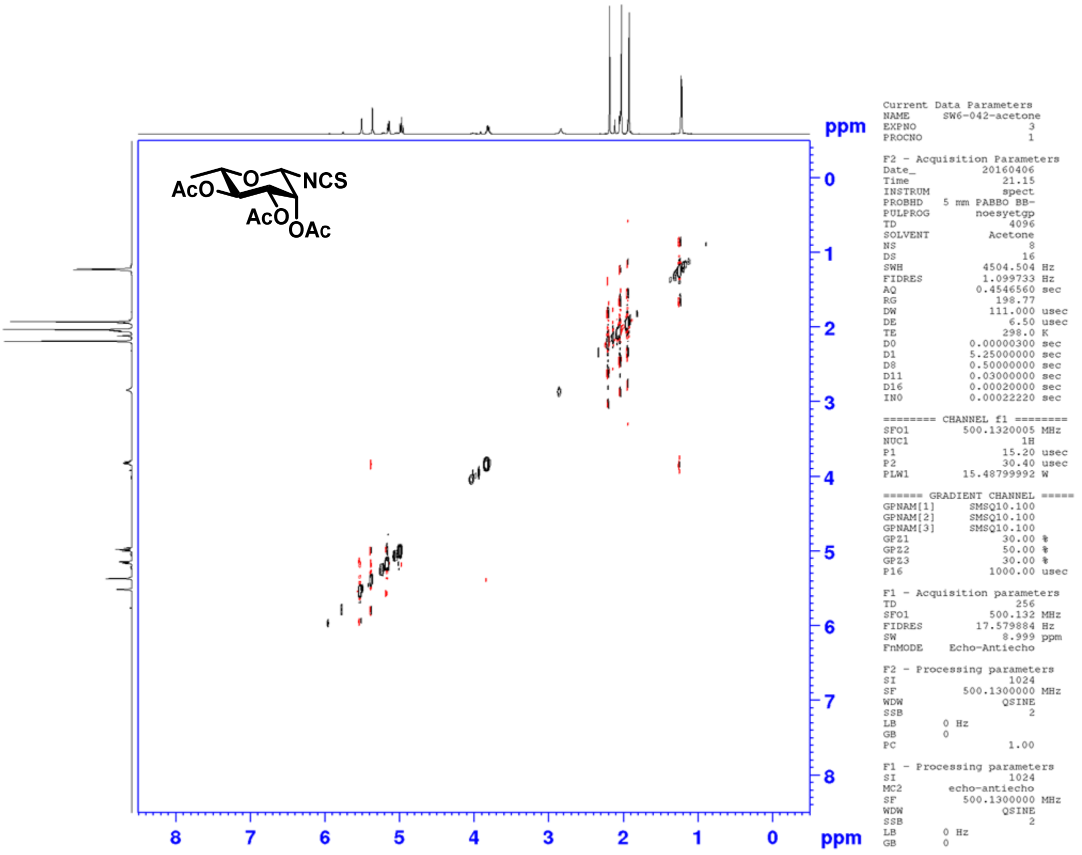


Figure 28: NOESY NMR spectrum for **4**

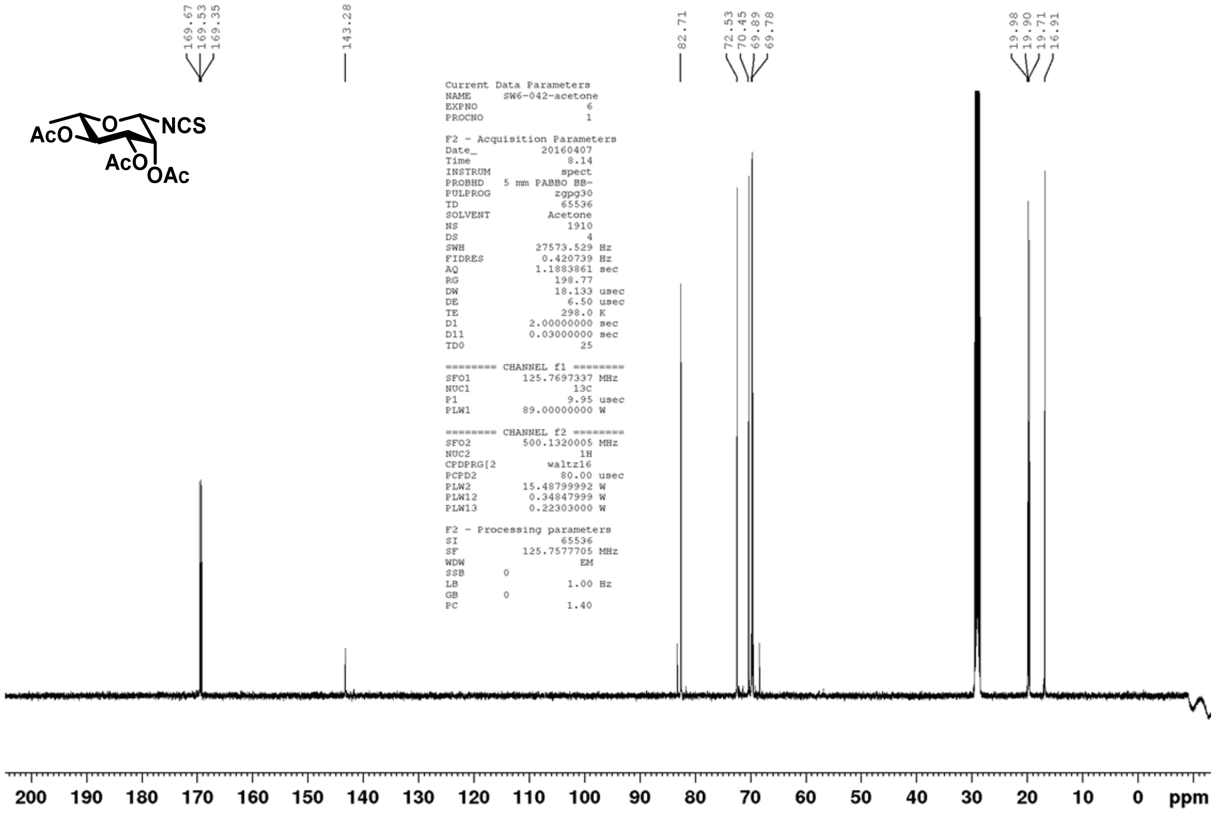


Figure 29: $^{13}C$ NMR spectrum for **4**

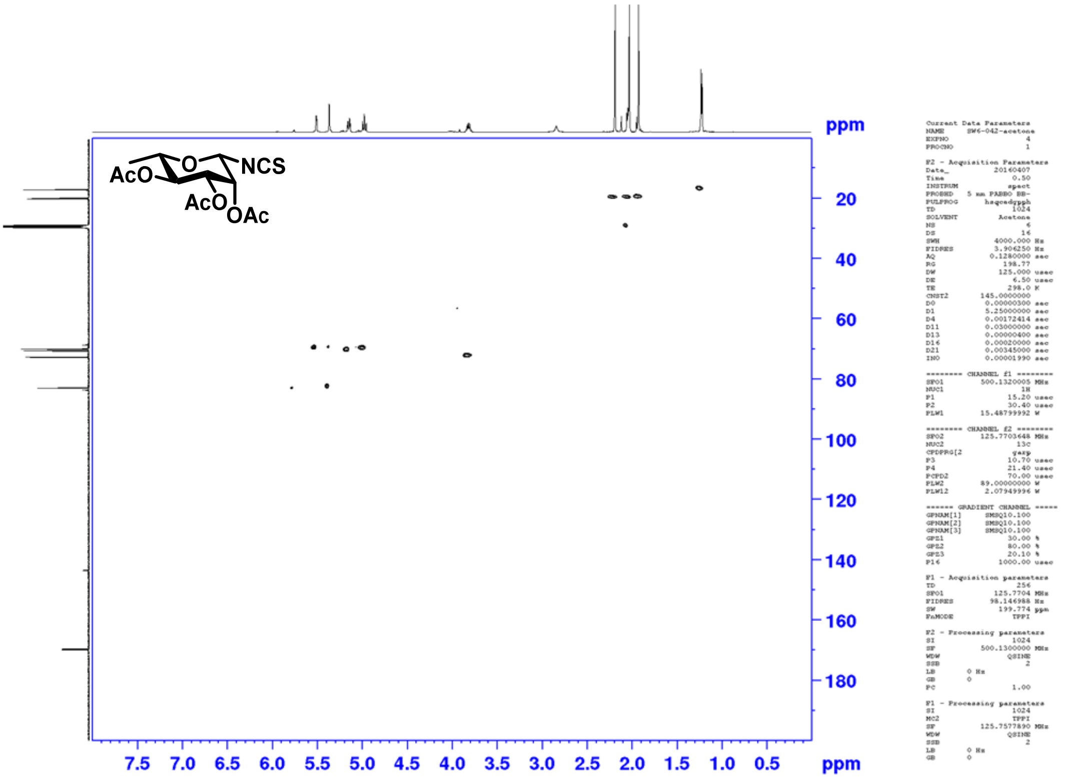


Figure 30: $^{1}$H-$^{13}$C HSQC NMR spectrum for **4**

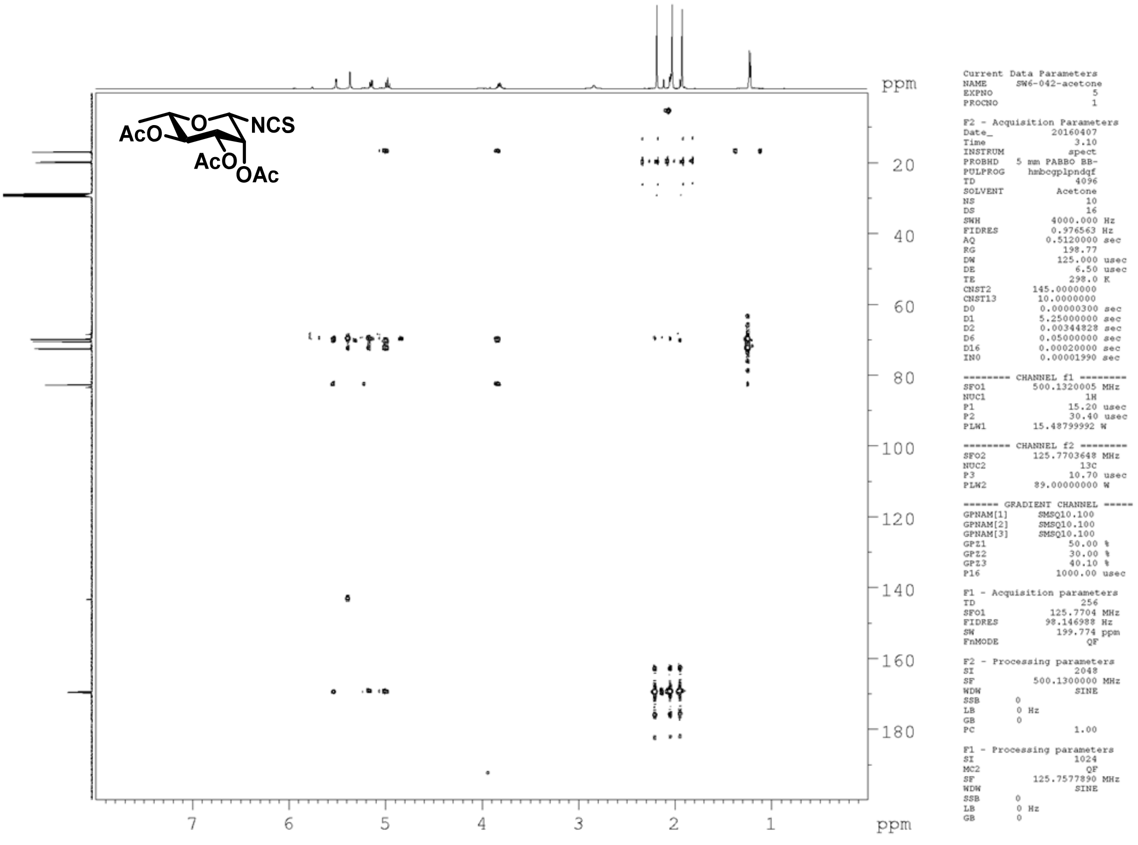


Figure 31: $^{1}$H-$^{13}$C HMBC NMR spectrum for **4**

1-(2,2,4,6,7-Pentamethyldihydrobenzofuran-5-sulfonyl)-3-(2,3,4-tri-*O*-acetyl-6-deoxy-β-L-mannopyranos-1-yl)-2-ethylisothiourea **9**

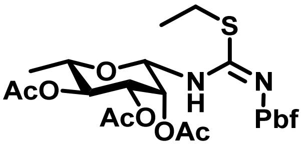


A solution of Pbf-$NH_2$ (540 mg, 2.0 mmol) and KO*t*Bu (224 mg, 2.0 mmol) in anhydrous THF (30 mL) was stirred under argon for 1 h. A solution of rhamnosyl isothiocyanate **4** (620 mg, 1.87 mmol) in THF (20 mL) was added dropwise into the mixture. After 3 h, the solution was neutralized with Amberlite® IR-120 ion exchange resin. After filtration, the filtrate was concentrated under reduced pressure. The crude mixture was dissolved in anhydrous MeCN under argon. Anhydrous $K_2CO_3$ (2.7 g, 20 mmol) was added into the reaction followed by EtI (0.147 mL, 3.0 mmol). The mixture was stirred at 25 °C for 16 h. After the reaction was complete (monitored by LCMS), the mixture was concentrated and purified through flash chromatography with a gradient from 2:1 v/v *n*-hexane:ethyl acetate to 1:2 v/v *n*-hexane:ethyl acetate to yield the desired isothiourea (0.90 g, 1.43 mmol, 77%) as a white foam. $[\alpha]_D$ 13 (c 1.9, acetone). **IR:** 3268 (N-H), 1750 (C=O), 1369 (S=O), 1154 (S=O) $cm^{-1}$. **$^1$H NMR** (400 MHz, d6-Acetone): δ 9.02 (br, $J_{NH\text{-}H1}$ = 9.4 Hz, NH), 5.51 (dd, 1H, $J_{NH\text{-}H1}$ = 9.4 Hz, $J_{1,2}$ = 1.5 Hz, H-1), 5.47 (dd, 1H, $J_{1,2}$ = 1.5 Hz, $J_{2,3}$ = 3.6 Hz, H-2), 5.27 (dd, 1H, $J_{2,3}$ = 3.6 Hz, $J_{3,4}$ = 10.2 Hz, H-3), 4.97 ($t_{apt}$, 1H, $J_{3,4}$ = $J_{4,5}$ = 10.0 Hz, H-4), 3.86 (dq, 1H, $J_{4,5}$ = 9.8 Hz, $J_{5,6}$ = 6.2 Hz, H-5), 3.04 (s, 2H, Ar-C$\underline{H_2}$), 2.97 (m, 2H, S-C$\underline{H_2}$-$CH_3$), 2.55 (s, 3H, Ar-C$\underline{H_3}$), 2.48 (s, 3H, Ar-C$\underline{H_3}$), 2.25 (s, 3H, OAc), 2.09 (s, 3H, Ar-C$\underline{H_3}$), 2.06 (s, 3H, OAc), 1.94 (s, 3H, OAc), 1.48 (s, 6H, C-(C$\underline{H_3}$)$_2$), 1.22 (m, 6H, $J_{5,6}$ = 6.7 Hz, H-6 and S-$CH_2$-C$\underline{H_3}$) ppm. **$^{13}$C NMR** (100 MHz, d6-Acetone): δ 169.8, 169.4, 169.2, 166.3, 159.2, 138.8, 132.8, 131.8, 125.2, 117.2, 86.8, 78.9, 71.4, 70.6, 69.9, 69.5, 42.5, 27.8, 25.4, 19.8, 19.6, 18.5, 17.3, 16.9, 13.7, 11.5 ppm. **HRMS**: (+ESI) m/z, $[M + H]^+$, Calcd: 629.2197, Found: 629.2202

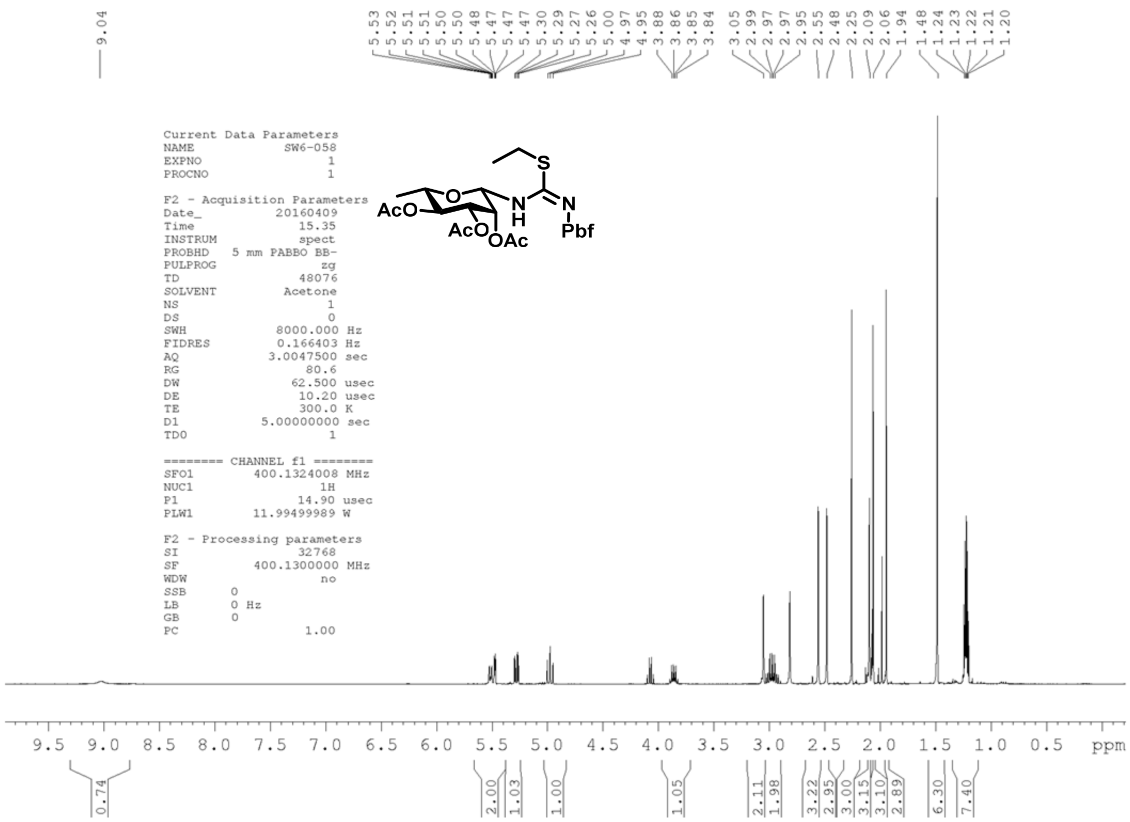


Figure 32: $^{1}$H NMR spectrum for **9**

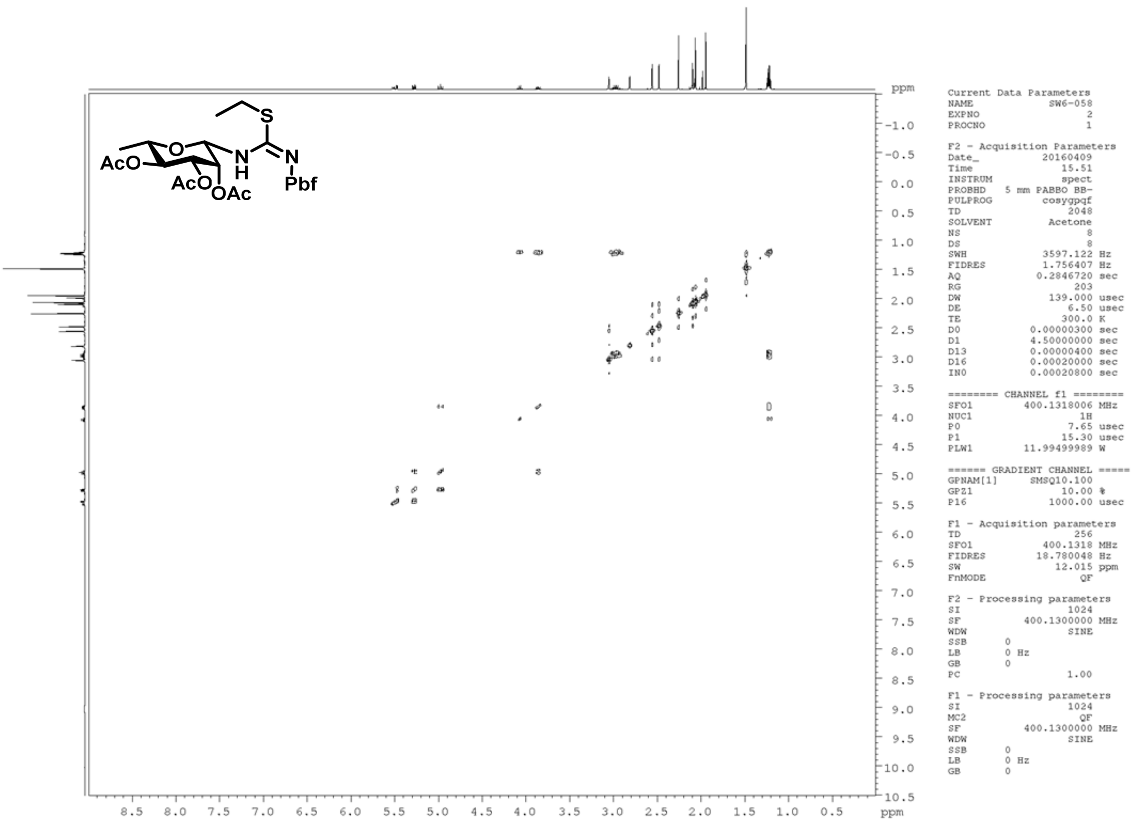


Figure 33: COSY NMR spectrum for **9**

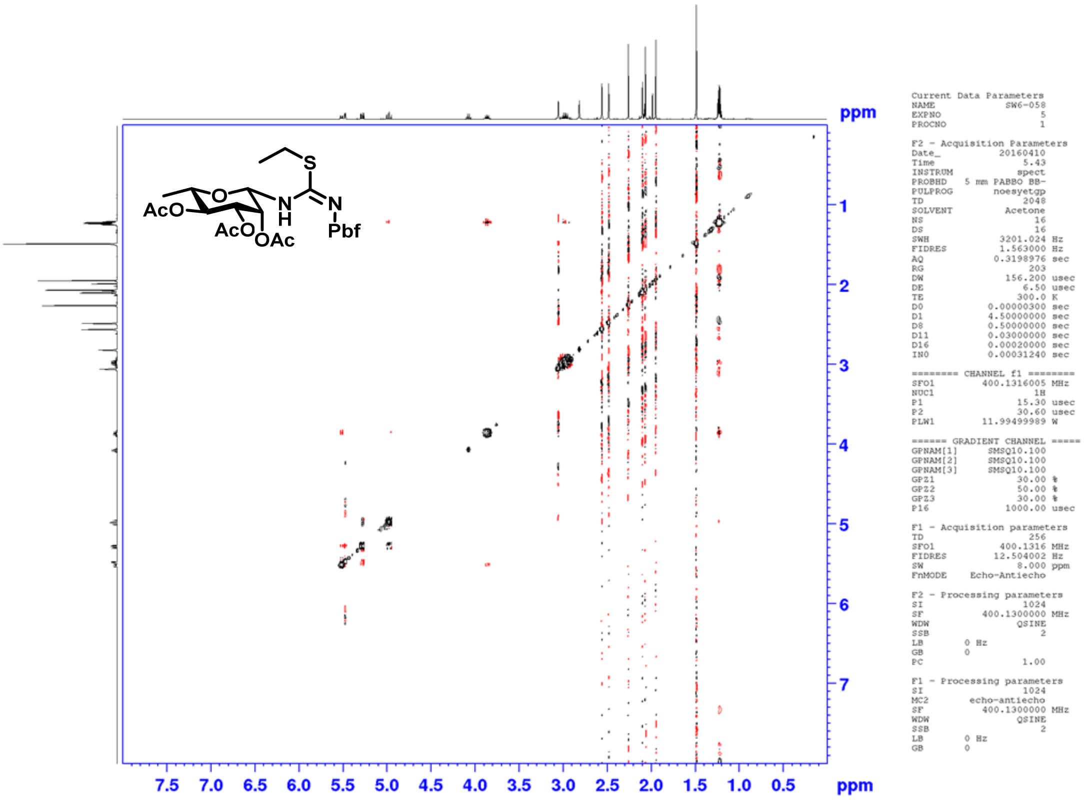


Figure 34: NOESY NMR spectrum for **9**

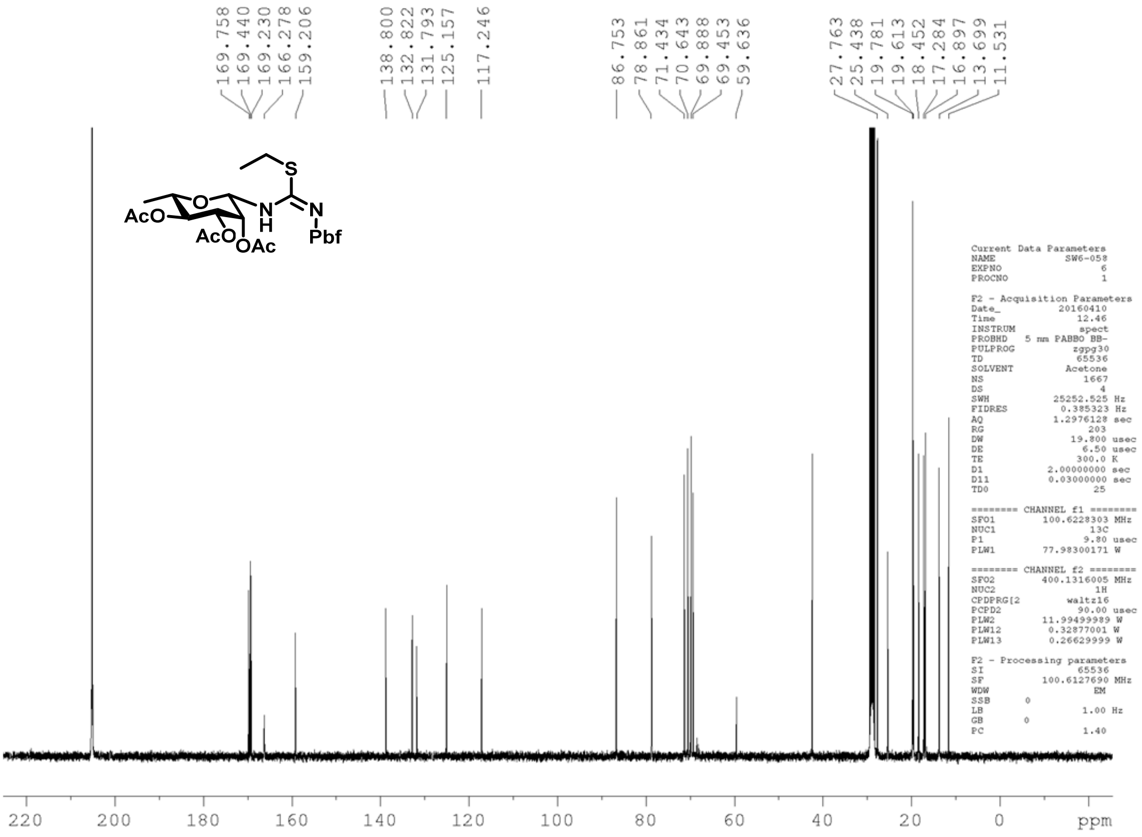


Figure 35: $^{13}C$ NMR spectrum for **9**

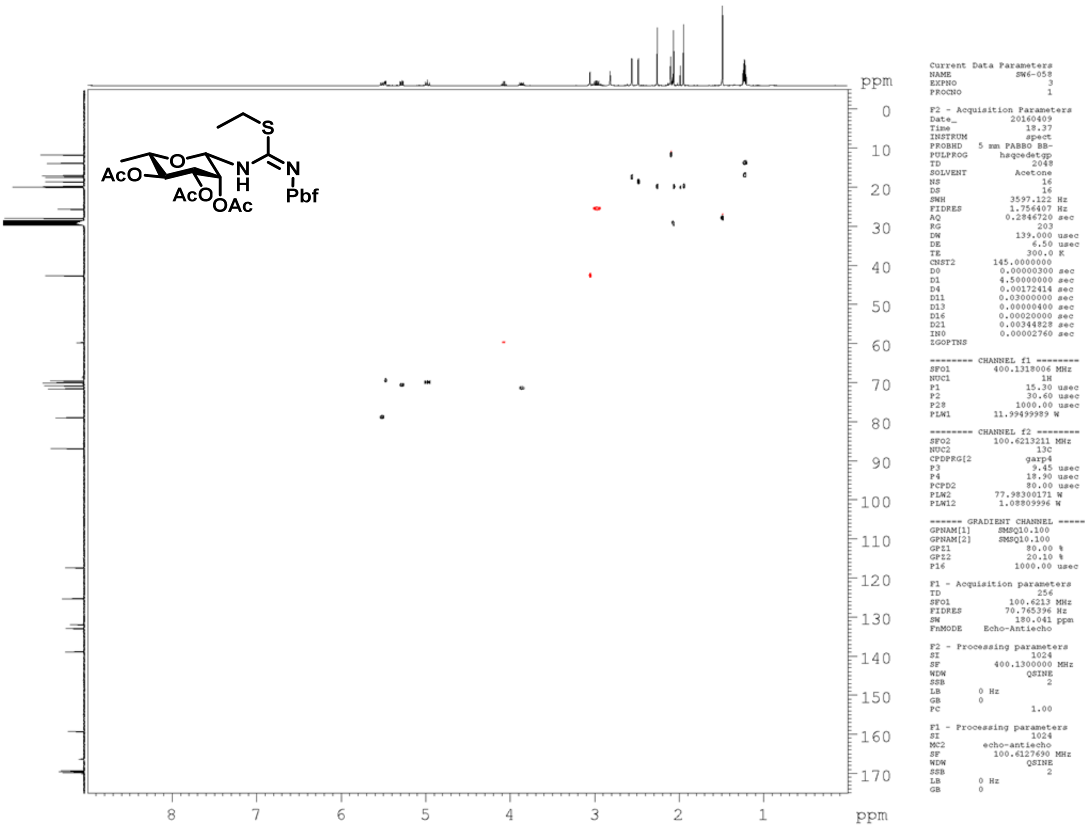

Figure 36: $^{1}H$-$^{13}C$ HSQC NMR spectrum for **9**

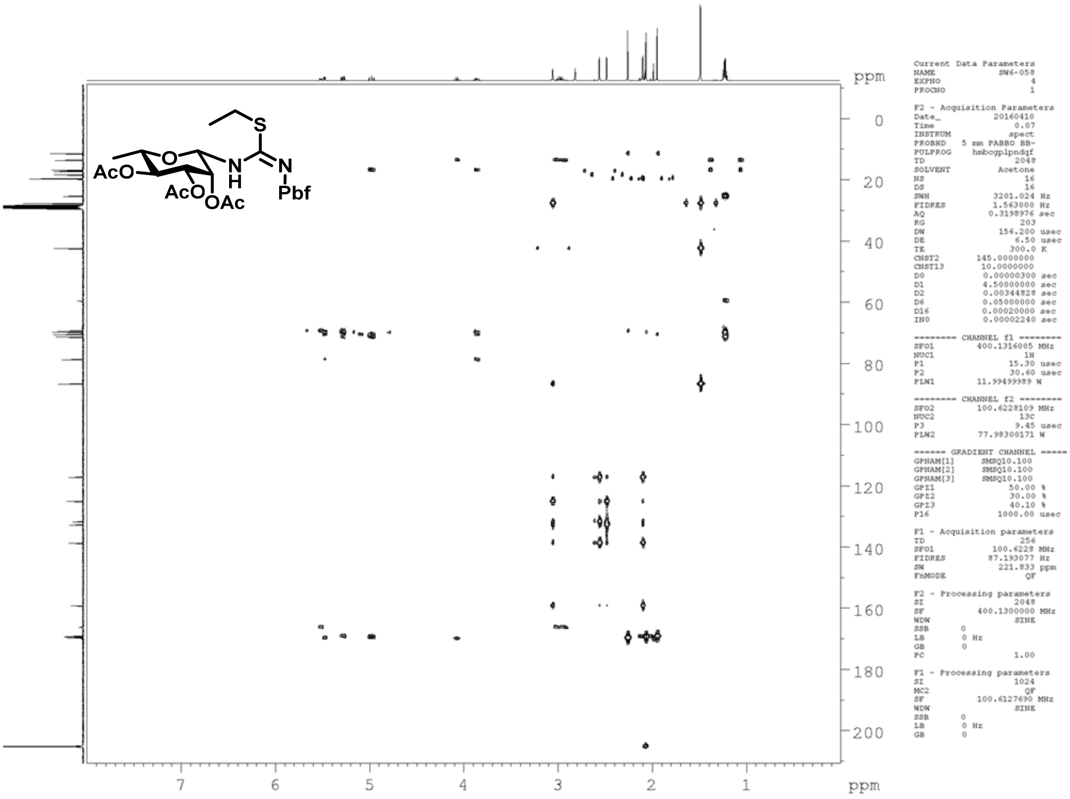

Figure 37: $^{1}H$-$^{13}C$ HMBC NMR spectrum for **9**

*N*α-[(9*H*-fluoren-9-yl)-methoxycarbonyl]-*N*ω-(2,2,4,6,7-pentamethyldihydrobenzofuran-5-sulfonyl)-*N*ω'(2,3,4-tri-*O*-acetyl-6-deoxy-β-L-mannopyranos-1-yl)-L-arginine Allyl Ester **11**

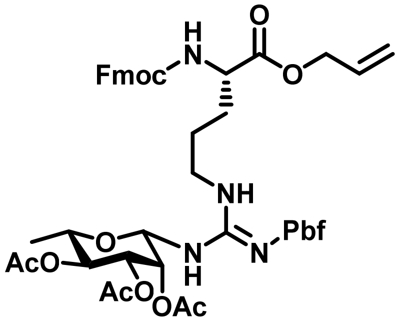


To a solution of rhamnosyl isothiourea **9** (233 mg, 0.37 mmol), Fmoc-Orn-OAll (235 mg, 0.6 mmol) and $NEt_3$ (140 µL, 1.0 mmol) in chloroform (10 mL) was added $HgCl_2$ (160 mg, 0.6 mmol). The mixture was stirred at 25 °C for 2 h. After the reaction was complete (monitored by TLC), the mixture was filtered through a pad of Celite®. The filtrate was concentrated and purified through flash chromatography (2:1 v/v ethyl acetate:*n*-hexane) to yield the desired β-rhamnosylated arginine **11** (340 mg, 0.35 mmol, 94 %) as a foam. $[\alpha]_D$ +27 (c 0.96, acetone). **IR:** 3327 (N-H), 1750 (C=O), 1368 (S=O), 1157 (S=O) $cm^{-1}$. **$^1$H NMR** (400 MHz, d6-Acetone): δ 7.87 (d, 2H, *J* = 7.5 Hz, Ar-H), 7.71 (d, 2H, *J* = 7.3 Hz, Ar-H), 7.43 ($t_{apt}$, 2H, *J* = 7.4 Hz, Ar-H), 7.34 ($t_{apt}$, 2H, *J* = 7.5 Hz, Ar-H), 6.82 (d, 1H, *J* = 7.8 Hz, Fmoc-NH), 5.93 (m, 1H, O-$CH_2$-C<u>H</u>=$CH_2$), 5.41 (d, 1H, $J_{2,3}$ = 3.3 Hz, H-2), 5.40 (br, 1H, H1), 5.34 (dq, 1H, $J_1$ = 17.2 Hz, $J_2$ = 1.6 Hz O-$CH_2$-CH=C$\underline{H_2}$), 5.21 (dq, 1H, $J_1$ = 10.5 Hz, $J_2$ = 1.4 Hz, O-$CH_2$-CH=C$\underline{H_2}$), 5.10 (dd, 1H, $J_{2,3}$ = 3.5 Hz, $J_{3,4}$ = 10.4 Hz, H3), 4.96 ($t_{apt}$, $J_{3,4}$ = $J_{4,5}$ = 10.0 Hz, H-4), 4.60 (d, 2H, *J* = 4.2 Hz, Hz O-C$\underline{H_2}$-CH=$CH_2$), 4.38 (m, 2H, *J* = 7.0 Hz, FmocCH-C$\underline{H_2}$-O-CO), 4.25 (m, 1H, *J* = 7.0 Hz, FmocC<u>H</u>-$CH_2$-O-CO), 4.23 (m, 1H, Arg-Hα,), 3.78 (dq, 1H, $J_{4,5}$ = 10.0 Hz, $J_{5,6}$ = 6.2 Hz, H-5), 3.30 ($q_{apt}$, 2H, *J* = 6.1 Hz, Arg-Hδ), 3.00 (s, 2H, Ar-C$\underline{H_2}$), 2.59 (s, 3H, Ar-C$\underline{H_3}$), 2.49 (s, 3H, Ar-C$\underline{H_3}$), 2.24 (s, 3H, OAc), 2.07 (s, 3H, Ar-C$\underline{H_3}$), 2.03 (s, 3H, OAc), 1.93 (s, 3H, OAc), 1.87 (m, 1H, Arg-Hβ1), 1.74 (m, 1H, Arg-Hβ2), 1.65 (m, 2H, Arg-Hγ), 1.45 (s, 6H, C-(C$\underline{H_3}$)$_2$), 1.17 (d, 3H, $J_{5,6}$ = 6.2 Hz, H-6) ppm. **$^{13}$C NMR** (100 MHz, d6-Acetone): 171.7, 169.9, 169.4, 169.3, 158.3, 156.2, 153.7, 144.2, 144.1, 141.2, 137.9, 134.3, 132.4, 132.0, 127.6, 127.1, 127.1, 125.3, 125.2, 124.6, 120.0, 117.4, 116.7, 86.2, 77.5, 77.2, 71.2, 71.0, 69.9, 69.5, 66.3, 65.1, 54.0, 47.1, 42.7, 40.7, 27.8, 25.6, 19.9, 19.8, 19.6, 18.6, 17.4, 16.9, 11.6 ppm. **HRMS**: (+ESI) m/z, $[M + Na]^+$, Calcd: 983.3719, Found: 987.3714

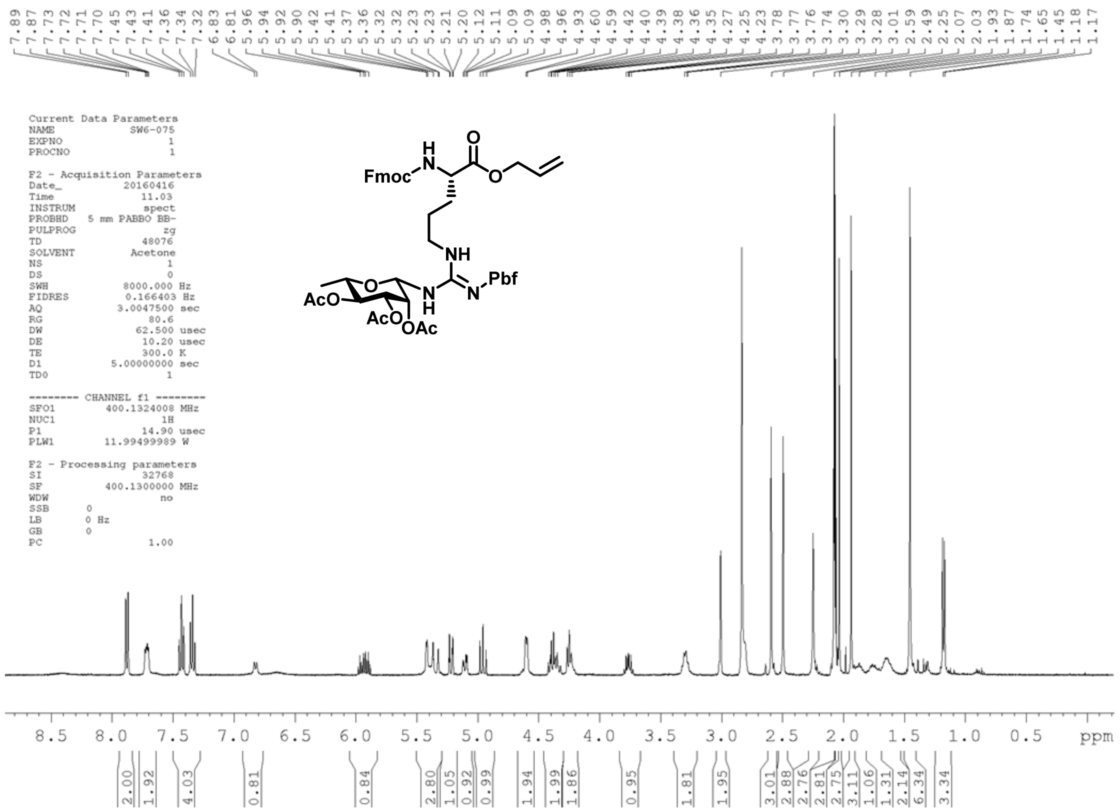


Figure 38: $^{1}$H NMR spectrum for **11**

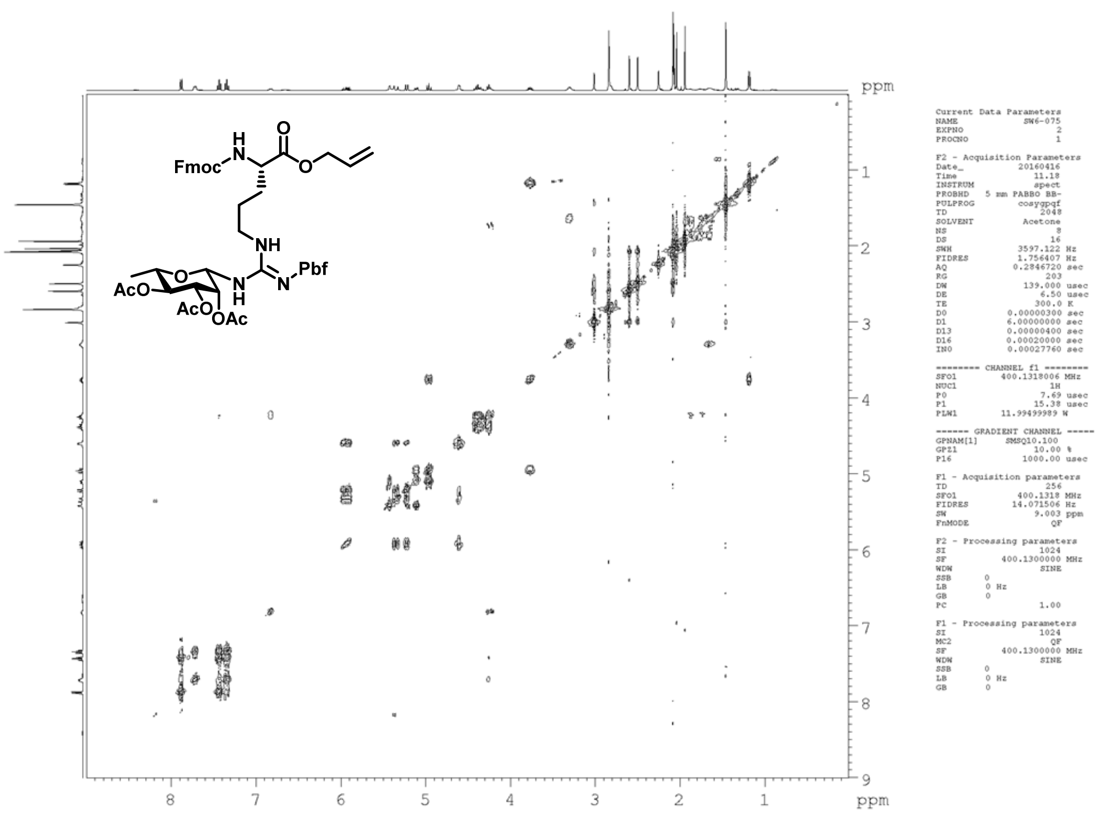


Figure 39: COSY NMR spectrum for **11**

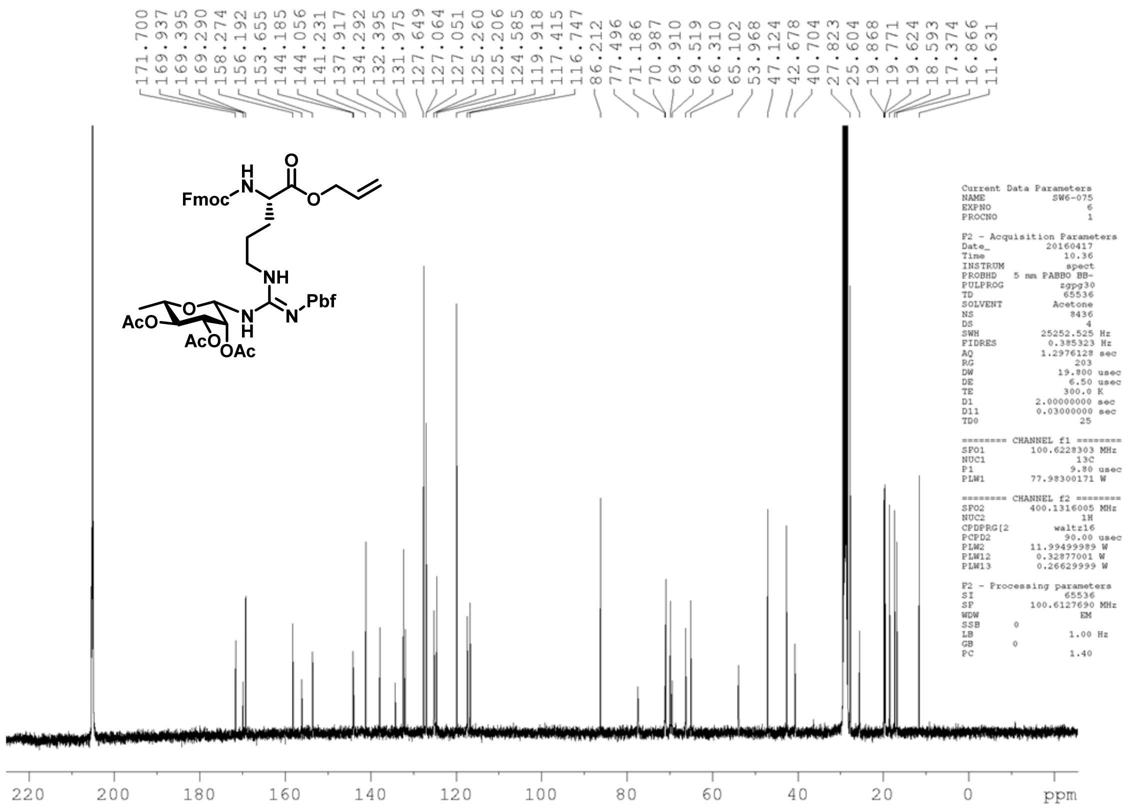


Figure 40: $^{13}$C NMR spectrum for **11**

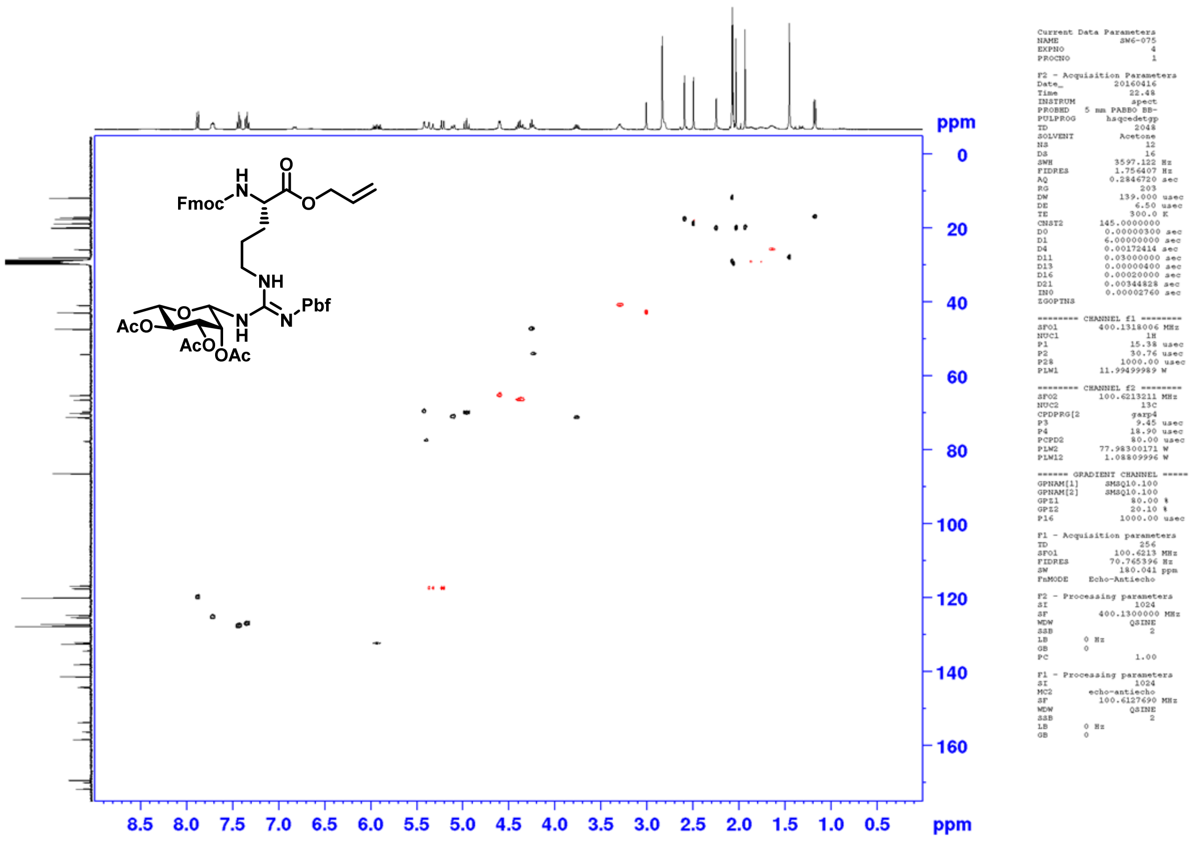


Figure 41: $^{1}$H-$^{13}$C HSQC NMR spectrum for **11**

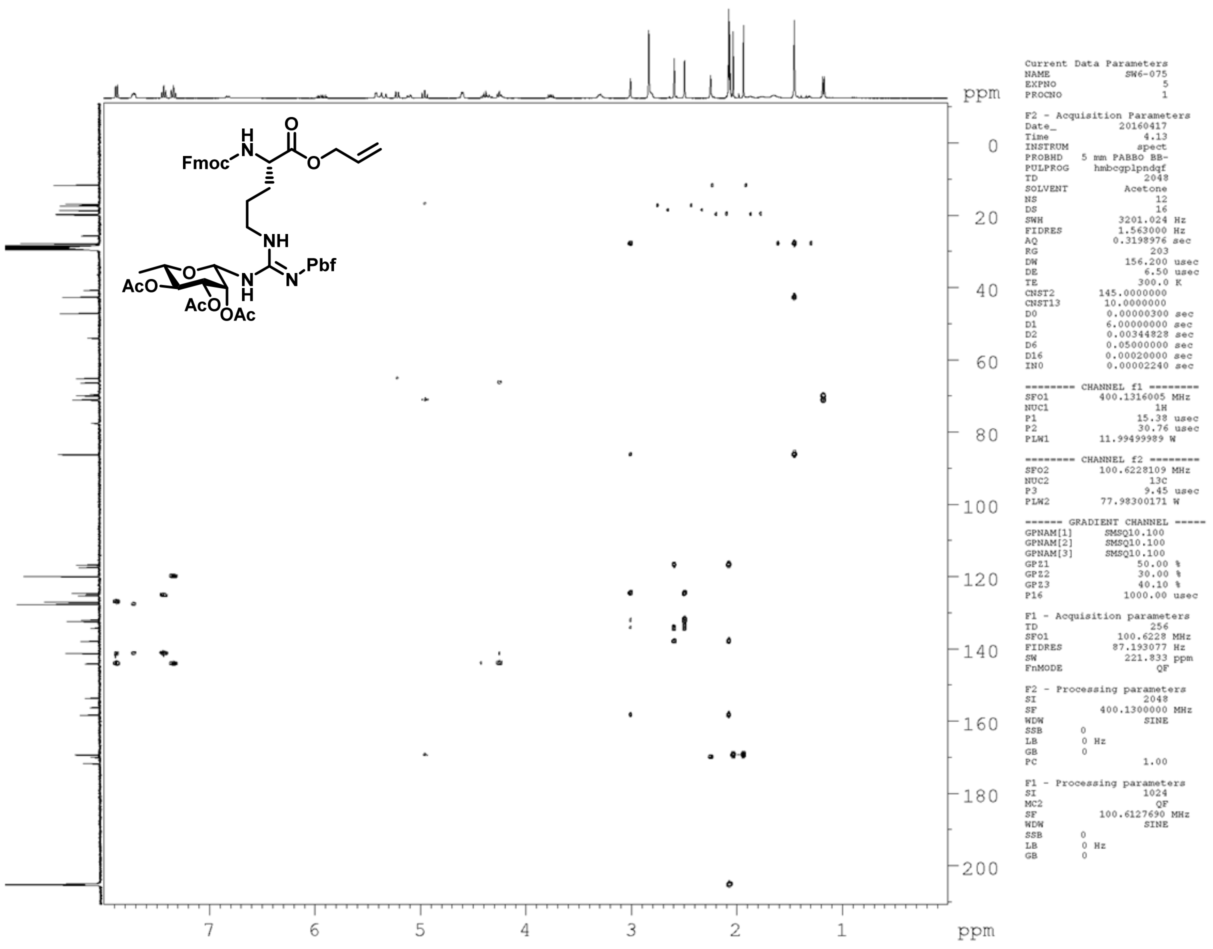


Figure 42: $^1$H-$^{13}$C HMBC NMR spectrum for **11**

*N*α-[(9*H*-fluoren-9-yl)-methoxycarbonyl]-*N*ω-(2,2,4,6,7-pentamethyldihydrobenzofuran-5-sulfonyl)-*N*ω'(2,3,4-tri-*O*-acetyl-6-deoxy-β-L-mannopyranos-1-yl)-L-arginine **2**

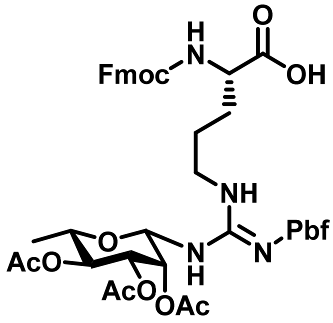


Rhamnosylated arginine **11** (160 mg, 166 μmol) was dissolved in anhydrous THF (15 mL). $Pd(PPh_3)_4$ (100 mg, 86 μmol) was added into the solution followed by phenylsilane (200 μL, 1.6 mmol). The mixture was stirred at 25 °C for 30 min. After the reaction was complete (monitored by TLC), the solution was concentrated under reduced pressure. The mixture was then filtered through a pad of silica with 1% AcOH in ethyl acetate as eluent. The combined filtrate was decolorized over activated charcoal, concentrated and purified with reverse phase HPLC to yield the desired β-rhamnosylated arginine building block **2** (83 mg, 90 μmol, 54%) as a white solid following lyophilisation. $[\alpha]_D$ +15 (c 0.76, acetone). **IR:** 3323 (N-H, O-H), 1751 (C=O), 1370 (S=O), 1160 (S=O) $cm^{-1}$. **$^1$H NMR** (400 MHz, d6-Acetone): δ 7.87 (d, 2H, $J$ = 7.5 Hz, Ar-H), 7.73 (d, 2H, $J$ = 7.5 Hz, Ar-H), 7.42 ($t_{apt}$, 2H, $J$ = 7.4 Hz, Ar-H), 7.33 ($t_{apt}$, 2H, $J$ = 7.5 Hz, Ar-H), 6.70 (d, 1H, $J$ = 7.9 Hz, Fmoc-NH), 5.42 (d, 1H, $J_{2,3}$ = 3.6 Hz, H-2), 5.40 (br, 1H, H1), 5.10 (dd, 1H, $J_{2,3}$ = 3.5 Hz, $J_{3,4}$ = 10.2 Hz, H3), 4.95 ($t_{apt}$, $J_{3,4}$ = $J_{4,5}$ = 10.0 Hz, H-4), 4.37 (m, 2H, $J$ = 7.3 Hz, Fmoc-CH-C$\underline{H_2}$-O-CO), 4.25 (m, 1H, = 7.3 Hz, Fmoc-C$\underline{H}$-$CH_2$-O-CO), 4.23 (m, 1H, Arg-Hα), 3.78 (dq, 1H, $J_{4,5}$ = 9.8 Hz, $J_{5,6}$ = 6.1 Hz, H-5), 3.31 ($q_{apt}$, 2H, $J$ = 6.1 Hz, Arg-Hδ), 3.00 (s, 2H, Ar-C$\underline{H_2}$), 2.60 (s, 3H, Ar-C$\underline{H_3}$), 2.50 (s, 3H, Ar-C$\underline{H_3}$), 2.24 (s, 3H, OAc), 2.07 (s, 3H, Ar-C$\underline{H_3}$), 2.02 (s, 3H, OAc), 1.93 (s, 3H, OAc), 1.91 (m, 1H, Arg-Hβ1), 1.75 (m, 1H, Arg-Hβ2), 1.67 (m, 2H, Arg-Hγ), 1.45 (s, 6H, C-(C$\underline{H_3}$)$_2$), 1.17 (d, 3H, $J_{5,6}$ = 6.2 Hz, H-6) ppm. **$^{13}$C NMR** (100 MHz, d6-Acetone): 172.8, 169.9, 169.4, 169.3, 158.3, 156.2, 153.7, 153.6, 153.6, 144.2, 144.1, 141.2, 137.9, 134.3, 132.0, 127.6, 127.1, 125.3, 125.3, 124.6, 119.9, 116.8, 86.2, 77.4, 71.2, 71.0, 69.9, 69.5, 66.3, 53.6, 53.5, 47.1, 42.7, 40.8, 40.7, 27.8, 25.6, 19.9, 19.8, 19.6, 18.6, 17.4, 16.8, 11.6 ppm. **HRMS:** (+ESI) m/z, $[M + H]^+$, Calcd: 921.3587, Found: 921.3585

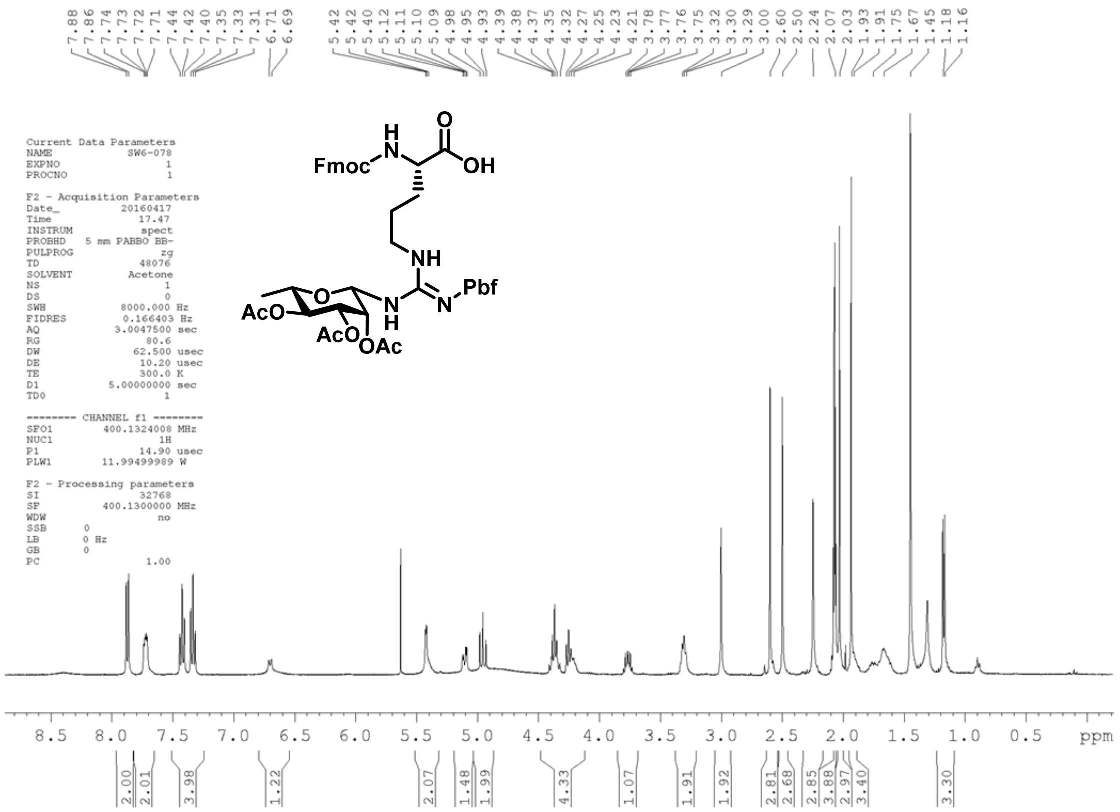


Figure 43: $^{1}$H NMR spectrum for **2**

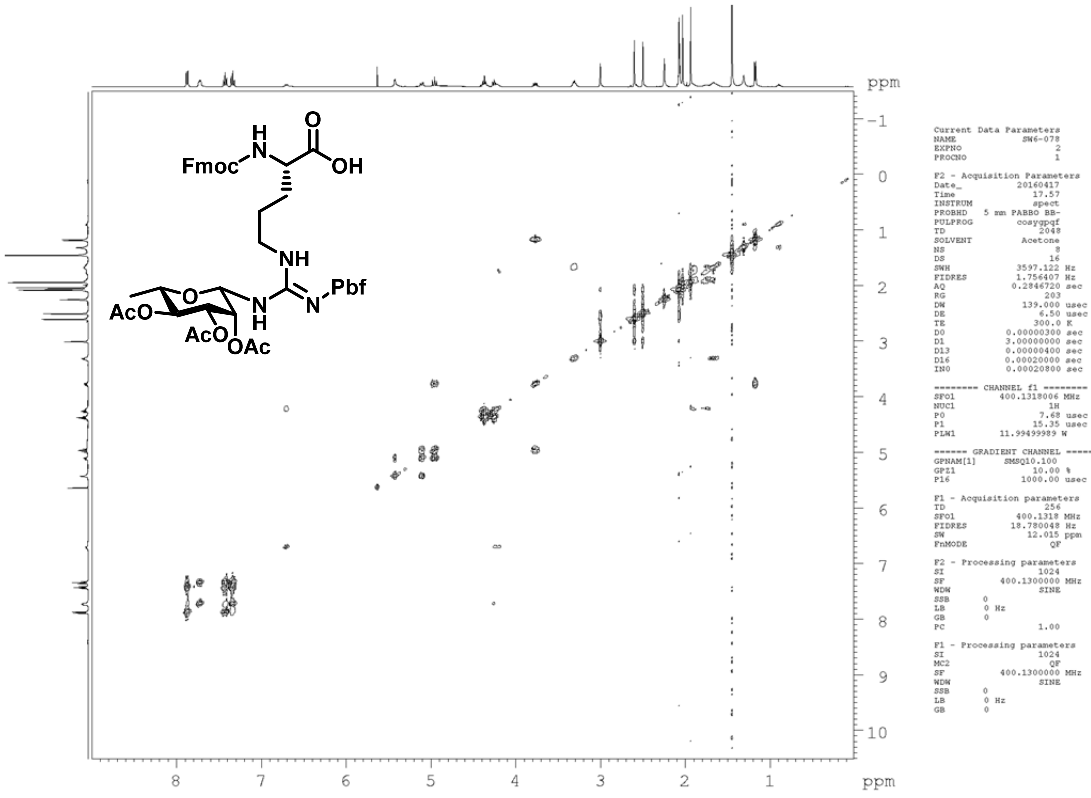


Figure 44: COSY NMR spectrum for **2**

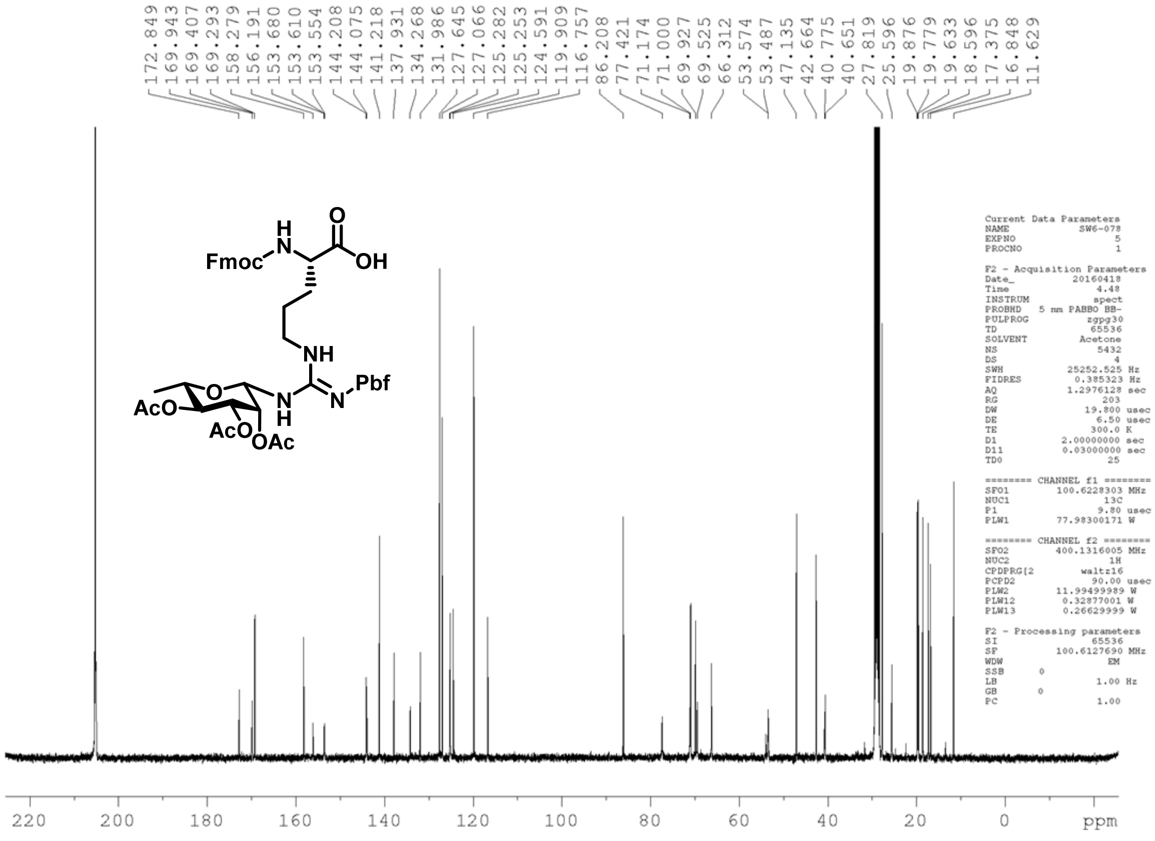


Figure 45: $^{13}$C NMR spectrum for **2**

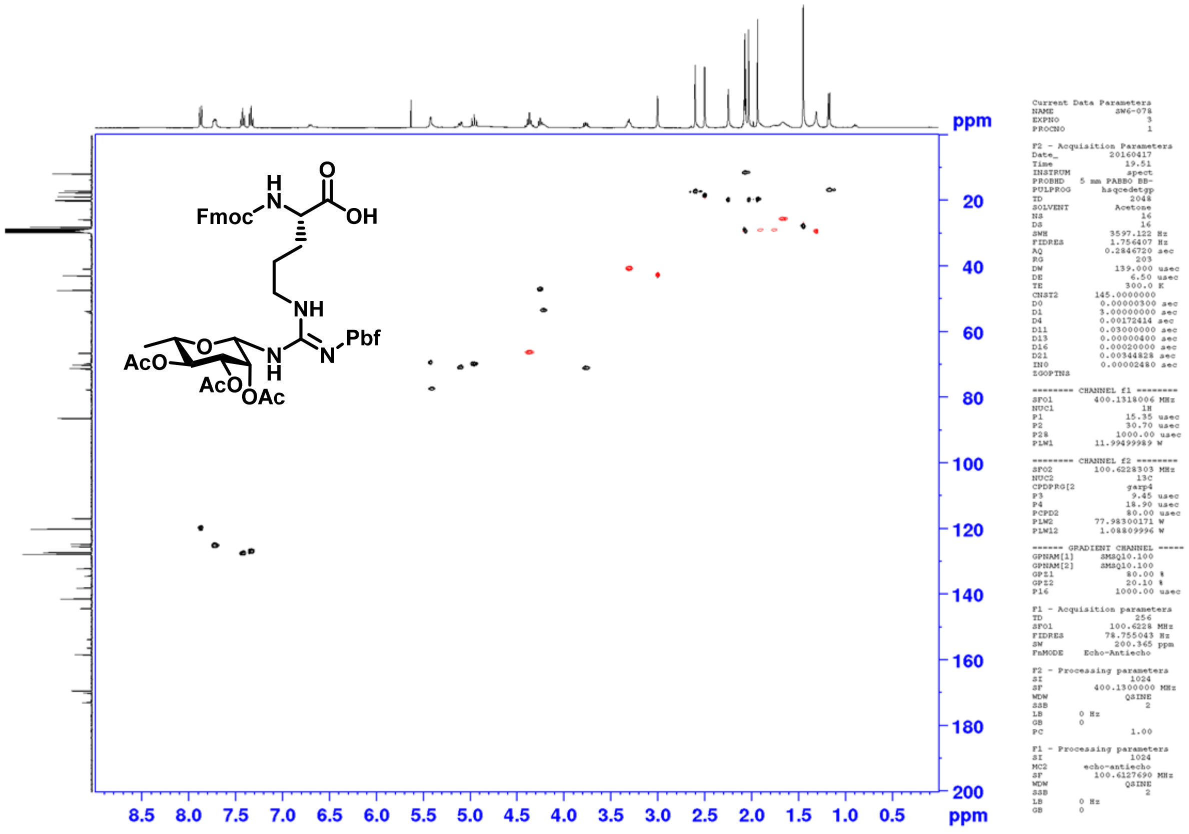


Figure 46: $^{1}$H-$^{13}$C HSQC NMR spectrum for **2**

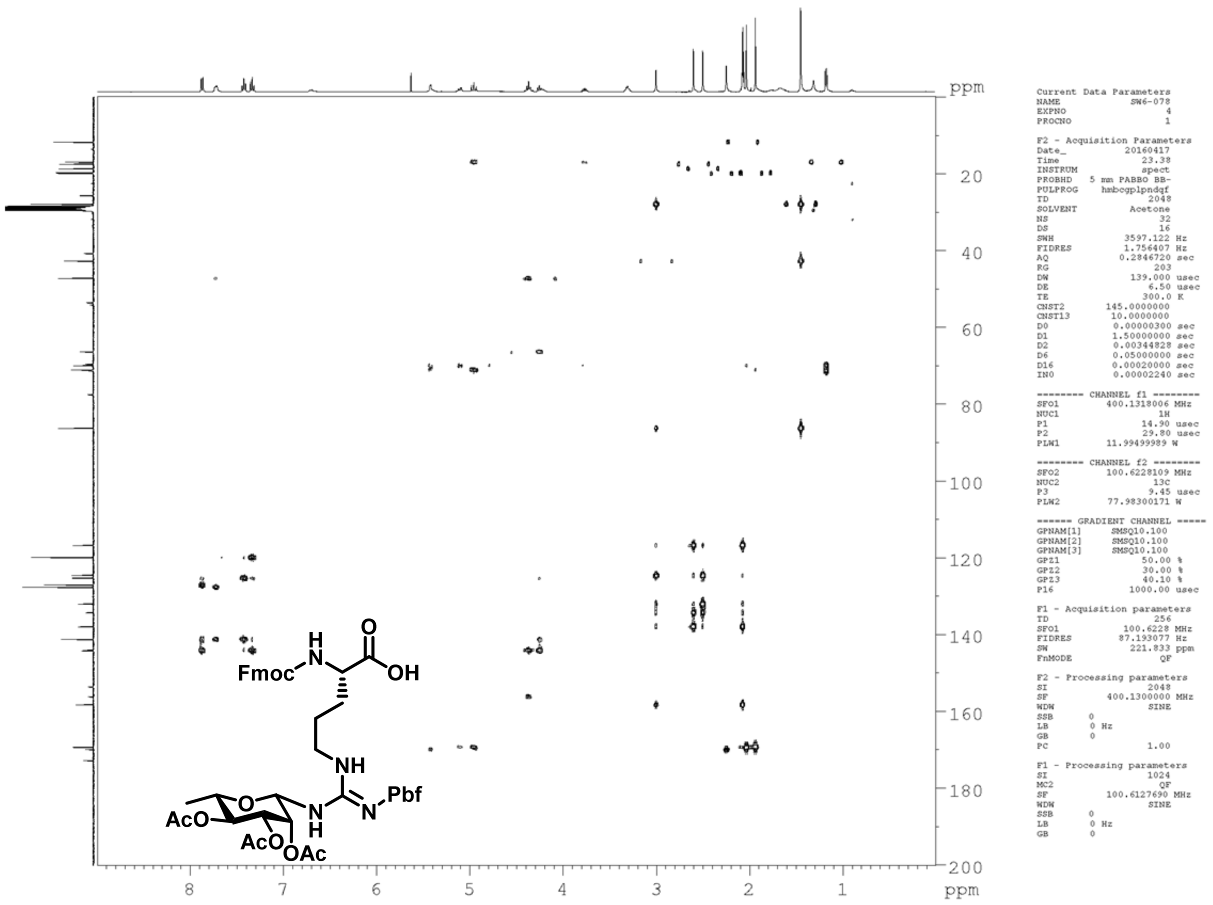


Figure 47: $^{1}H$-$^{13}C$ HMBC NMR spectrum for **2**

*N*α-[(9*H*-fluoren-9-yl)-methoxycarbonyl]-*N*ω-(2,2,4,6,7-pentamethyldihydrobenzofuran-5-sulfonyl)-*N*ω'(2,3,4-tri-*O*-acetyl-6-deoxy- β -L-mannopyranos-1-yl)-L-arginine trifluoroacetate salt **12**

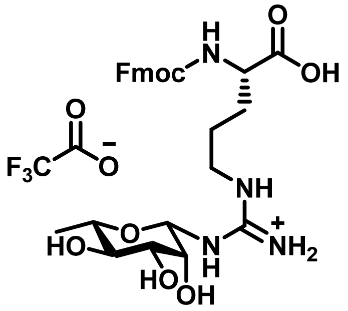


Rhammosylated arginine building block **2** (200 mg, 0.22 mmol) was suspended in TFA/*i*$Pr_3SiH/H_2O$ (18:1:1, 10 mL) at 25 °C with vigorous stirring. After 90 min, the mixture was concentrated and then purified with RP-HPLC. The relevant fractions were recombined and the solvent removed *in vacuo*. The resulting solid was dissolved in dry MeOH (5 mL). NaOMe (0.5 M in MeOH, 0.3 mL, 0.6 mmol) was added into the solution to obtain a final pH of ~8. The mixture was stirred at 25 °C. After 30 min, the reaction was judged to be complete (monitoring by UP-LCMS). The solution was adjusted to pH 4 by careful addition of TFA before concentrating under reduced pressure. The residue was purified through RP-HPLC to afford the deprotected β-rhamnosylated arginine **12** (45 mg, 0.08 mmol, 36%) as white solid following lyophilization. $[\alpha]_D$ +36 (c 0.34, acetone). **IR:** 3324 (N-H, O-H), 1680 (C=O) $cm^{-1}$. $^{1}$**H NMR** (500 MHz, $CD_3OD$): δ 7.80 (d, 2H, *J* = 7.5 Hz, Ar-H), 7.67 ($t_{apt}$, 2H, *J* = 8.5 Hz, Ar-H), 7.40 ($t_{apt}$, 2H, *J* = 7.5 Hz, Ar-H), 7.31 ($t_{apt}$, 2H, J = 7.5 Hz, Ar-H), 4.86 (d, 1H, *J* = 1.2 Hz, H-1), 4.39 (m, 2H, FmocCH-C$\underline{H_2}$-O-CO), 4.23 (m, 1H, FmocC$\underline{H}$-$CH_2$-O-CO), 4.19 (m, 1H, Arg-Hα), 3.87 (dd, 1H, $J_{1,2}$ = 1.3 Hz, $J_{2,3}$ = 3.1 Hz, H-2), 3.45 (dd, 1H, $J_{2,3}$ = 3.3 Hz, $J_{3,4}$ = 9.2 Hz, H-3), 3.39 (dq, 1H, $J_{4,5}$ = 9.3 Hz, $J_{5,6}$ = 5.8 Hz, H-5), 3.34 ($t_{apt}$, $J_{3,4}$ = $J_{4,5}$ = 9.3 Hz, H-4), 3.28 (m, 2H, Arg-Hδ), 1.93 (m, 1H, Arg-Hβ1), 1.73 (m, 1H, Arg-Hβ2), 1.70 (m, 2H, Arg-Hγ), 1.28 (d, 3H, $J_{5,6}$ = 5.8 Hz, H-6) ppm. $^{13}$**C NMR** (100 MHz, $CD_3OD$): 174.0, 160.6 (q, $\underline{C}OCF_3$), 157.4, 156.1, 143.9, 143.7, 141.2, 127.4, 126.8, 126.8, 124.9, 124.8, 119.5, 116.5 (q, CO$\underline{C}F_3$), 78.8 ($J_{C1-H1}$ = 154 Hz), 73.7, 73.3, 71.7, 70.2, 66.6, 53.4, 40.8, 28.5, 24.8, 16.6 ppm. **HRMS**: (+ESI) m/z, $[M + H]^+$, Calcd: 543.2449, Found: 543.2449.

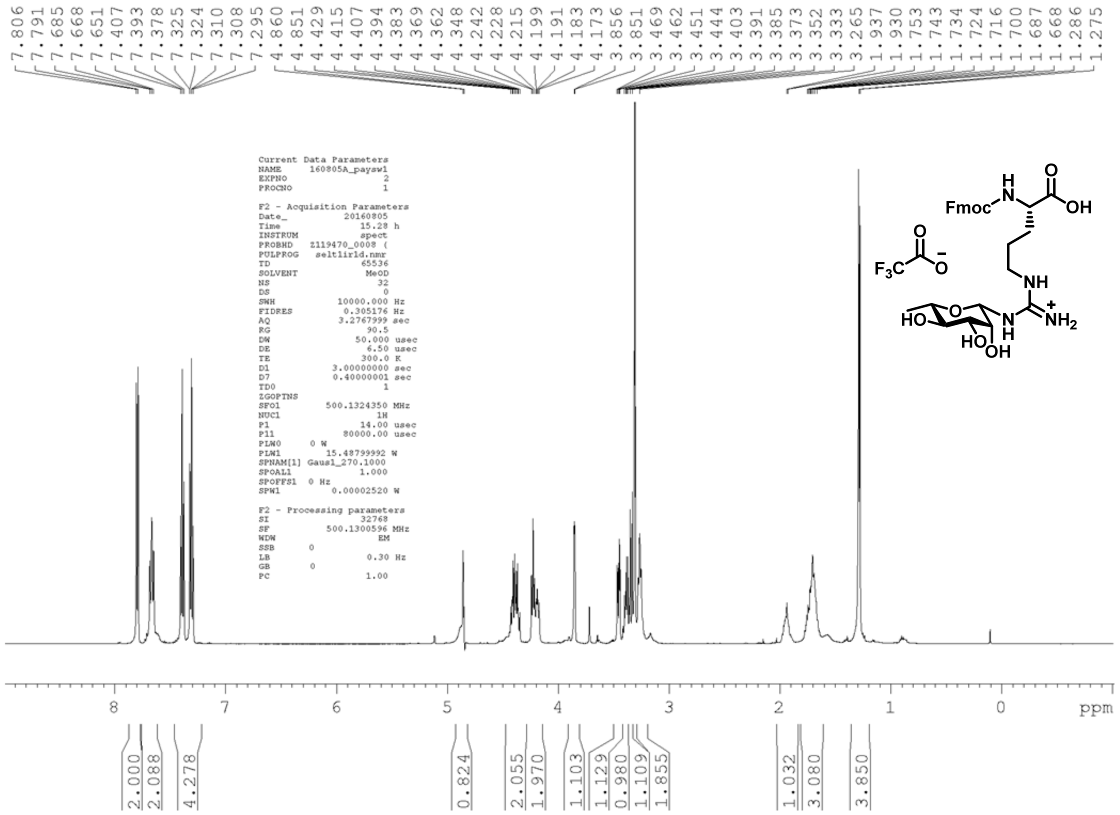

Figure 48: $^{1}$H NMR spectrum for **12**

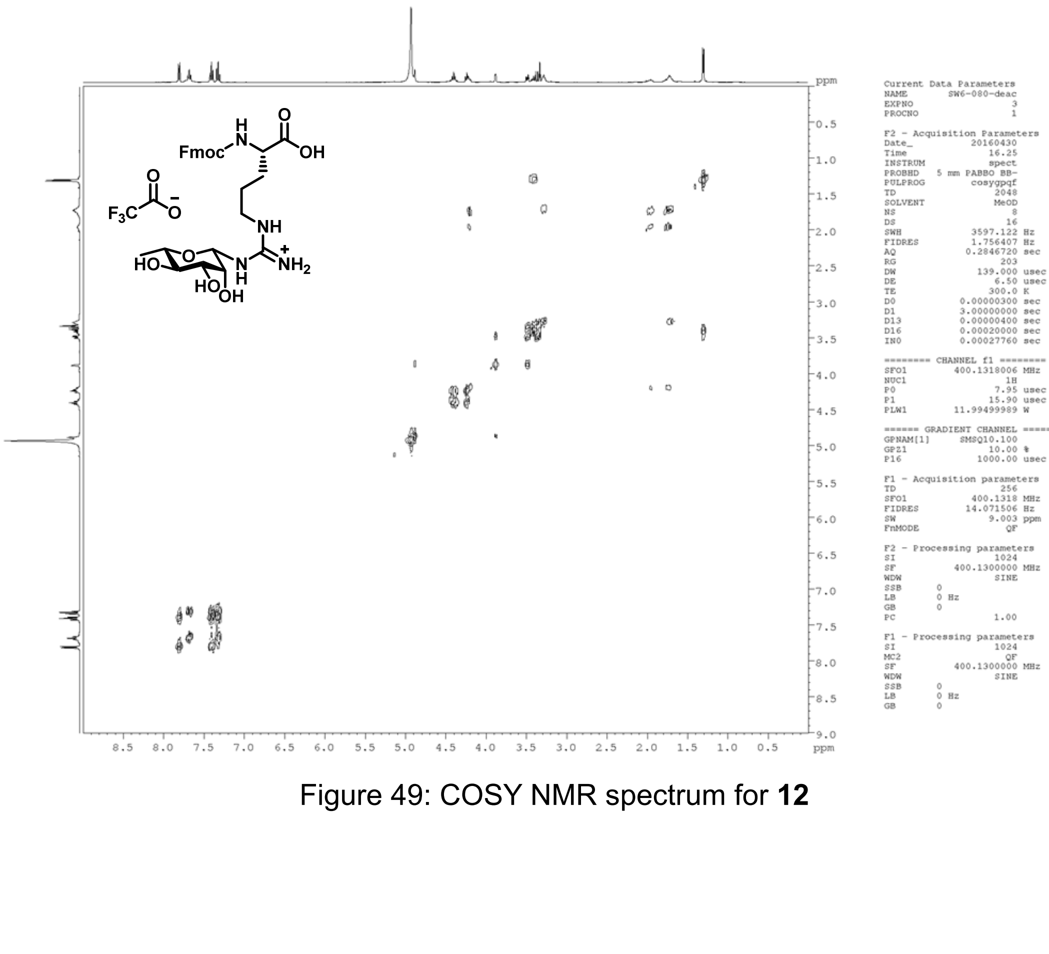

Figure 49: COSY NMR spectrum for **12**

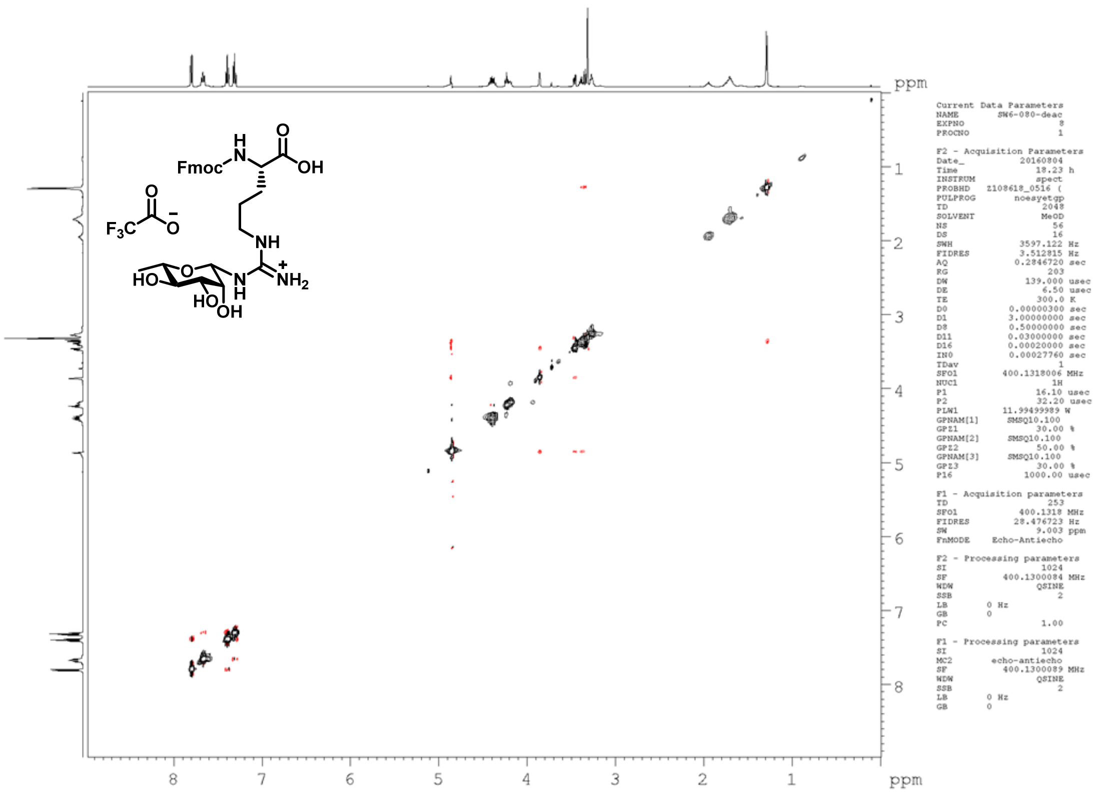

Figure 50: NOESY NMR spectrum for **12**

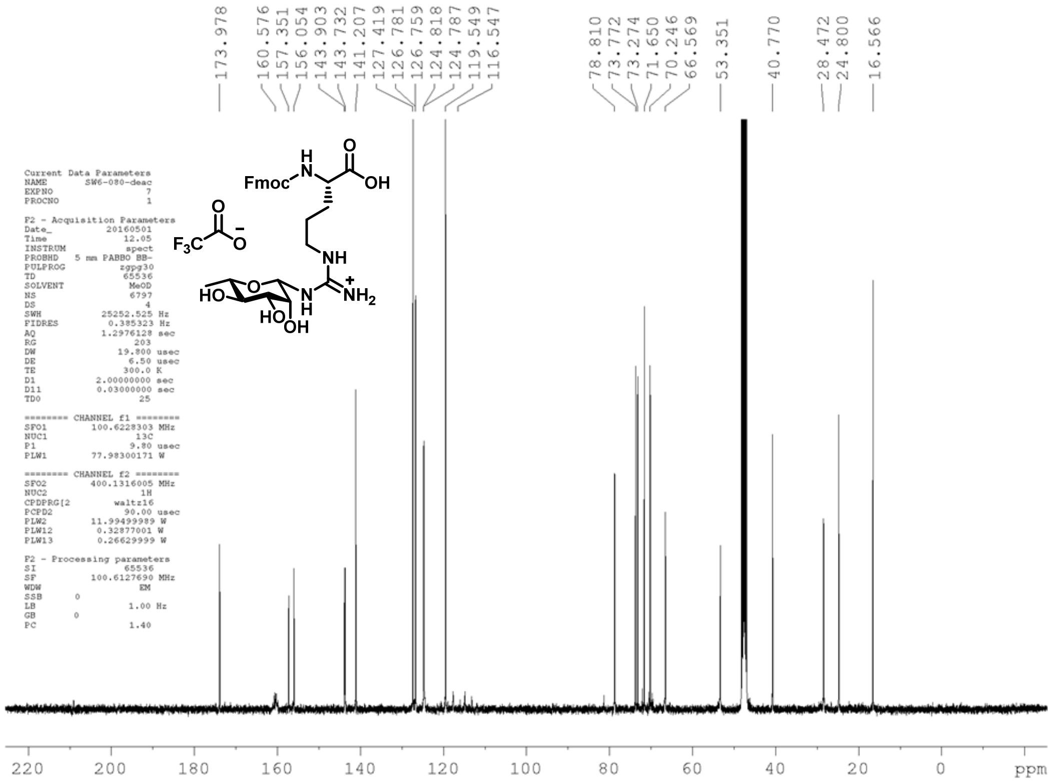


Figure 51: $^{13}$C NMR spectrum for **12**

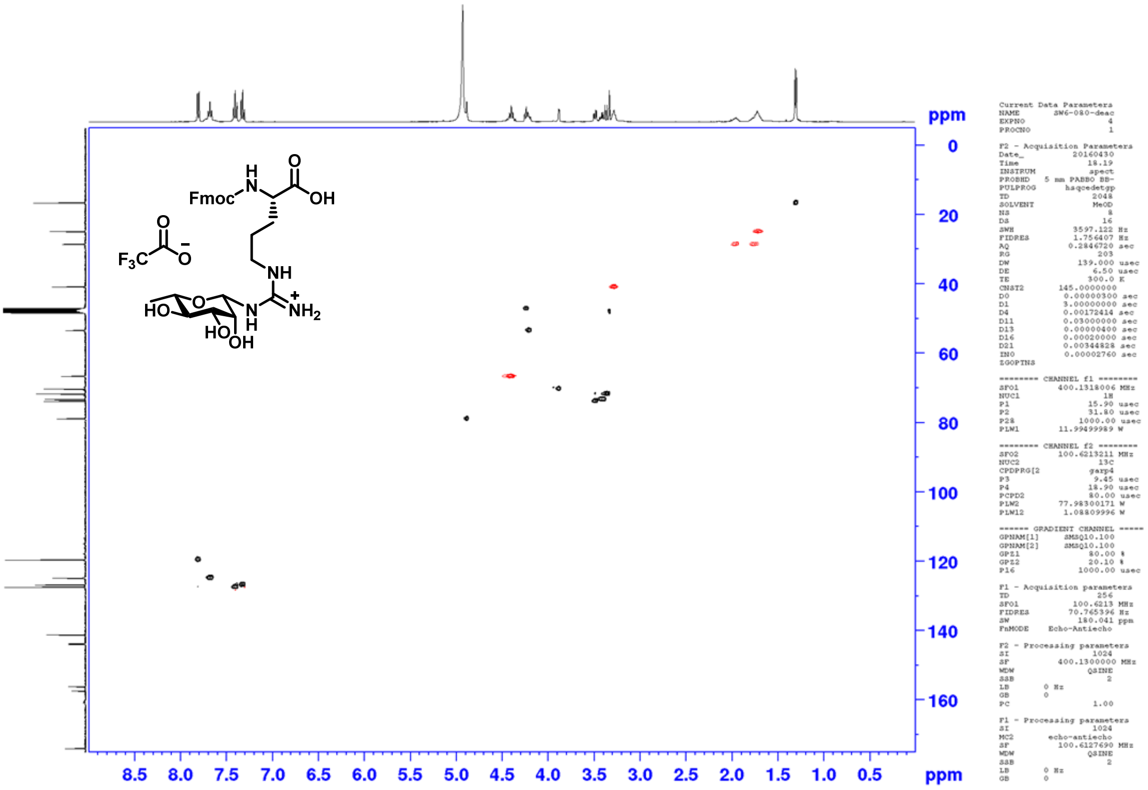


Figure 52: $^{1}H$-$^{13}C$ HSQC NMR spectrum for **12**

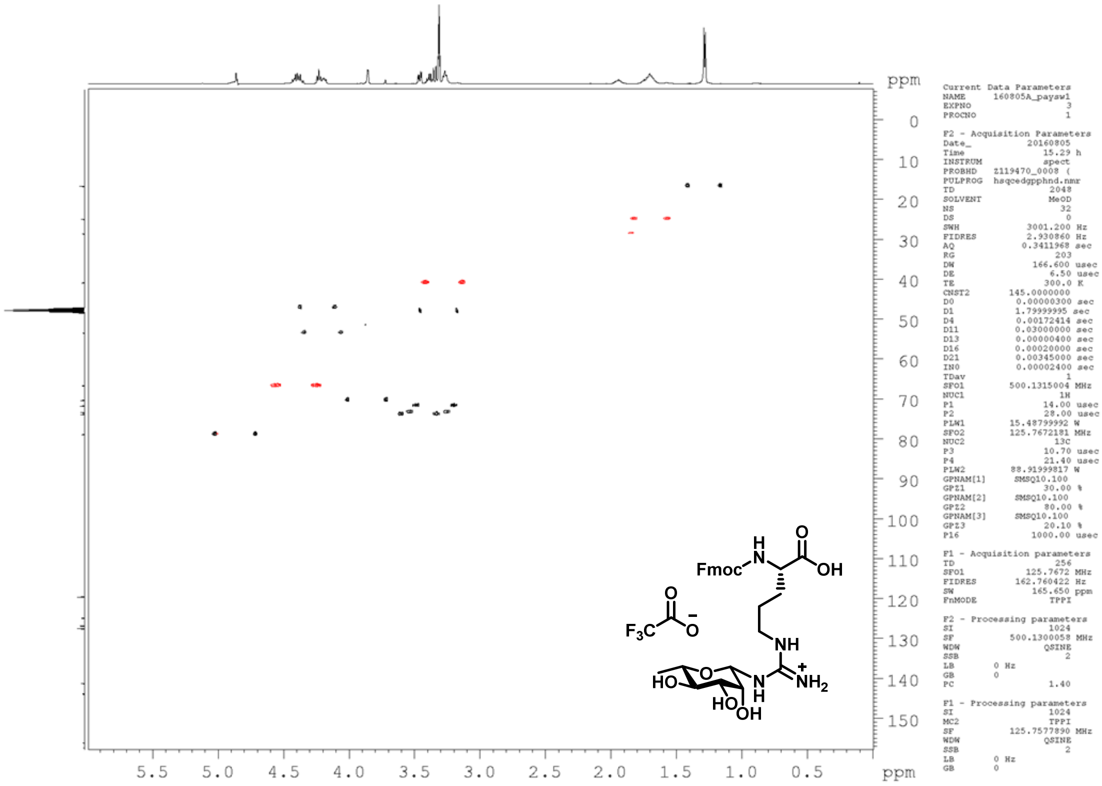


Figure 53: $^{1}H$-$^{13}C$ HSQC without proton decoupling NMR spectrum for **12**

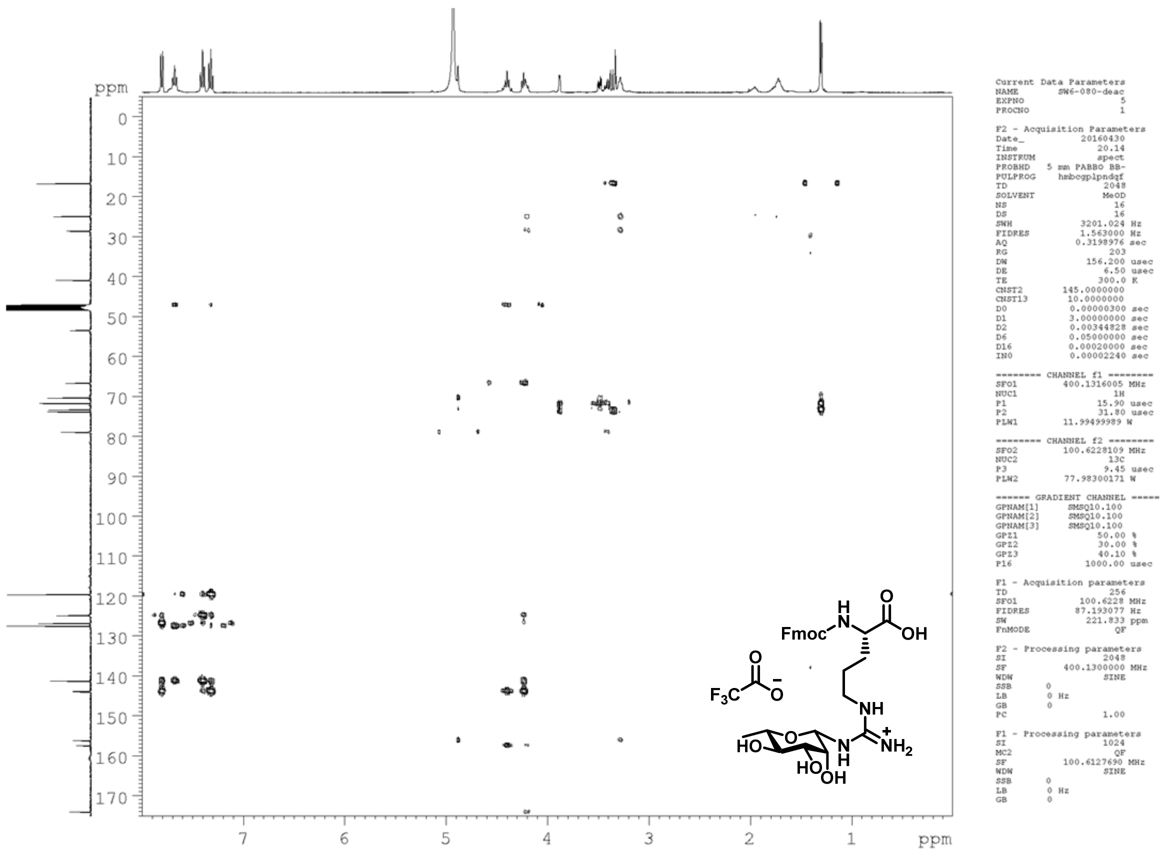


Figure 54: $^{1}H$-$^{13}C$ HMBC NMR spectrum for **12**

## 3. General Procedures for Peptide Synthesis

### 3.1 Preloading of Chemmatrix® Trtyl-OH Resin

Chemmatrix® Trtyl-OH resin (loading capacity 0.3 mmol/g, 0.2 g) was swelled in dichloromethane (6 mL) for 30 min. A solution of 2% $SOCl_2$ in dichloromethane (6 mL) was then added and the resin was shaken for 3 h. After filtration, the resin was washed with dichloromethane (5 x 3 mL) and DMF (5 x 3m). Then a solution of Fmoc-Lys(Boc)-OH (1 mmol) and DIPEA (2 mmol) in dichloromethane (3 mL) was added into the resin and the mixture was agitated for 3 h. The resin was again washed with DMF (5 x 3 mL), dichloromethane (5 x 3 mL) and DMF (5 x 3 mL).

### 3.2 Fmoc Deprotection

The resin was treated with piperidine (20%) in DMF (5 mL, 2 x 5 min, 25 °C) and then washed with DMF (5 x 3 mL), dichloromethane (5 x 3 mL) and DMF (5 x 3 mL).

### 3.3 Coupling of General Amino Acid Building Blocks

A solution of Fmoc-AA-OH (4 equiv.), DIC (4 equiv.), and Oxyma (4 equiv.) in DMF (3 mL) was added to the resin and the mixture was agitated at 50 °C for 25 min. After filtration, the resin was washed with DMF (5 x 3 mL), dichloromethane (5 x 3 mL) and DMF (5 x 3 mL).

### 3.4 Coupling of Rhamnosylated Arginine Building Blocks

A solution of Fmoc-AA-OH (2.1 equiv.), HATU (2.0 equiv.), HOAt (3.0 equiv.) and *sym*-collidine (2.2 equiv.) in DMF (3 mL) was added to the resin and the mixture was agitated at 25 °C for 18 h. After filtration, the resin was washed with DMF (5 x 3 mL), dichloromethane (5 x 3 mL) and DMF (5 x 3 mL).

### 3.5 Capping

A solution of $Ac_2O$/Pyridine (1:9, v/v, 3 mL), was added into the resin. The mixture was agitated at 25 °C for 25 min. After filtration, the resin was washed with DMF (5 x 3 mL), dichloromethane (5 x 3 mL) and DMF (5 x 3 mL).

## 3.6 Deprotection of α-Rhamnosylated Peptide

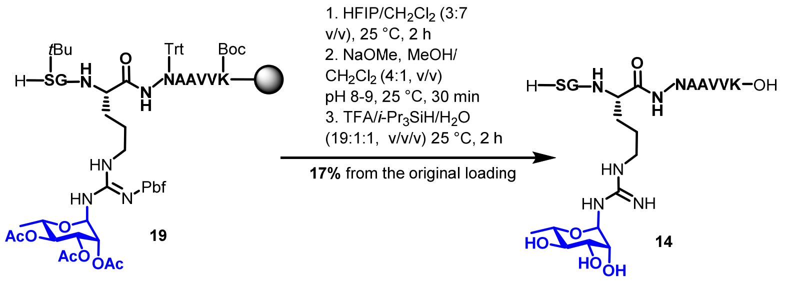


After washing thoroughly with dichloromethane (10 x 5 mL), the resin was suspended in a solution of HFIP in dichloromethane (30 vol%, 5mL, 4 x 30 min). After filtration, the resin was washed with dichloromethane and the combined filtrate was concentrated under reduced pressure. The residue was re-dissolved in MeOH/dichloromethane (4:1, v/v, 10 mL). NaOMe (0.5 M in MeOH) was carefully added into the solution to give a final pH 8. The resulting solution was stirred at 25 °C with continuous UPLC-MS monitoring. After completion, the reaction was quenched with formic acid (100 μL). The solvent was removed by blowing with a gentle flow of nitrogen gas. The residue was then suspended in TFA/*i*-$Pr_3SiH/H_2O$ (18:1:1,v/v/v) for 2 h. The resulting mixture was concentrated and purified with RP-HPLC.

Note: either higher pH or higher dichloromethane to methanol ratio will accelerate anomerization during Zemplén deacetylation.

## 3.7 Deprotection of β-Rhamnosylated Peptide

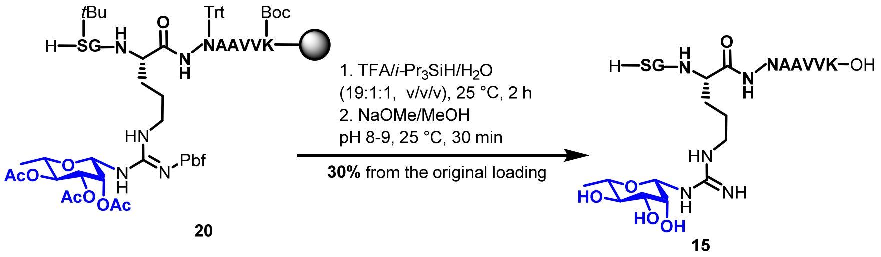


After washing thoroughly with dichloromethane (10 x 5 mL), the resin was suspended in TFA/*i*$Pr_3SiH/H_2O$ (18:1:1,v/v/v) for 2 h. After filtration, the filtrate was concentrated by blowing with a gentle flow of nitrogen gas. The residue was then re-dissolved in MeOH (5 mL). NaOMe (0.5 M in MeOH, 1 mL) was added into the solution to give a final pH of 8-9. The resulting solution was stirred at 25 °C for 2 h. After completion (monitoring with UPLC-MS), the reaction was quenched with formic acid (100 μL). The resulting mixture was concentrated and purified with RP-HPLC.

# 4. Synthesis and Characterization of Rhamnosylated Peptides

## 4.1 α-Rhamnosylated Peptide **14**

The synthesis of α-rhamnosylated peptide **14** was conducted on a 25 μmol scale based on Fmoc loading assay after loading the first amino acid. After HPLC purification and lyophilization, the peptide was obtained as a white powder (5.8 mg, 4.25 μmol, 17%, **14**α:**15**β 9:1). **$^1$H NMR** (500 MHz, $D_2O$): 5.08 (d, $J_{1,2}$ = 1.7 Hz, 1H, $J_{H1\text{-}C1}$ = 167 Hz, Rha-H1), 4.60 (dd, 1H, Asn-Hα), 4.30 – 3.8 (m, 12H, Arg-Hα, Ser-Hα, 2 x Ala-Hα, Lys-Hα, Gly-Hα$_1$, Gly-Hα$_2$, Rha-H2, 2 x Val-Hα, Ser-Hβ$_1$, Ser-Hβ$_2$), 3.66 (dd, 1H, $J_{2,3}$ = 3.6 Hz, $J_{3,4}$ = 9.7 Hz, Rha-H3), 3.55 (dq, 1H, $J_{4,5}$ = 9.4 Hz, $J_{5,6}$ = 6.4 Hz, Rha-H5), 3.40 ($t_{apt}$, 1H, $J_{3,4}$ = $J_{4,5}$ = 9.5 Hz, Rha-H4), 3.20 (t, 2H, Arg-Hδ), 2.90 (t, 2H, Lys-Hε), 2.74 (dd, 1H, Asn-Hβ$_1$), 2.66 (dd, 1H, Asn-Hβ$_2$), 2.0 – 1.3 (m, 12H, 2 x Val-Hβ, 2 x Arg-Hβ, 2 x Lys-Hβ, 2 x Arg-Hγ, 2 x Lys-Hγ, 2 x Lys-Hδ), 1.29 (d, 3H, Ala-C$\underline{H}_3$), 1.28 (d, 3H, Ala-C$\underline{H}_3$), 1.17 (d, 3H, $J_{5,6}$ = 6.2 Hz, Rha-H6), 0.88 – 0.80 (m, 12H, 4 x Val-C$\underline{H}_3$) ppm. **HRMS**: (+ESI) m/z, $[M + H]^+$, Calcd: 1047.5793, Found: 1047.5792.

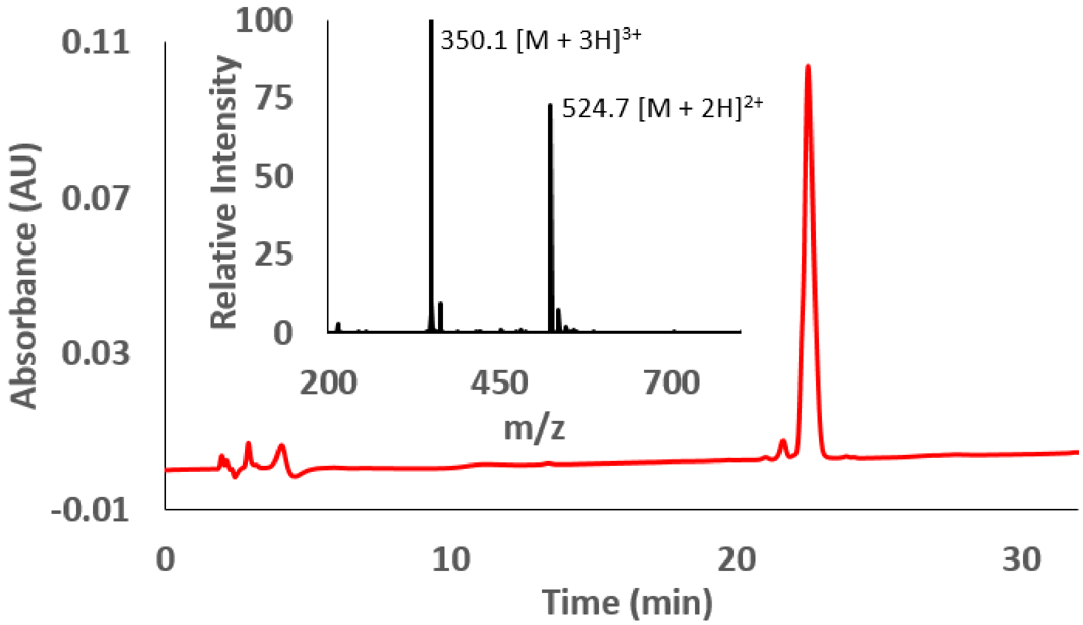


Figure 55: Analytical HPLC trace (with Altantis T3 column) of α-rhamnosylated peptide **14**, $t_R$ = 22.5 min, gradient: 100% A for 2 min then 0 to 20% B over 30 min.

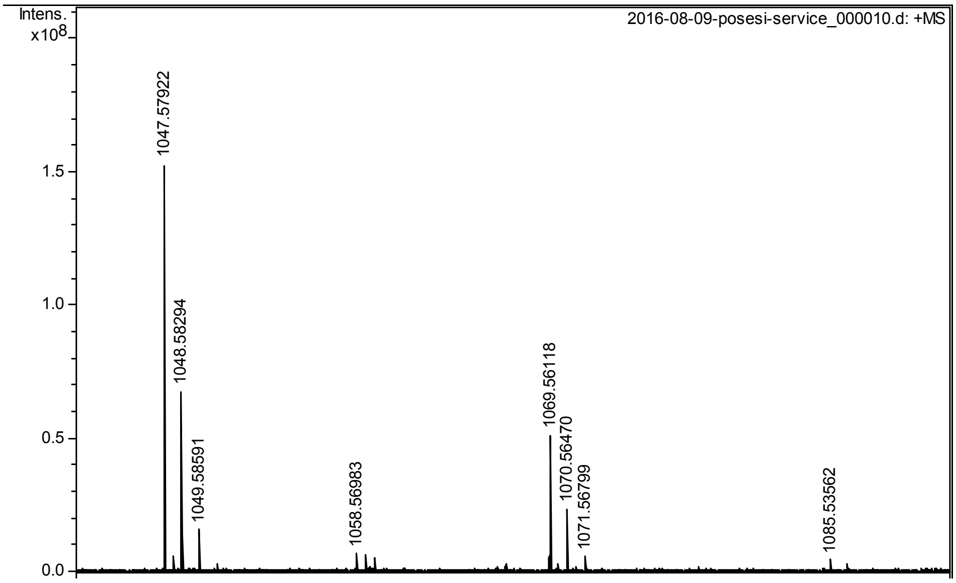


Figure 56: HRMS spectrum for α-rhamnosylated peptide **14**

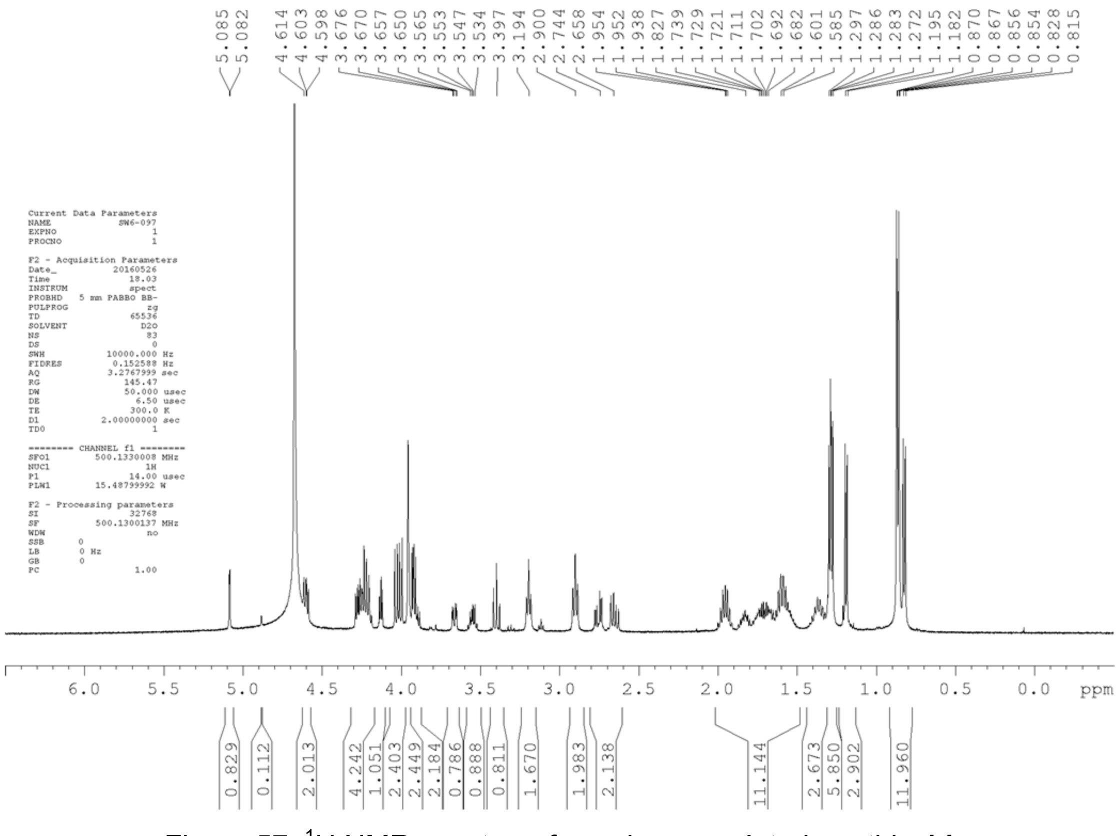

Figure 57: $^{1}$H NMR spectrum for α-rhamnosylated peptide **14**

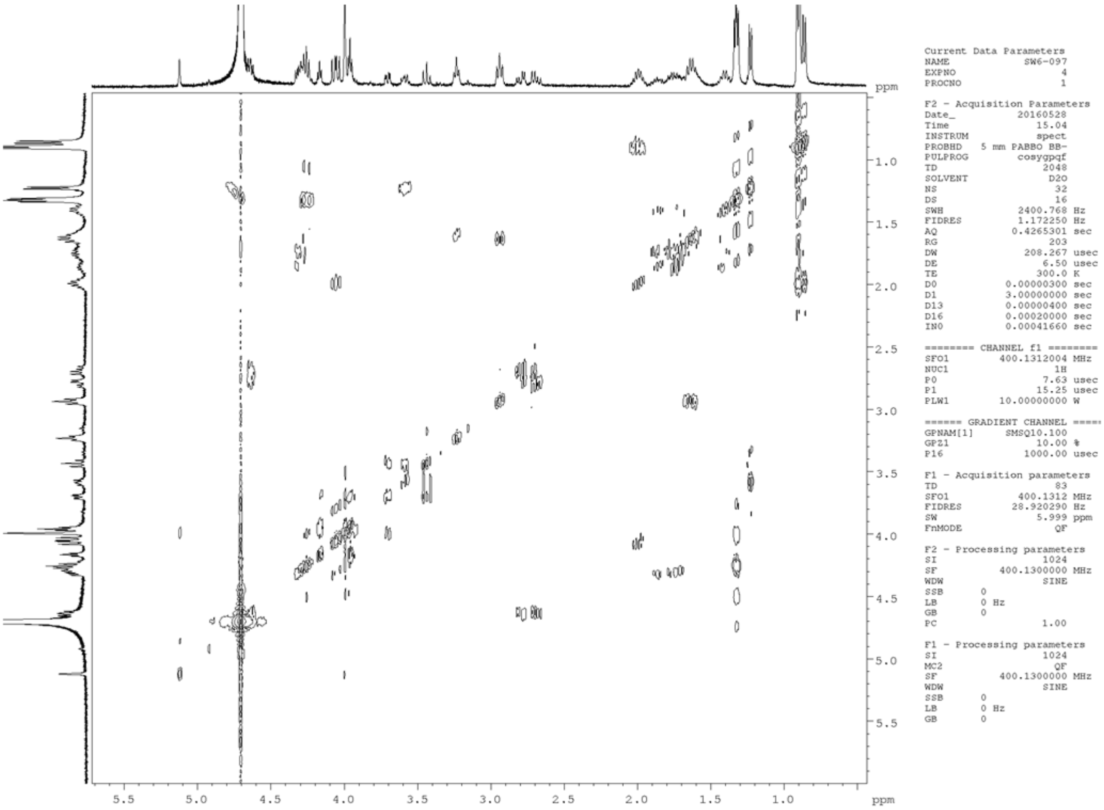


Figure 58: COSY NMR spectrum for α-rhamnosylated peptide **14**

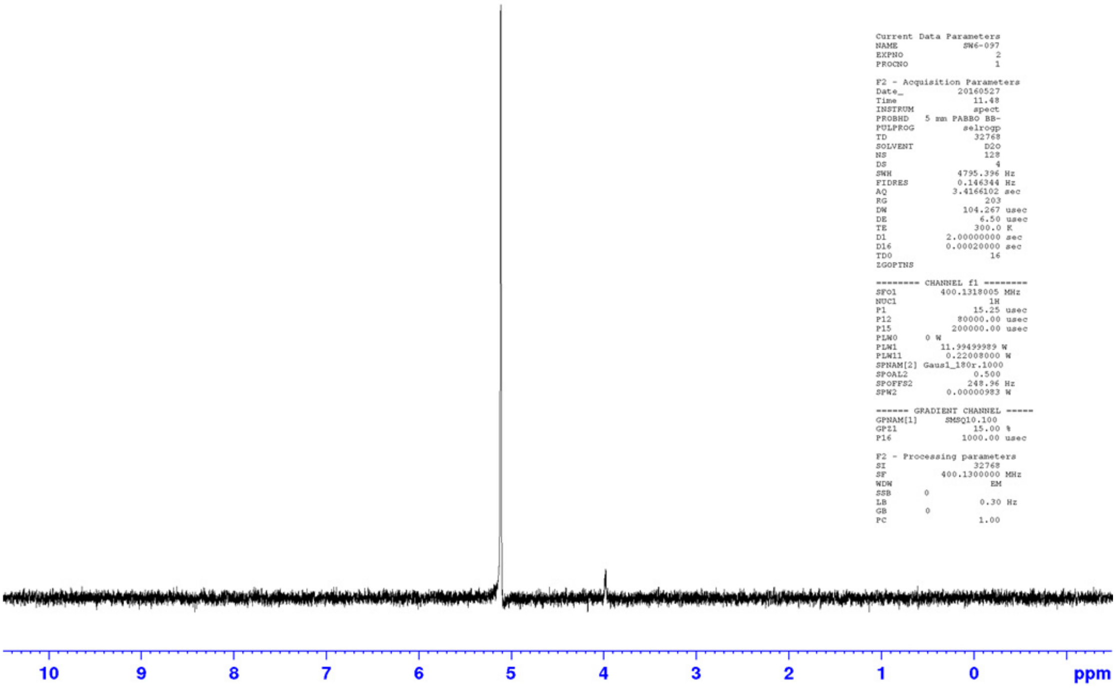


Figure 59: $^{1}$H-$^{1}$H Selective ROSEY NMR spectrum for α-rhamnosylated peptide **14** by irradiation of H1

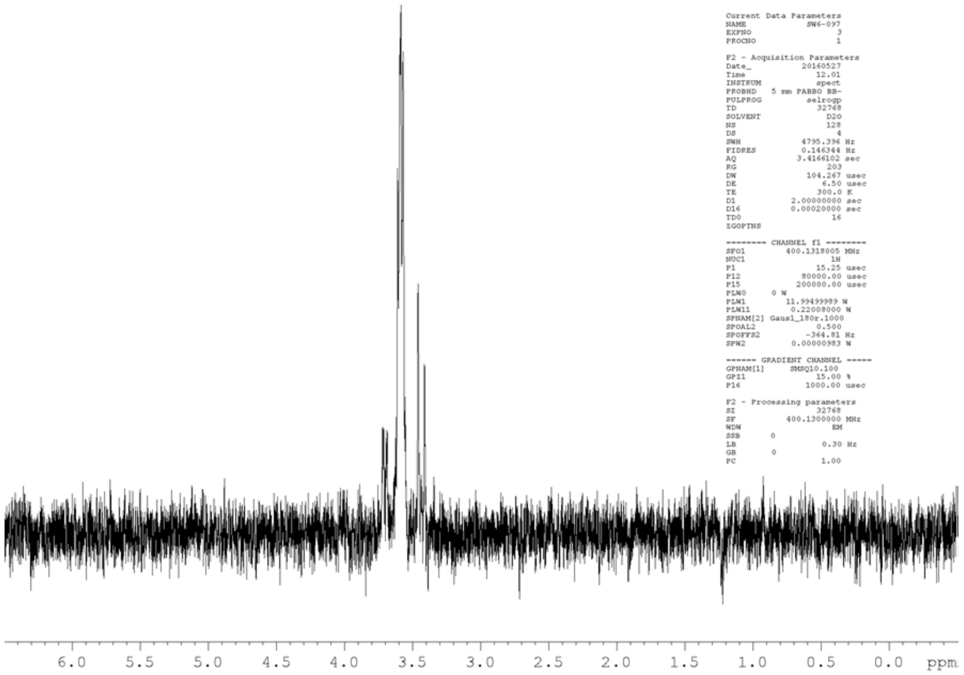


Figure 60: $^{1}H$-$^{1}H$ Selective ROSEY NMR spectrum for α-rhamnosylated peptide **14** by irradiation of H5

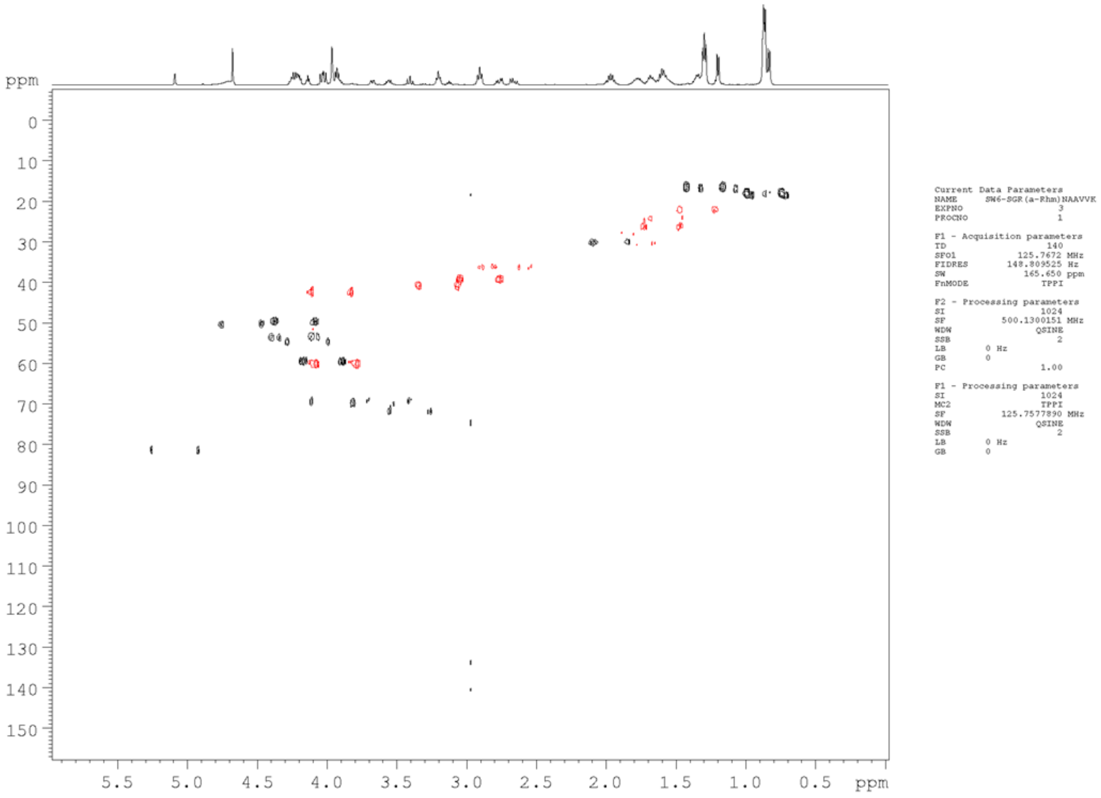


Figure 61: $^{1}H$-$^{13}C$ HSQC without proton decoupling NMR spectrum for α-rhamnosylated peptide **14**

4.2 β-Rhamnosylated Peptide **15**

The synthesis of β-rhamnosylated peptide **15** was conducted on a 22 μmol scale based on Fmoc loading assay after loading the first amino acid. After HPLC purification and lyophilization, the glycopeptide was obtained as a white powder (9.7 mg, 0.7 μmol, 30%). $^{1}$**H NMR** (500 MHz, $D_2O$): 4.89 ($s_{apt}$, 1H, $J_{H1\text{-}C1}$ = 153 Hz, Rha-H1), 4.60 (dd, 1H, Asn-Hα), 4.30 – 3.80 (m, 12H, Arg-Hα, Ser-Hα, 2 x Ala-Hα, Lys-Hα, Gly-Hα$_1$, Gly-Hα$_2$, Rha-H2, 2 x Val-Hα, Ser-Hβ$_1$, Ser-Hβ$_2$), 3.55 (dd, 1H, $J_{2,3}$ = 2.5 Hz, $J_{3,4}$ = 9.6 Hz, Rha-H3), 3.43 (dq, 1H, $J_{4,5}$ = 9.7 Hz, $J_{5,6}$ = 6.3 Hz, Rha-H5), 3.32 ($t_{apt}$, 1H, $J_{3,4}$ = $J_{4,5}$ = 9.9 Hz, Rha-H4), 3.20 (t, 2H, Arg-Hδ), 2.91 (t, 2H, Lys-Hε), 2.75 (dd, 1H, Asn-Hβ$_1$), 2.67 (dd, 1H, Asn-Hβ$_2$), 1.97 (septet, 2H, 2 x Val-Hβ), 1.85 – 1.55 (m, 8H, 2 x Arg-Hβ, 2 x Lys-Hβ, 2 x Arg-Hγ, 2 x Lys-Hδ), 1.40 - 1.25 (m, 8H, 2 x Lys-Hγ, 2 x Ala-C$\underline{H}_3$), 1.20 (d, 3H, $J_{5,6}$ = 6.0 Hz, Rha-H6), 0.88 – 0.80 (m, 12H, 4 x Val-C$\underline{H}_3$) ppm. **HRMS**: (+ESI) m/z, $[M + H]^+$, Calcd: 1047.5793, Found: 1047.5799.

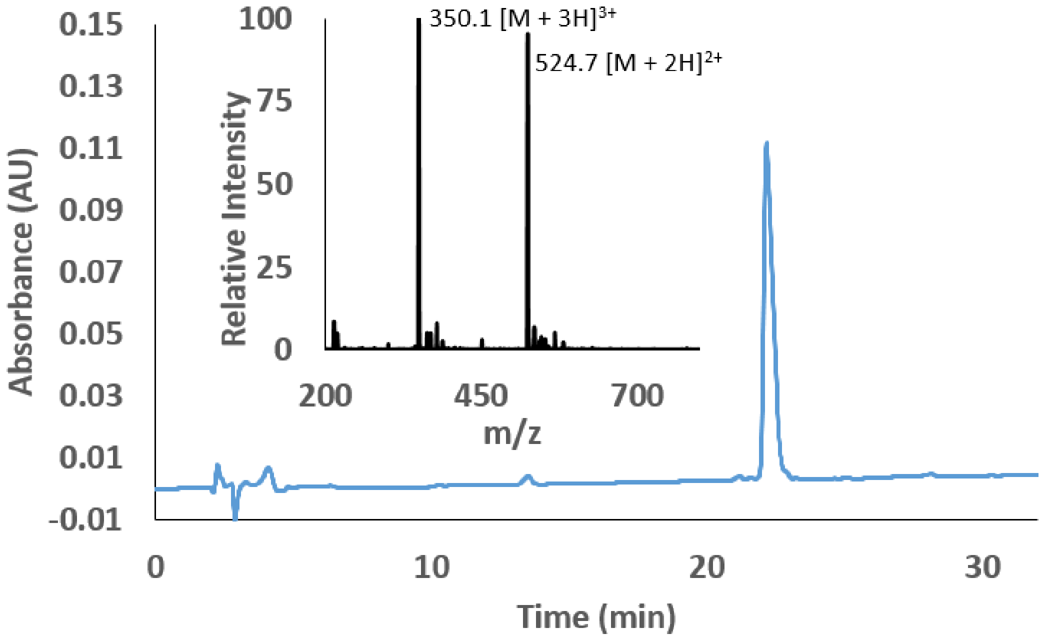


Figure 62: Analytical HPLC trace (with Altantis T3 column) of β-rhamnosylated peptide **15**, $t_R$ = 22.2 min, gradient: 100% A for 2 min then 0 to 20% B over 30 min.

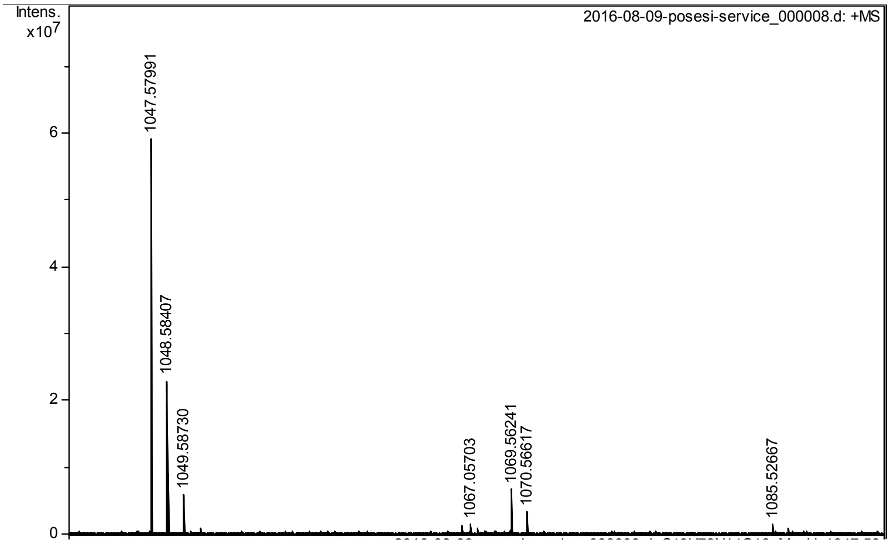


Figure 63: HRMS spectrum for β-rhamnosylated peptide **15**

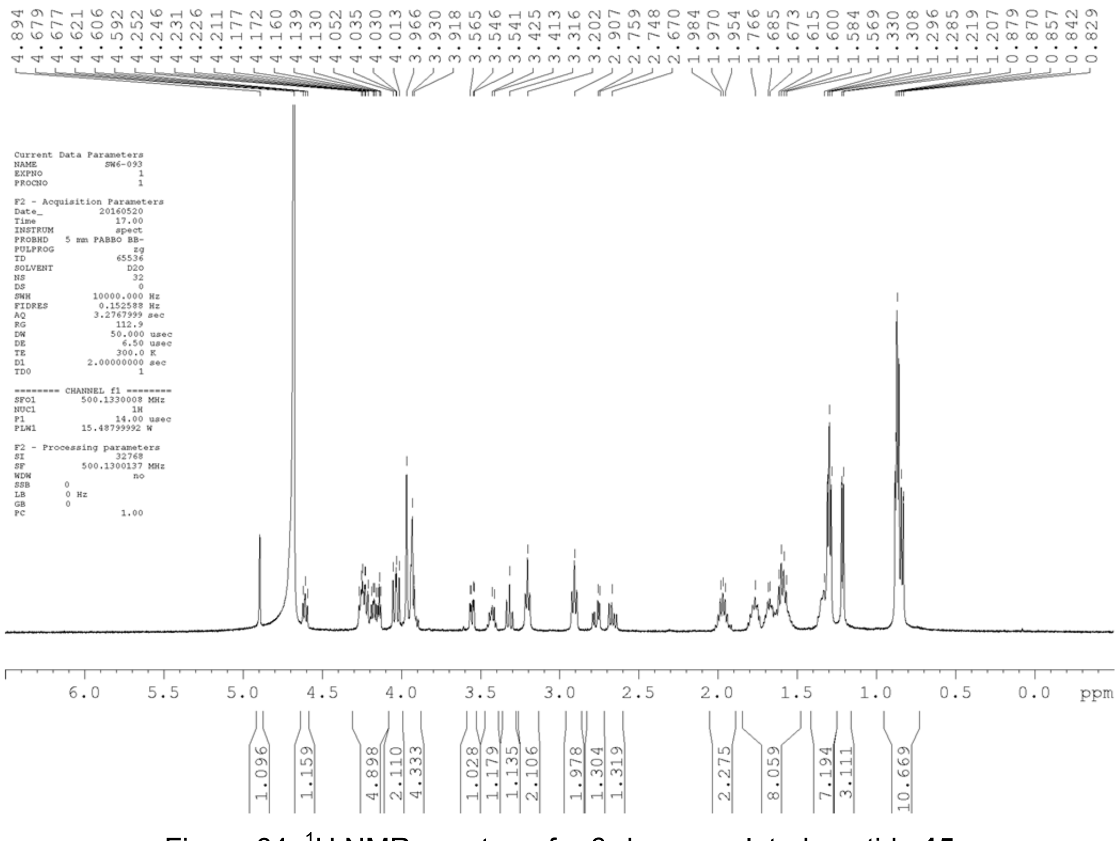


Figure 64: $^{1}$H NMR spectrum for β-rhamnosylated peptide **15**

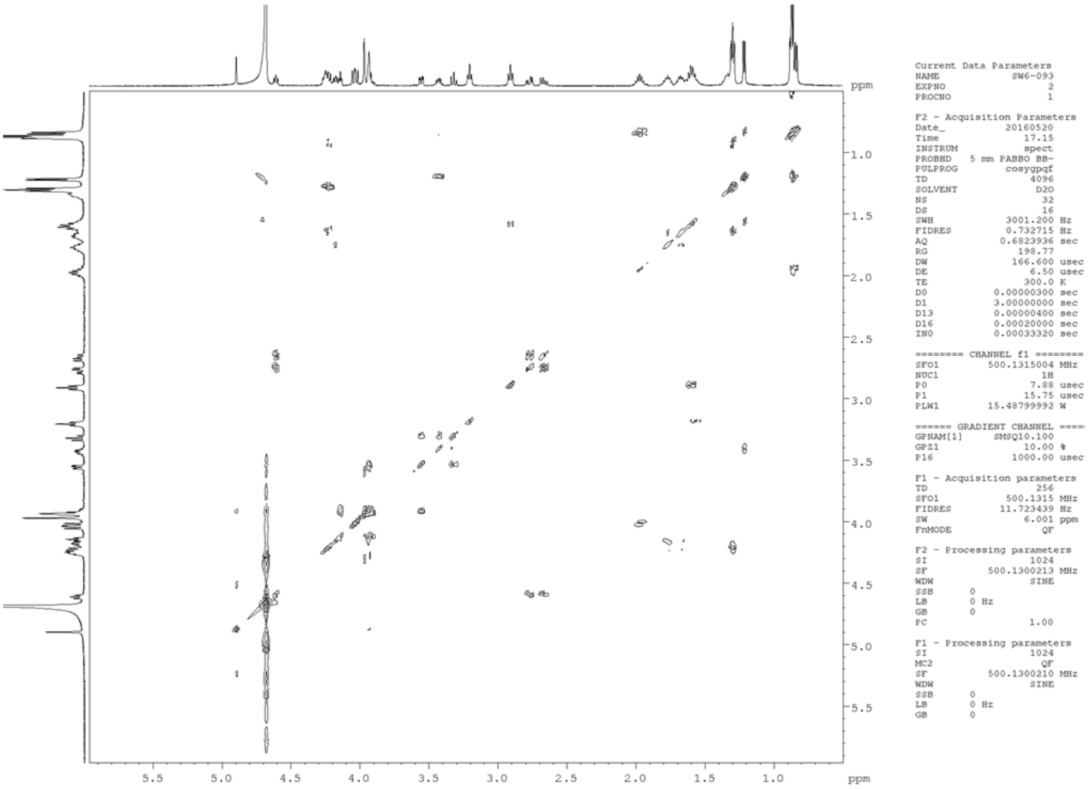


Figure 65: COSY NMR spectrum for β-rhamnosylated peptide **15**

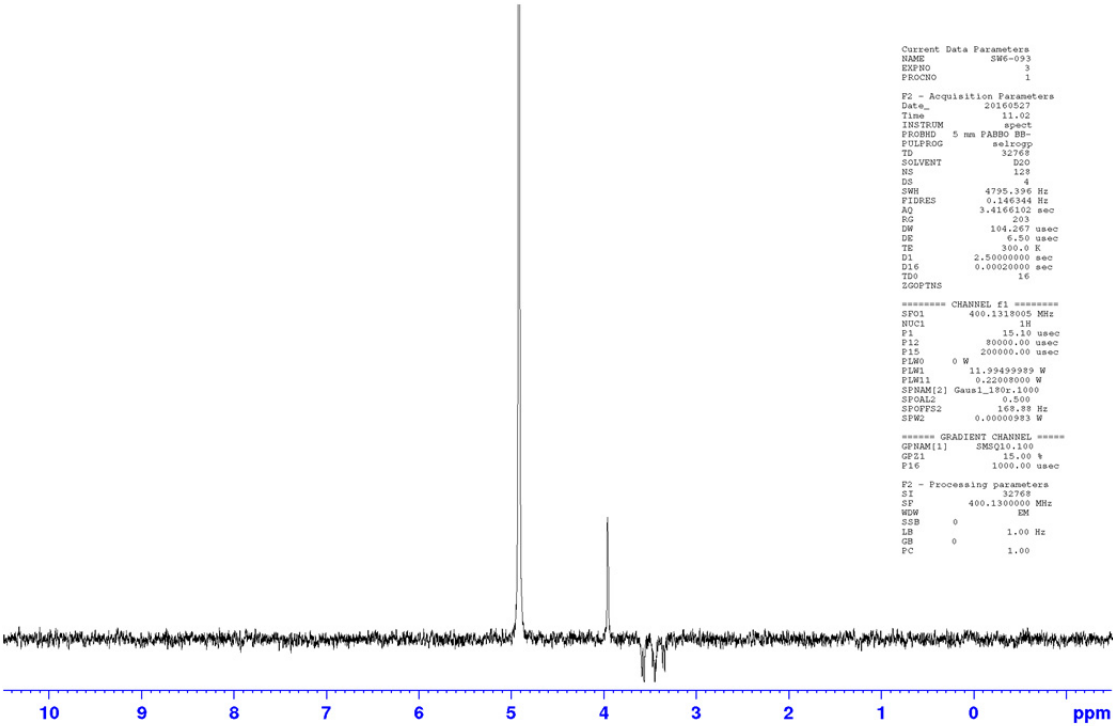


Figure 66: $^{1}$H-$^{1}$H Selective ROSEY NMR spectrum for β-rhamnosylated peptide **15** by irradiation of H1

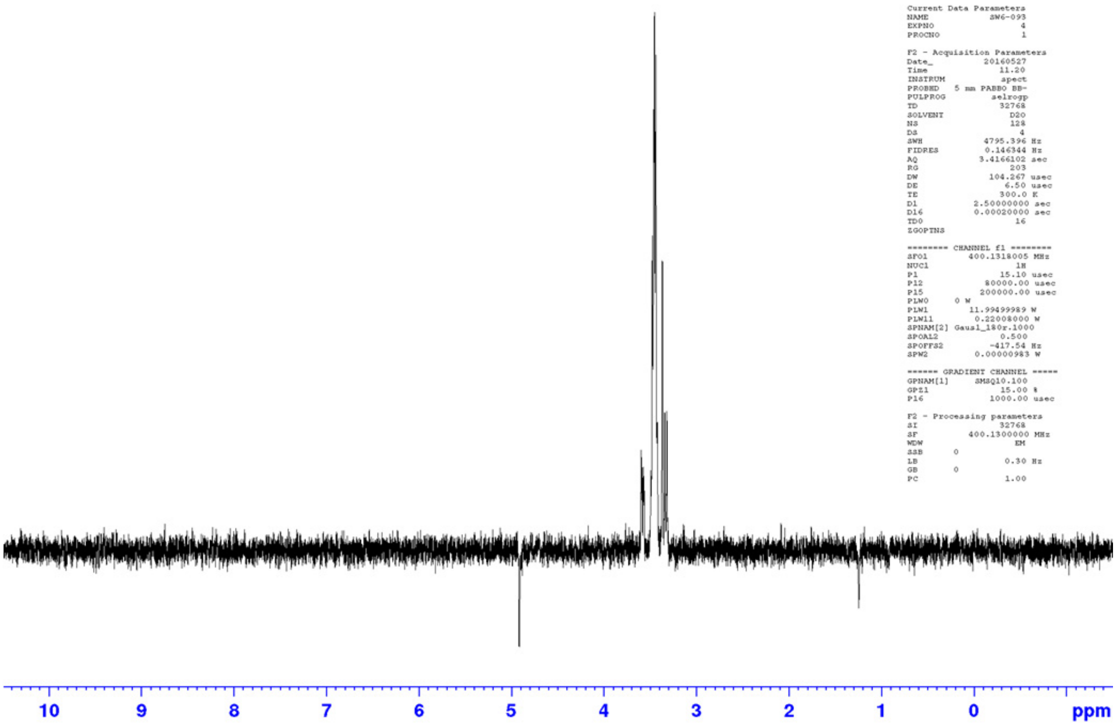


Figure 67: $^{1}H$-$^{1}H$ Selective ROSEY NMR spectrum for β-rhamnosylated peptide **15** by irradiation of H5

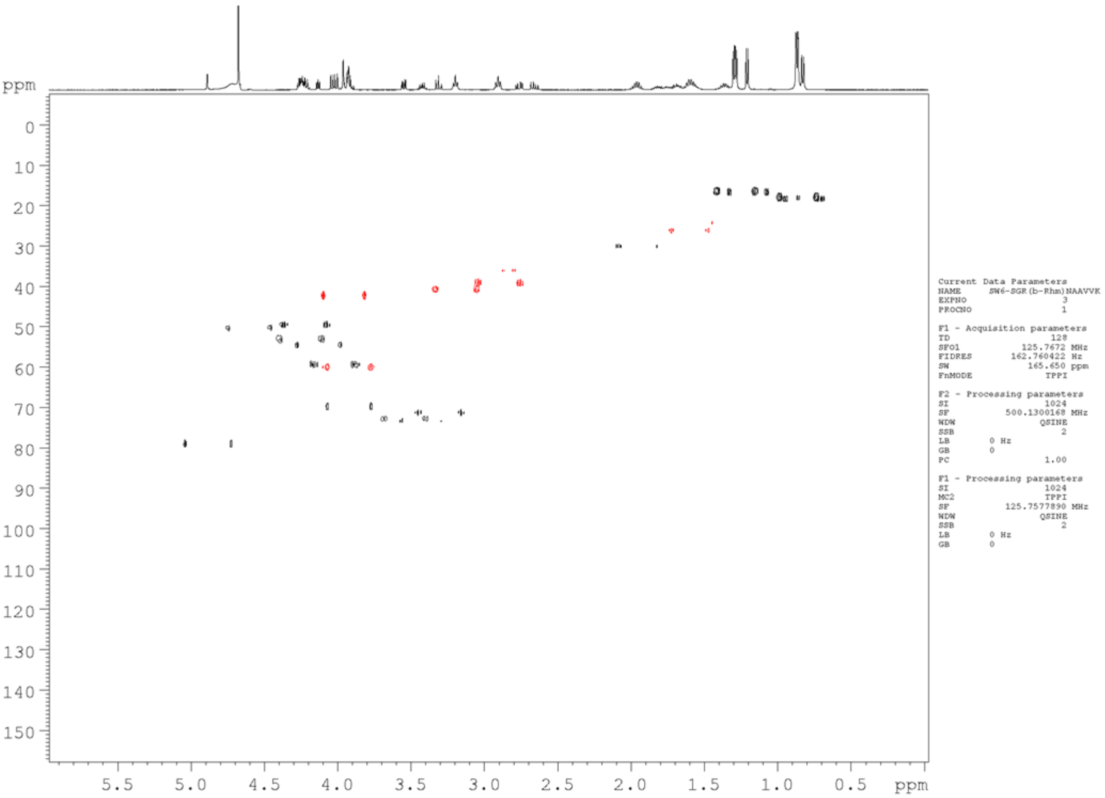


Figure 68: $^{1}H$-$^{13}C$ HSQC without proton decoupling NMR spectrum for β-rhamnosylated peptide **15**

4.3 Resolution of Glycopeptides **14** and **15** with HPLC system

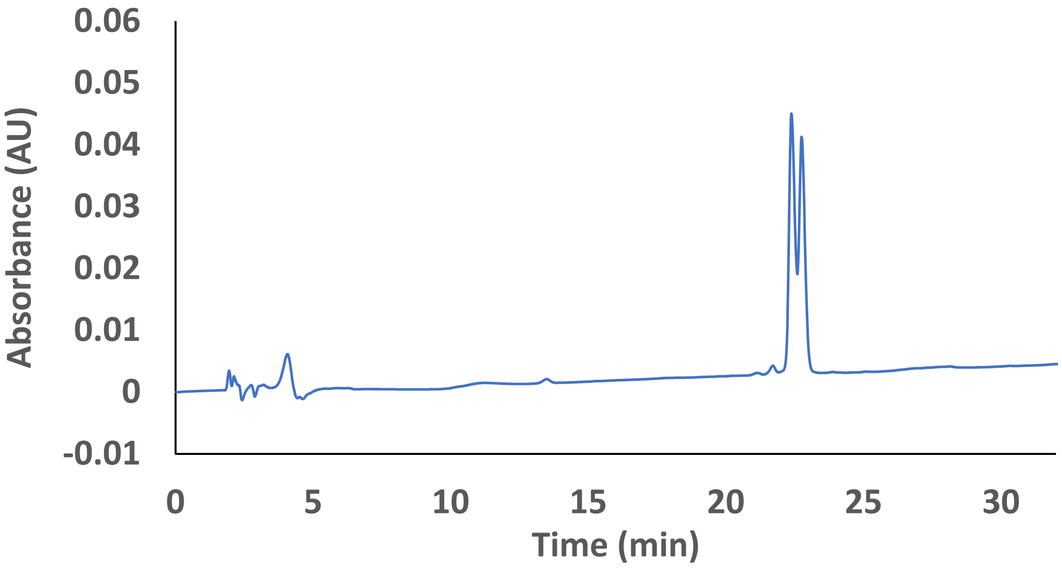


Figure 69: Analytical HPLC trace (with Altantis T3 column) of co-injection of both α-rhamnosylated peptide **14** and β-rhamnosylated peptide **15**, gradient: 100% A for 2 min then 0 to 20% B over 30 min.

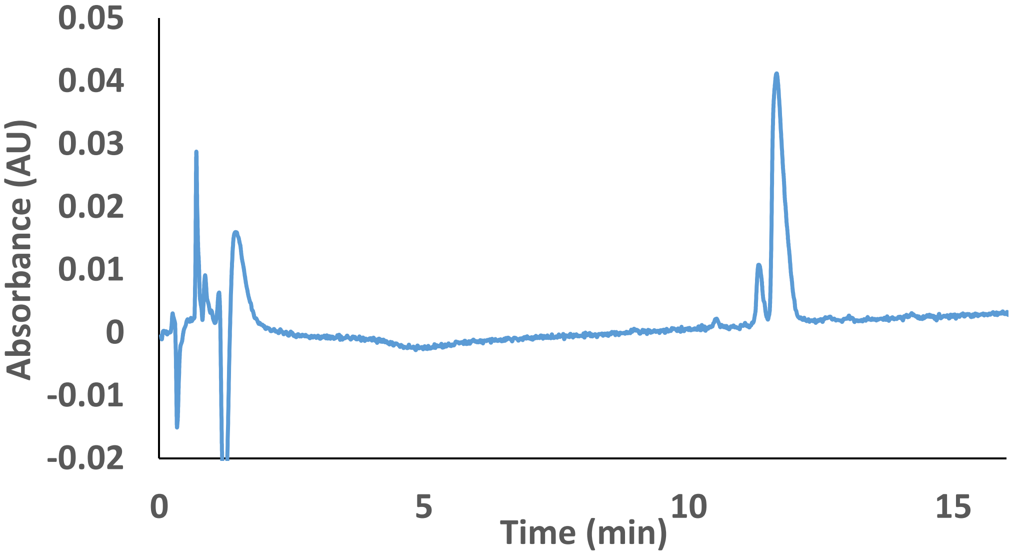


Figure 70: Analytical HPLC trace (with fluorophenyl column) of α-rhamnosylated peptide **14** ($t_R$ = 11.7 min), gradient: 100% A for 2 min then 0 to 20% B over 30 min. Please note this sample contains 11% β-rhamnosylated peptide **15**, $t_R$ = 11.3 min)

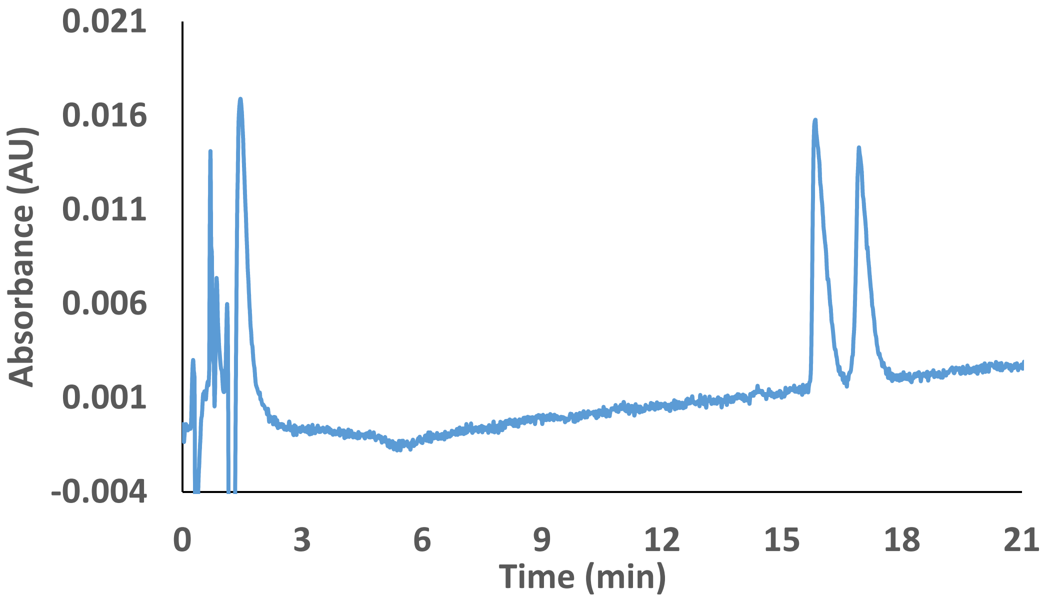


Figure 71: Analytical HPLC Trace with fluorophenyl column of co-injection of both α-rhamnosylated peptide **14** ($t_R$ = 16.9 min) and β-rhamnosylated peptide **15** ($t_R$ = 15.8 min), gradient:100% A for 1 min then 0 to 6.7% B over 20 min.

Analytical HPLC purification was performed on the α-rhamnosylated peptide **14** (α:β 9:1 as in Figure 77) to obtain pure α-rhamnosylated peptide **14** for MS/MS experiments.

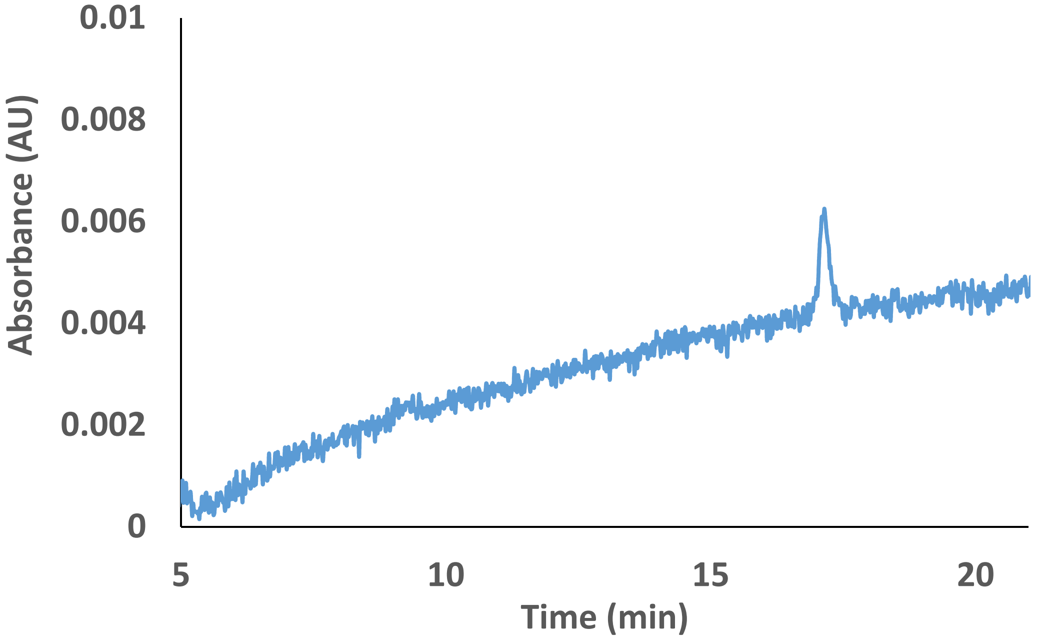


Figure 72: Analytical HPLC Trace with fluorophenyl column of α-rhamnosylated peptide **14** after second purification (retention time = 17.1 min), gradient:100% A for 1 min then 0 to 6.7% B over 20 min.

# 5. Analysis of Anomerization under Acidic and Basic Conditions

5.1 Analysis of **1** and **2** during conventional Pbf-deprotection and deacetylation sequence

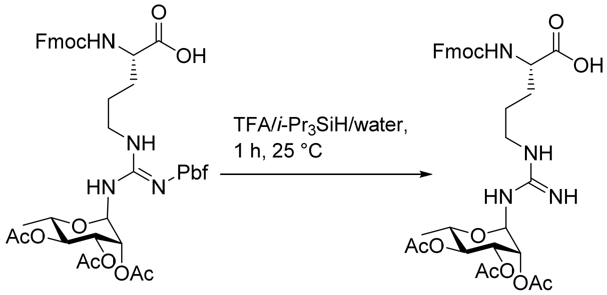


**1** **S1**

Fmoc-Arg(α-Rha,Pbf)-OH **1** (92 mg, 0.1 mmol) was suspended in TFA/*i*-$Pr_3SiH/H_2O$ (18:1:1, v/v/v, 10 ml) at 25 °C. The mixture was stirred vigorously for 90 min before concentrating under reduced pressure. The residue was purified by RP-HPLC to yield Fmoc-Arg[α-Rha(OAc)$_3$]-OH **S1** (58 mg, 0.075 mmol, 75%) as white solid after lyophilization.. $^1$**H NMR** (500 MHz, d6-acetone): 7.88 (d, 2H, $J$ = 7.6 Hz, Ar-H), 7.74 (dd$_{apt}$, 2H, $J_1$ = 7.2 Hz, $J_2$ = 4.4 Hz, Ar-H), 7.43 (t, 2H, $J$ = 7.4 Hz, Ar-H), 7.33 (td, 2H, $J_1$ = 11.2 Hz, $J_1$ = 1.1 Hz, Ar-H), 6.88 (d, 1H, $J$ = 8.4 Hz, Fmoc-NH), 5.51 (dd, 1H $J_{2,3}$ = 3.4 Hz, $J_{3,4}$ = 9.5 Hz, H-3), 5.42 (s$_{apt}$, 1H, H-1), 5.36 (d$_{apt}$, 1H, $J_{2,3}$ = 3.4 Hz, H-2), 5.00 (t$_{apt}$, $J_{3,4}$ = $J_{4,5}$ = 9.6 Hz, H-4), 4.36 (m, 2H, FmocCH-C$\underline{H_2}$-O-CO), 4.28 (m, 1H, FmocC$\underline{H}$-$CH_2$-O-CO), 4.26 (m, 1H, Arg-Hα), 4.03 (dq, 1H, $J_{4,5}$ = 9.3 Hz, $J_{5,6}$ = 6.4 Hz, H-5), 3.50 (m, 2H, Arg-Hδ), 2.12 (s, 3H, OAc), 2.02 (s, 3H, OAc), 1.94 (s, 3H, OAc), 1.87 (m, 4H, 2 x Arg-Hβ, 2 x Arg-Hγ), 1.21 (d, 3H, $J_{5,6}$ = 6.2 Hz, H-6) ppm.

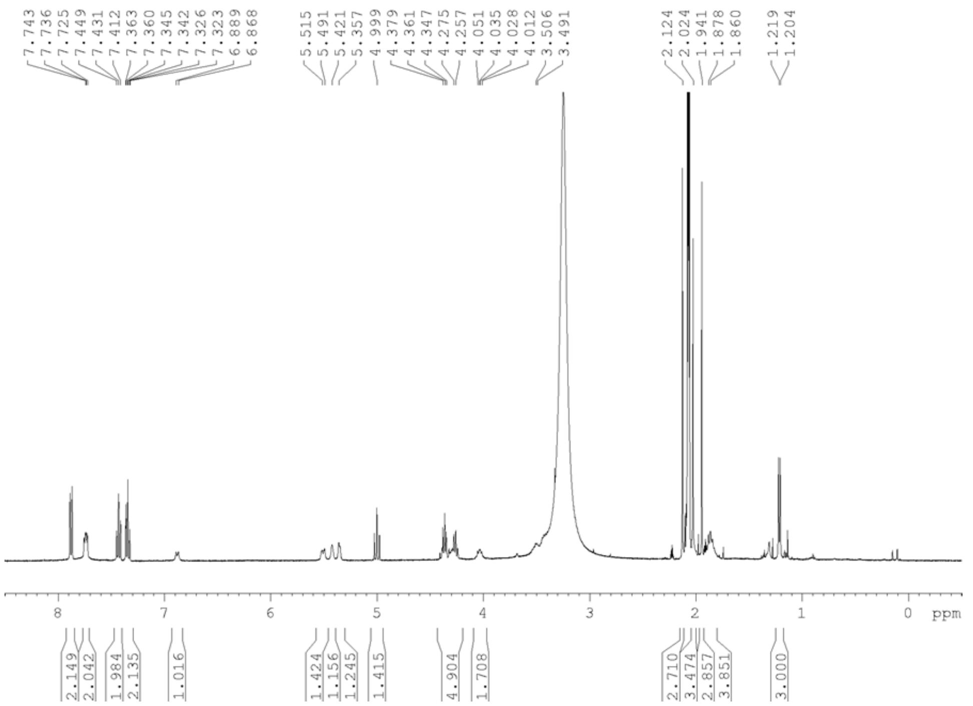


Figure 73: $^{1}$H NMR spectrum for **S1**

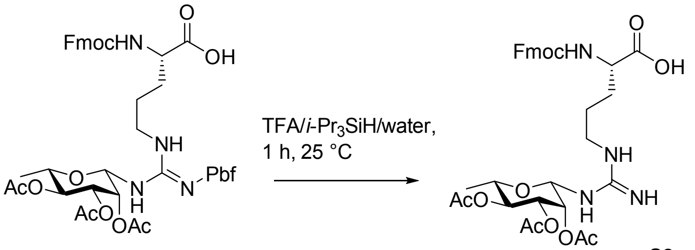


**2** **S2**

Fmoc-Arg(β-Rha,Pbf)-OH **2** (200 mg, 0.22 mmol) was suspended in TFA/*i*-$Pr_3SiH/H_2O$ (18:1:1, v/v/v, 10 ml) at 25 °C. The mixture was stirred vigorously for 90 min before concentrating under reduced pressure. The residue was purified by RP-HPLC to yield Fmoc-Arg[β-Rha(OAc)$_3$]-OH **S2** (130 mg, 0.16 mmol, 72%) as white solid after lyophilization. **$^1$H NMR** (500 MHz, d6-acetone): δ 7.87 (d, 2H, $J$ = 7.6 Hz, Ar-H), 7.74 (dd$_{apt}$, 2H, $J_1$ = 6.8 Hz, $J_2$ = 5.1 Hz, Ar-H), 7.43 (t, 2H, $J$ = 7.3 Hz, Ar-H), 7.33 (td, 2H, $J_1$ = 11.2 Hz, $J_2$ = 1.2 Hz, Ar-H), 6.89 (d, 1H, $J$ = 8.7 Hz, Fmoc-NH), 5.48 (d$_{apt}$, 1H, $J_{1,2}$ = 1.1 Hz, $J_{2,3}$ = 3.2 Hz, H-2), 5.40 (dd$_{apt}$, 1H, $J_{1,2}$ = 1.0 Hz, $J_{1,NH}$ = 8 Hz, H-1), 5.08 (dd, 1H $J_{2,3}$ = 3.3 Hz, $J_{3,4}$ = 10.0 Hz, H-3), 5.00 (t$_{apt}$, $J_{3,4}$ = $J_{4,5}$ = 10.0 Hz, H-4), 4.36 (m, 2H, FmocCH-C<u>H$_2$</u>-O-CO), 4.26 (m, 1H, FmocC<u>H</u>-$CH_2$-O-CO), 4.23 (m, 1H, Arg-Hα), 3.88 (dq, 1H, $J_{4,5}$ = 9.6 Hz, $J_{5,6}$ = 6.2 Hz, H-5), 3.50 (m, 2H, Arg-Hδ), 2.22 (s, 3H, OAc), 2.04 (s, 3H, OAc), 1.92 (s, 3H, OAc), 1.87 (m, 4H, 2 x Arg-Hβ, 2 x Arg-Hγ), 1.21 (d, 3H, $J_{5,6}$ = 6.1 Hz, H-6) ppm.

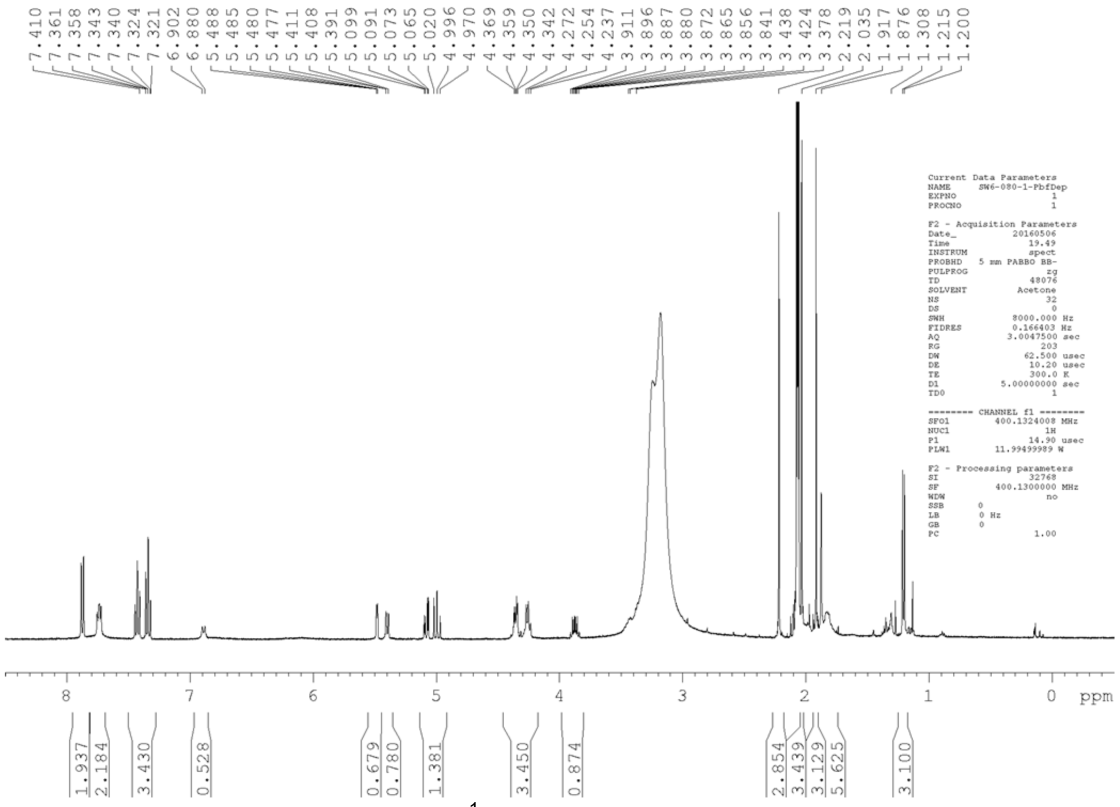

Figure 74: $^{1}$H NMR spectrum for **S2**

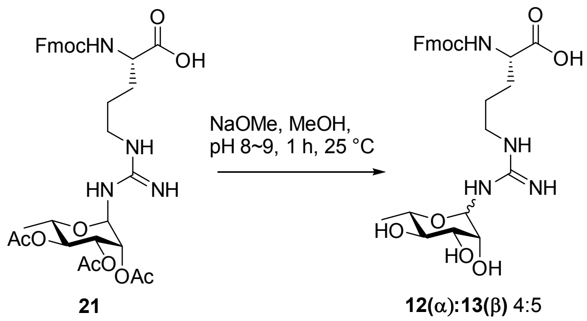

Fmoc-Arg[α-Rha(OAc)$_3$]-OH **21** (58 mg, 0.075 mmol) was dissolved in dry MeOH (5 mL). NaOMe (0.5 M in MeOH, 0.1 mL, 0.200 mmol) was added into the solution to attain a final pH of ~8 (as judged by moist universal indicator strips). The mixture was stirred at 25 °C. After 30 min, the reaction was judged to be complete (monitoring with UPLC-MS). The solution was adjusted to pH 4 by careful addition of TFA and then concentrated under reduced pressure. The residue was purified through RP-HPLC to afford a mixture of compounds **12** (α anomer) and **13** (β anomer) in a 4:5 ratio (23 mg, 0.03 mmol, 46%). See $^{1}$H NMR spectrum of the anomeric mixture below.

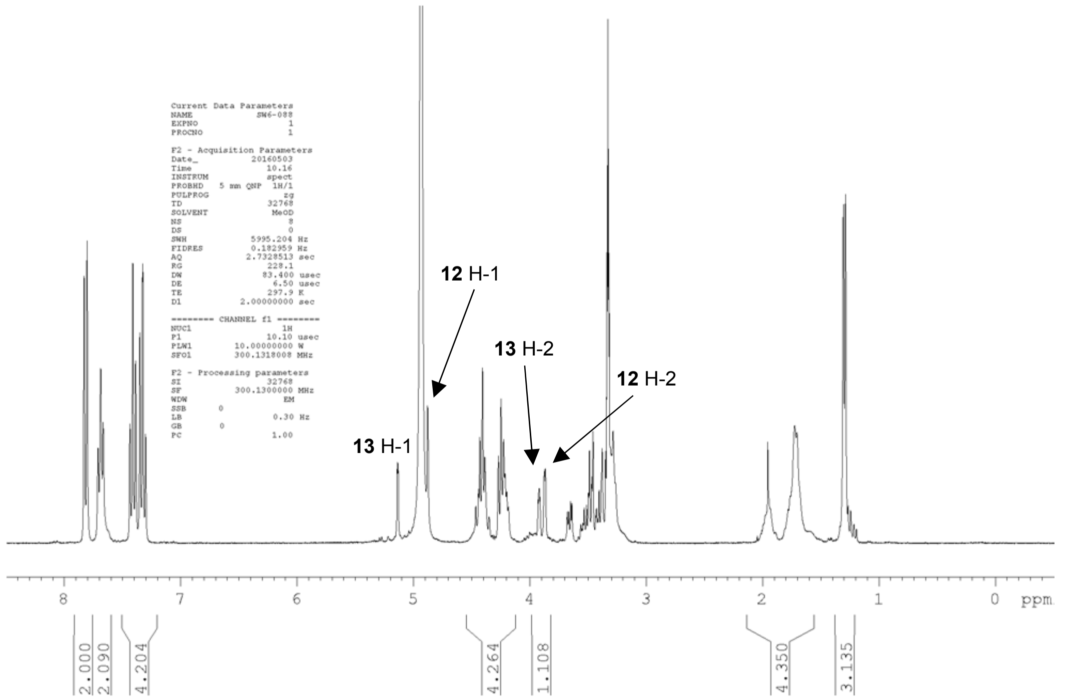


Figure 75: $^{1}$H NMR spectrum for Zemplén deprotection of **21**, affording a mixture of **12** and **13** in a ratio of 4:5

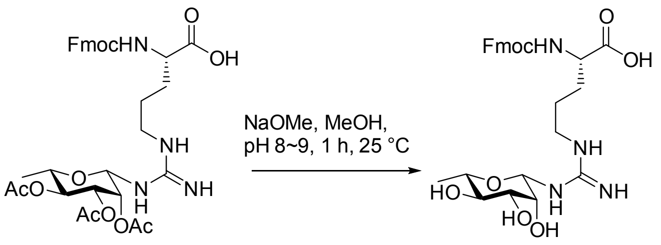


**22** **13**

Please refer to page S41 for treatment of β-rhamnoside **22** with NaOMe/MeOH.

## 5.2 Anomerization of α-Rhamnosylated Glycopeptide during Deacetylation with Hydrazine (off resin)

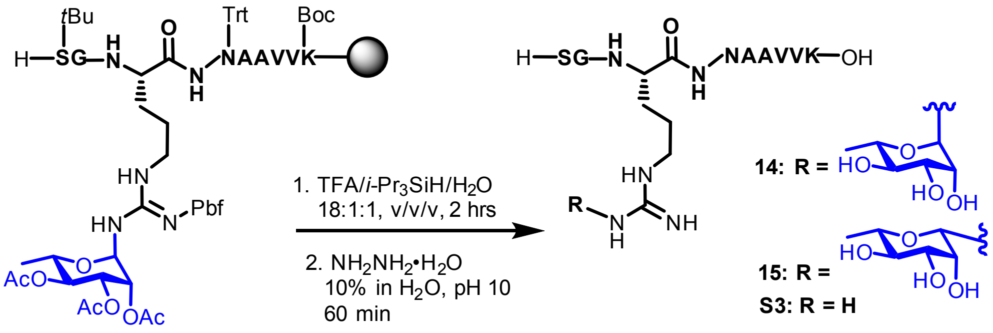


**19**

After washing thoroughly with dichloromethane (10 x 5 mL), the resin-bound α-rhamnosylated peptide **19** was suspended in TFA/*i*-$Pr_3SiH/H_2O$ (18:1:1 v/v/v) for 2 h. After filtration, the filtrate was concentrated by blowing with a gentle flow of nitrogen gas. The residue was then re-dissolved in hydrazine monohydrate (10 vol% in water) to attain a final pH of 10. The resulting solution was stirred at 25 °C for 30 min. After complete deacetylation (monitored by UPLC-MS) the reaction was quenched with formic acid (100 µL). The resulting mixture was concentrated and subjected to RP-HPLC to yield a mixture of α-rhamnosylated peptide **14**, β-rhamnosylated peptide **15** (α: β = 1:1), as well as significant quantities of deglycosylated peptide **S3**.

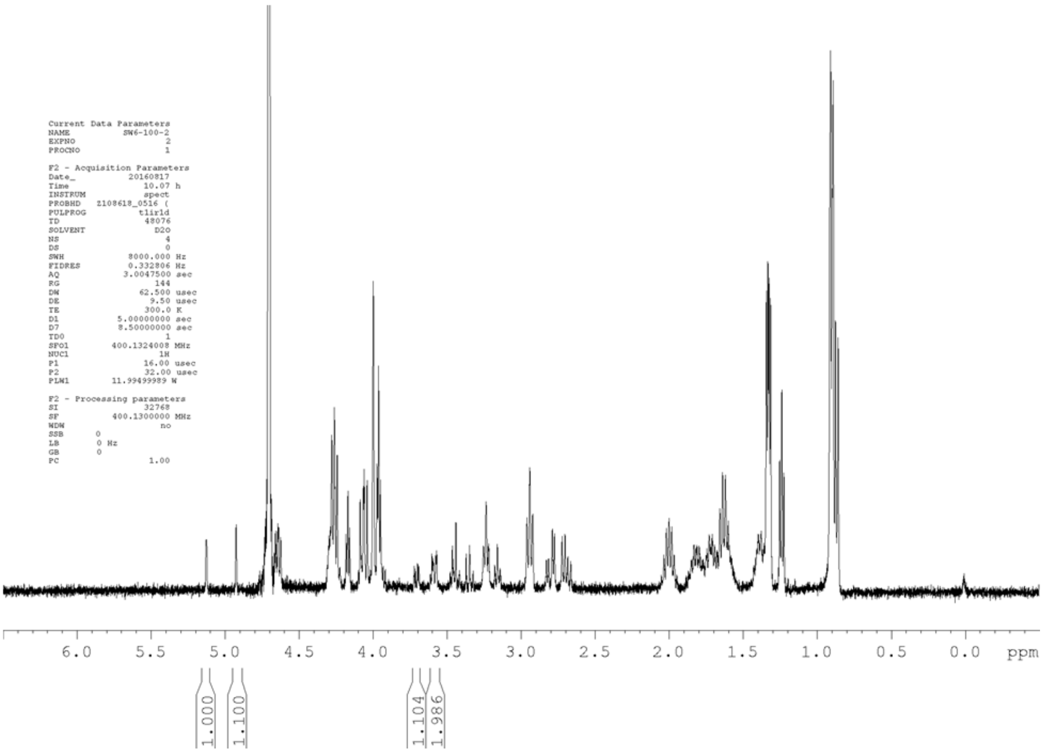

Figure 76: $^1$H NMR spectrum after acidolytic side chain deprotection and then hydrazine deacetylation of resin-bound glycopeptide **19**. The spectrum was a 1:1 mixture of α-rhamnosylated peptide **14** and β-rhamnosylated peptide **15**.

## 5.3 Anomerization of α-Rhamnosylated Glycopeptide during Deacetylation with Hydrazine (on resin)

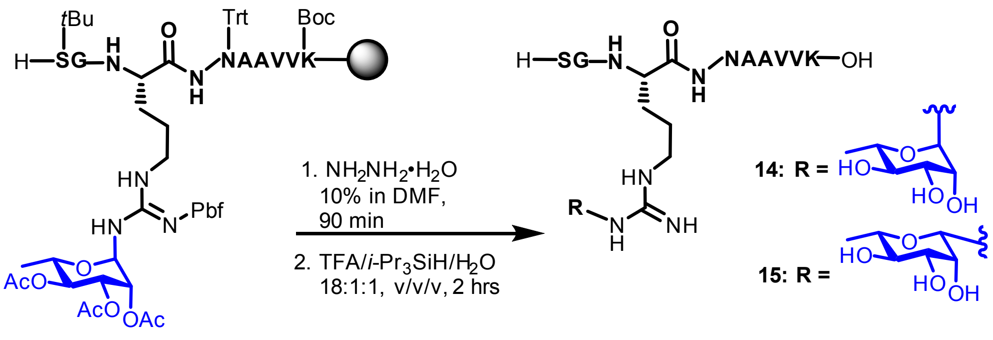


**19**

After washing thoroughly with dichloromethane (10 x 5 mL), the resin-bound α-rhamnosylated peptide **19** was agitated in hydrazine monohydrate (10 vol% in water) (3 ml, 90 min). The resin was then washed thoroughly with DMF (5 x 3 mL), MeOH (5 x 3 mL), DMF (5 x 3 mL) and dichloromethane (10 x 3 ml) and then suspended in TFA/*i*-$Pr_3SiH/H_2O$ (18:1:1 v/v/v) for 2 h. After filtration, the filtrate was concentrated by blowing with a gentle flow of nitrogen gas. The residue was purified through RP-HPLC to yield a mixture of α-rhamnosylated peptide **14** and β-rhamnosylated peptide **15** (α: β = 1:1).

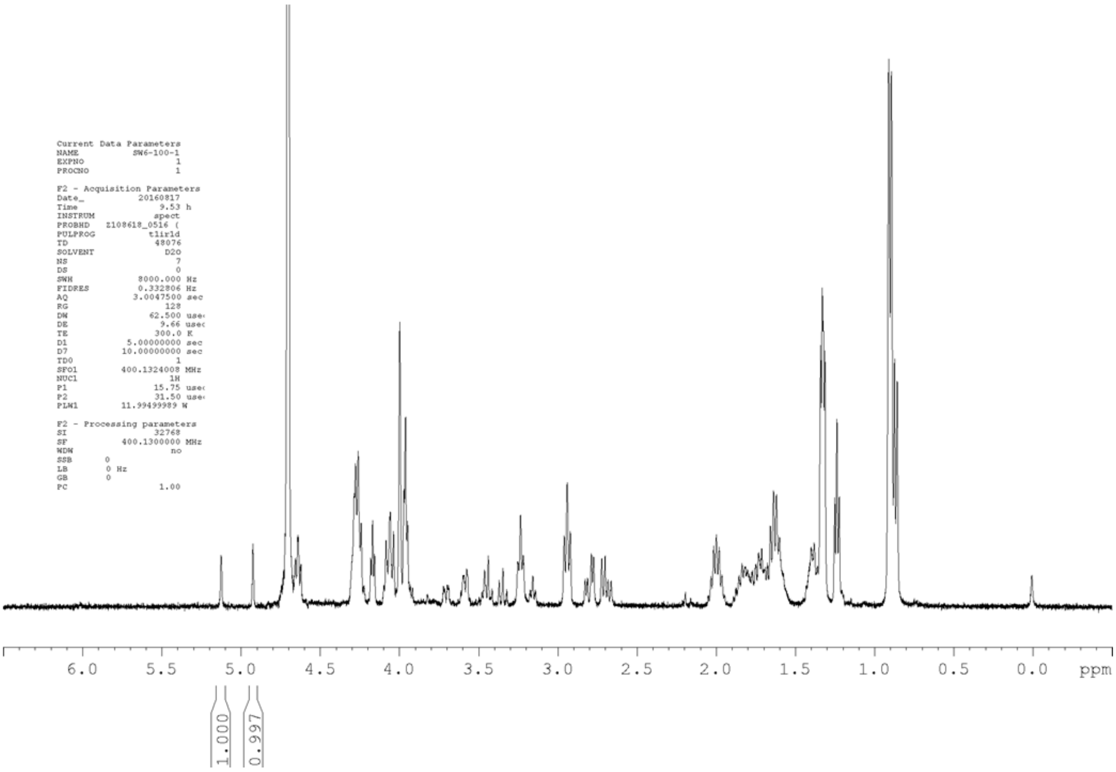


Figure 77: $^{1}$H NMR spectrum after hydrazine deacetylation and then acidolytic cleavage of resin-bound glycopeptide **19**. The spectrum shows a 1:1 mixture of α-rhamnosylated peptide **14** and β-rhamnosylated peptide **15**.

## 5.4 Examination of β-Rhamnosylated Glycopeptide during Deacetylation with Hydrazine (off resin)

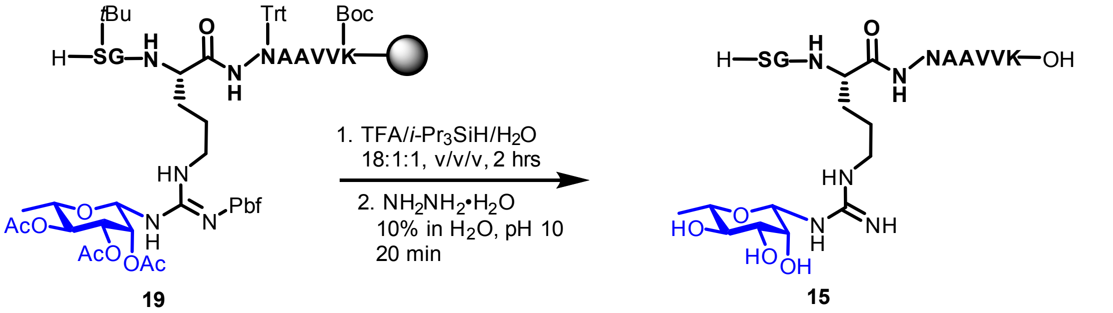


After washing thoroughly with dichloromethane (10 x 5 mL), the resin-bound β-rhamnosylated peptide **20** was suspended in TFA/*i*-$Pr_3SiH/H_2O$ (18:1:1 v/v/v) for 2 h.

After filtration, the filtrate was concentrated by blowing with a gentle flow of nitrogen gas. The residue was then re-dissolved in hydrazine monohydrate (10 vol% in water) to attain a final pH of 10. The resulting solution was stirred at 25 °C for 30 min. After complete deacetylation (monitored by UPLC-MS) the reaction was quenched with formic acid (100 µL). The resulting mixture was concentrated and purified through RP-HPLC to yield pure β-rhamnosylated peptide **14**.

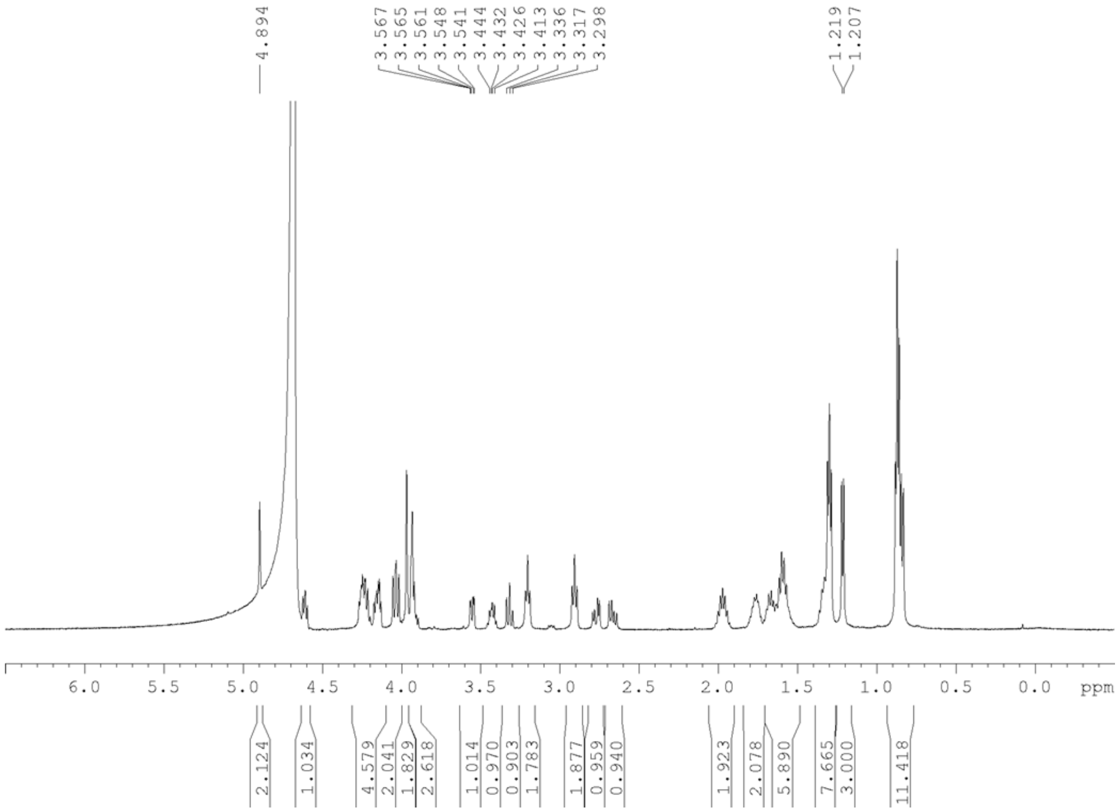


Figure 78: $^{1}$H NMR spectrum after acidolytic side chain deprotection/cleavage from the resin and then hydrazine deacetylation of resin-bound glycopeptide **20**. The spectrum was identical to that for β-rhamnosylated peptide **14**.

5.5 Trapping of Imine Intermediate during Glycopeptide Anomerization

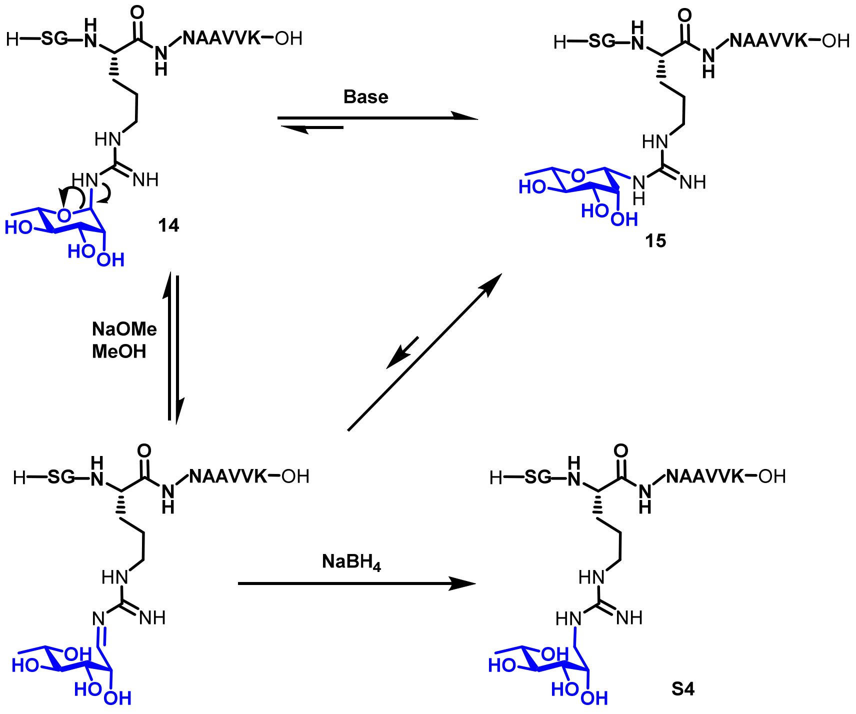


An anomeric mixture of α-rhamnosylated peptide **14** and β-rhamnosylated peptide **15** (1:1, 1.5 mg, obtained in Section 5.3) was suspended in MeOH (1 ml). NaOMe (0.5 M in MeOH, 0.1 ml) was added followed by $NaBH_4$ (0.1 g). The mixture was incubated at 25 $^{o}$C for 4 hr The crude mixture was desalted by filtering through a pad of C-18 silica and the filtrate was analysed by FTICR to detect reduced glycopeptide **S4** as one component of the mixture containing α-anomer **14**, β-anomer **15** and deglycosylated peptide **S3**. **HRMS**: (+ESI) m/z, $[M + 2H]^{2+}$, Calcd: 525.30111, Found: 525.30124.

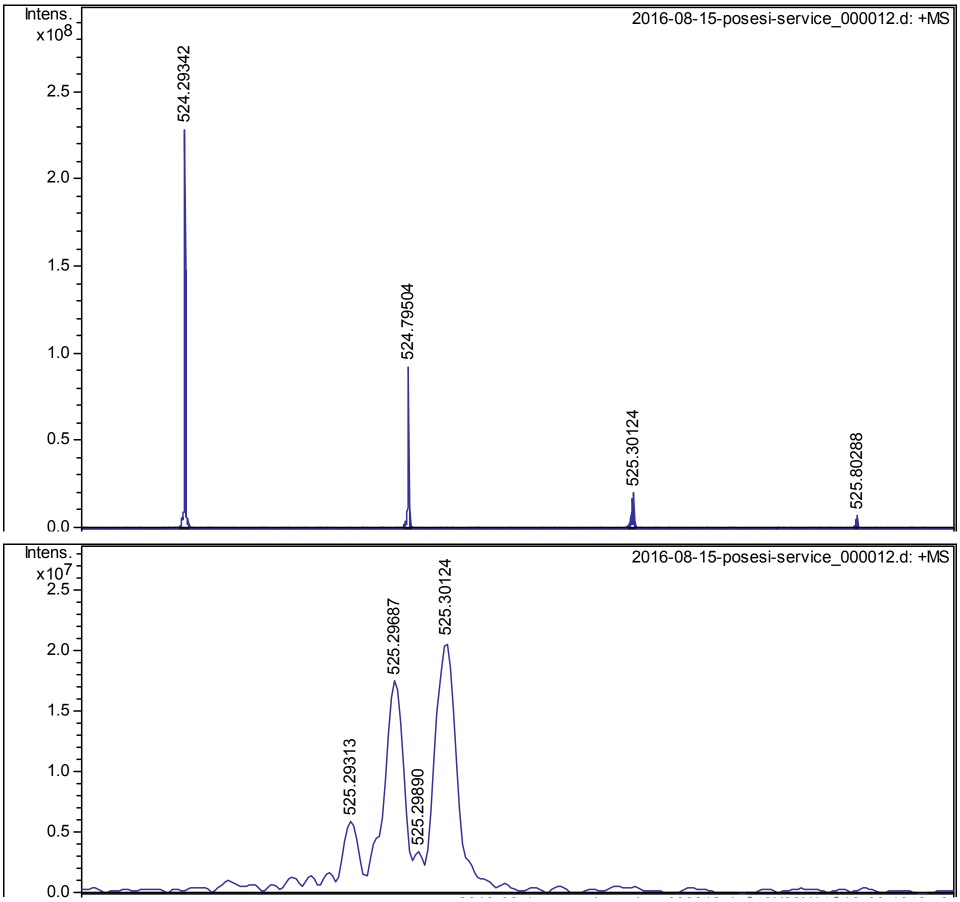


Figure 79: Expansion of HRMS spectrum of the reaction mixture. m/z 525.2931, 525.2969 and 525.2989 correspond to the isotopes of glycopeptide **14** or **15**; m/z 525.30124 corresponds to the reduced peptide **S4**.

## 5.6 Anomerization of α-Rhamnosylated Glycopeptide during Treatment with NaOMe.

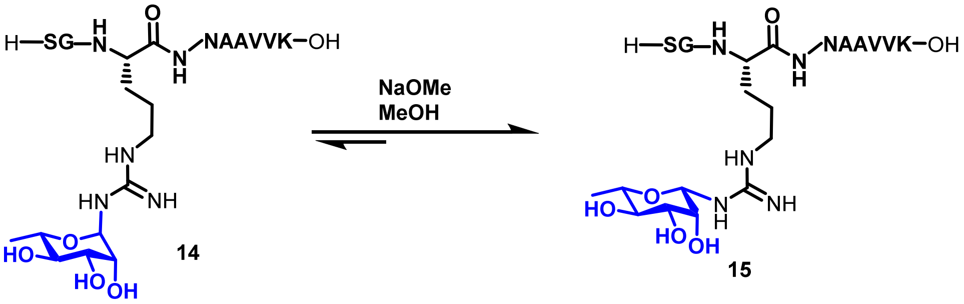


To a solution of α-rhamnosylated peptide **14** (0.8 mg, α:β 9:1, obtained in Section 4.1) in MeOH (1 mL) was added NaOMe (0.5 M in MeOH, 0.1 mL), to give a final pH 8.5-9. The resulting mixture was monitored with analytical RP-HPLC (fluorophenyl column).

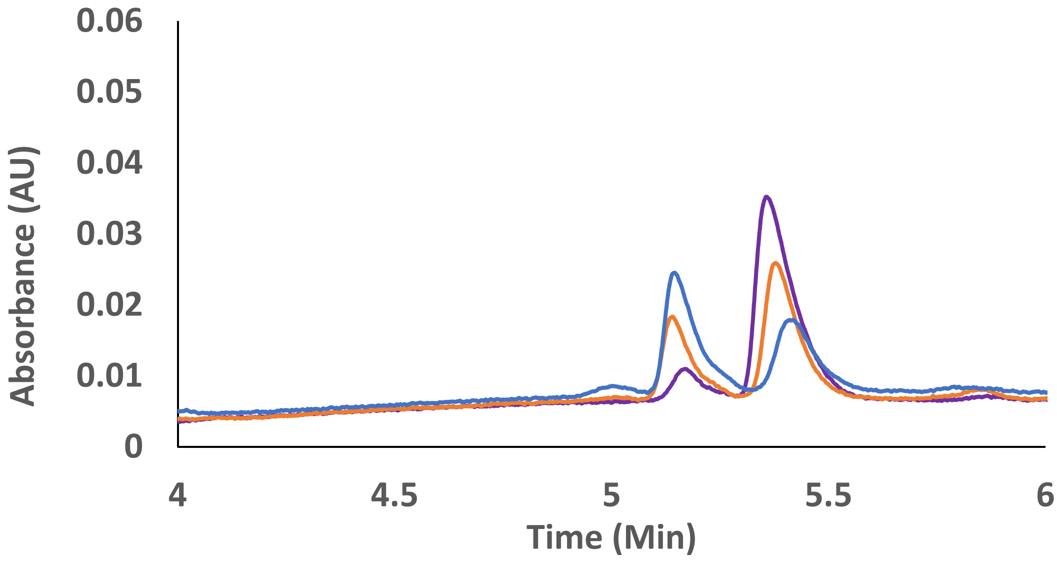


Figure 80: Time-course monitoring of anomerization between glycopeptide **14** ($t_R$ = 5.2 min) and **15** ($t_R$ = 5.4 min). Purple: t = 0; Orange: t = 30 min; Blue: t = 2 hrs. gradient: 0 to 8% B over 8 min with a flow rate of 0.6 mL/min.

## 6. His tagged purification of Pseudomonas aeruginosa EF-P

Protein purification was carried out as previously described[6] with minor alterations. In brief, we obtained *in vivo* rhamnosylated EF-P by expressing *Pseudomonas aeruginosa* N-terminal $His_6$-EF-P in the background of the *P. aeruginosa* PAK strain. Isolation of unmodified *P. aeruginosa* EF-P was achieved by expressing N-terminal $His_6$-EF-P in a XJB BL21(DE3) background. Clarified lysates were loaded onto a gravity column with TALON resin, and after 50 column volumes of wash buffer (10 mM Tris-HCl pH 7.4, 500 mM NaCl, 5mM imidazole) the proteins were eluted with wash buffer supplemented with 200mM imidazole. Elutent fractions were pooled, concentrated and dialyzed against 10 mM Tris-pH 6.8, 10 mM NaCl, and 2 mM BME. Following dialysis, the majority of contaminant proteins were removed on a DEA Sepharose column, using a step-wise gradient from 10 mM NaCl to 500 mM NaCl. EF-P was determined by gel electrophoresis to elute between 100 mM and 300 mM NaCl and these fractions were pooled, concentrated and dialyzed against 10mM Tris-pH 7.4, 100 mM NaCl, 2 mM BME, and 10% glycerol.

## 7. *in vitro* rhamnosylation of EF-P.

*In vitro* rhamnosylation was performed with 2 μM of recombinant $His_6$-EarP and 500 μM of recombinant $His_6$-EF-P, 500 μM dTDP-L-rhamnose (Carbosynth), 1 mM $MgCl_2$, 10 mM Tris-HCl pH 7.4, and 100 mM NaCl. The reaction was carried out at 37˚C for 1 hour and quenched by flash-freezing with liquid nitrogen.

## 8. Protease digestion and peptide preparation

Purified EF-P was digested with Lys-C (1:50 enzyme:substrate ratio) in 50 mM Tris.HCl (pH 6.8) overnight at 37 ºC. Peptides were acidified to a final concentration of 0.2% trifluoroacetic acid and desalted with a C18 microcolumn containing porous oligo R2/R3 reversed-phase media and dried by vacuum centrifugation.

## 9. nanoUHPLC-MS/MS

Peptides were resuspended in 0.1% formic acid and analyzed on a Dionex 3500RS nanoUHPLC coupled to an Orbitrap Fusion mass spectrometer in positive mode. Separation was achieved using an in-house packed 75 µm x 50 cm pulled column (1.9 µm particle size, C18AQ; Dr Maisch, Germany) by isocratic elution of 5% acetonitrile containing 0.1% formic acid over 30 min at 250 nL/min. An MS1 scan was acquired from 300 – 1500 *m/z* (120,000 resolution Orbitrap Fusion; $5e^5$ AGC, 100 ms injection time) followed by targeted MS/MS (349.8646 *m/z*) with either CID or HCD as specified and detection in the ion-trap ($2e^4$ AGC, 70 ms injection time, 10 to 40 NCE with steps of 5 NCE, 0.25 Activation Q, 2.0 m/z quadrupole isolation width) or ETD and detection in the Orbitrap (2e5 AGC, 120 ms injection time, 6e4 resolution, 54 ms reaction time). All data was manually analyzed in Xcalibur and annotated using GPMAW (Lighthouse Data, Denmark).

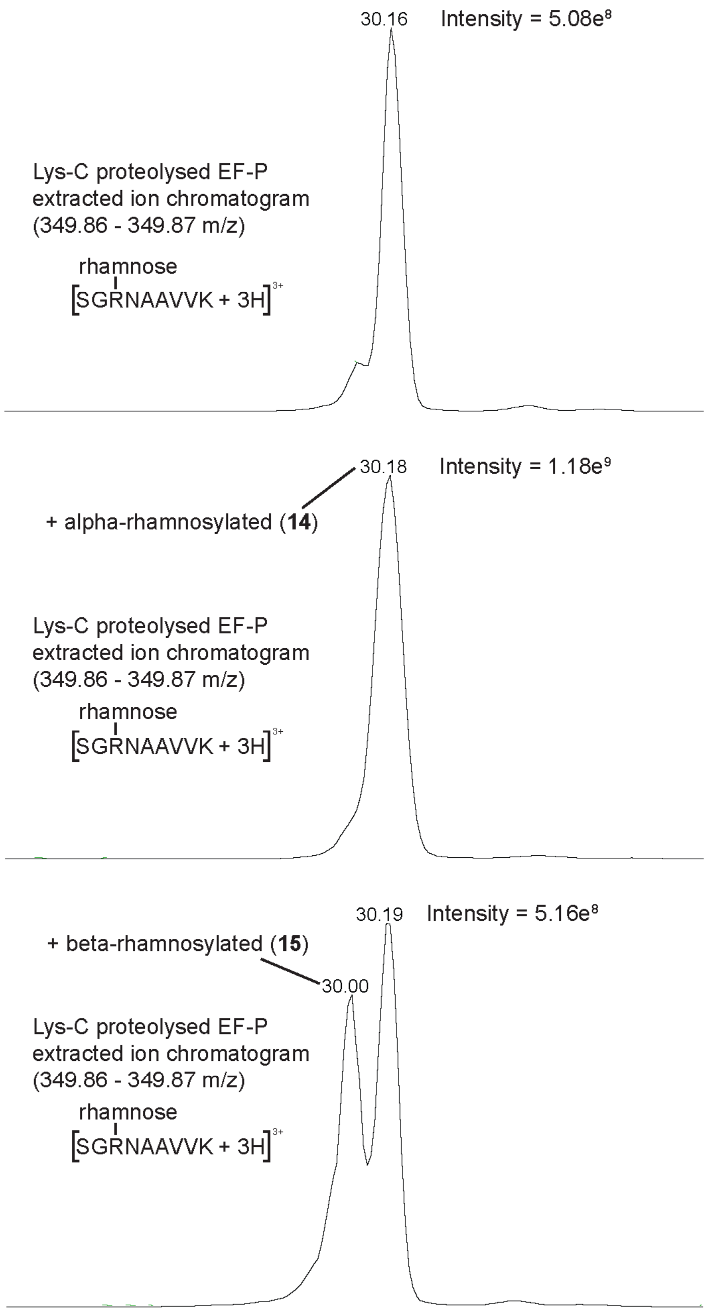


Figure 81. Extracted ion chromatogram of the Lys-C proteolysed *P. aeruginosa* EF-P rhamnosylated glycopeptide (349.86 – 349.87 *m/z*) separated by nanoUHPLC (top). Separation was also performed by spiking either the α-rhamnosylated (**14**) (middle) or β-rhamnosylated synthetic glycopeptide (**15**) (bottom) into Lys-C proteolysed sample of *P. aeruginosa* EF-P. Accumulated intensities of the separated glycopeptides further support α-configuration of the rhamnose moiety.

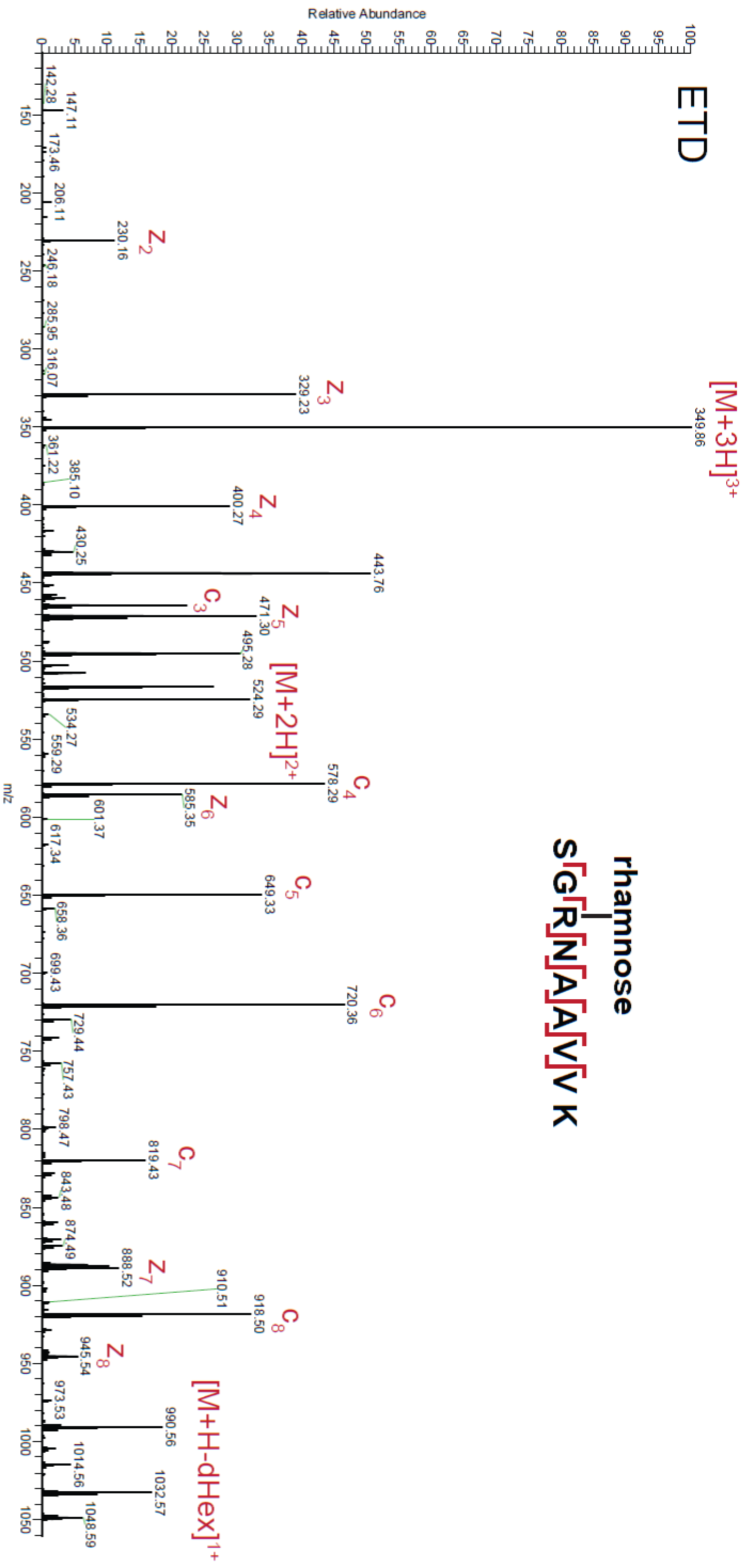


Figure 82: Electron transfer dissociation (ETD) MS/MS of the rhamnosylated glycopeptide from *P. aeruginosa* localizing the site of modification to Arg32 of EF-P. All ions are annotated to within 20 ppm.

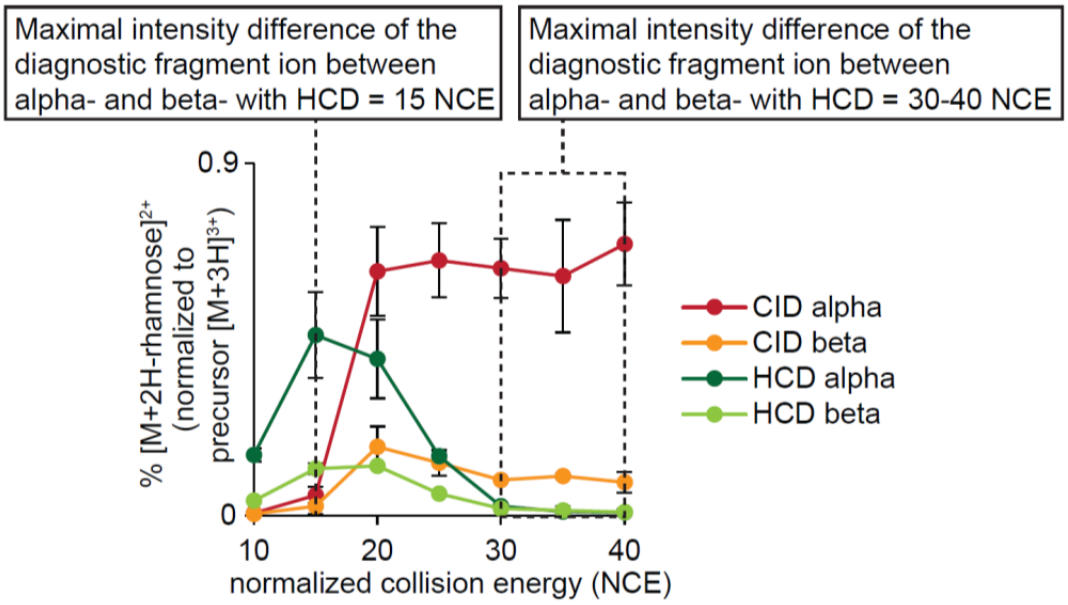


Figure 83. Synthetic α- and β-rhamonsylated glycopeptides **14** and **15** analysed by CID or higher collisional dissociation (HCD) MS/MS with increasing collision energies (10 – 40 NCE with steps of 5 NCE). Plotted is the distribution of the diagnostic fragment ion $[M+2H\text{-rhamnose}]^{2+}$ intensity (normalized to the precursor ion $[M+3H]^{3+}$ intensity). Maximal intensity differences to distinguish between □- and □-anomers are shown with the dotted lines revealing a NCE of 15% gives the greatest separation with HCD while an NCE of 30 – 40% gives the greatest separation with CID.

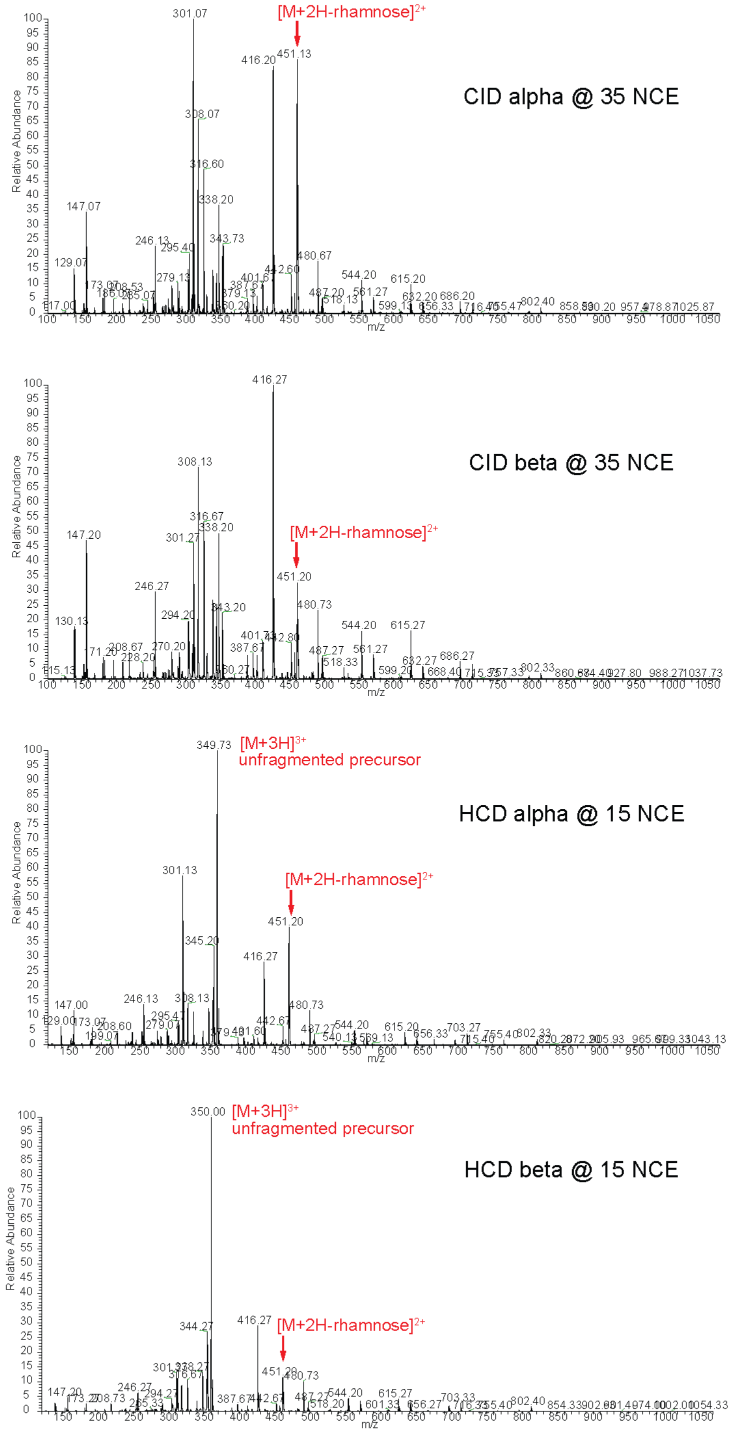


Figure 84. CID and HCD MS/MS of the synthetic α- and β-rhamnosylated glycopeptides **14** and **15** $[M+3H]^{3+}$ with either 35 NCE (CID) or 15 NCE (HCD). Highlighted is the diagnostic fragment ion $[M+2H\text{-rhamnose}]^{2+}$ and the non-fragmented precursor ion.

## 10. References


1. Gottlieb, H. E.; Kotlyar, V.; Nudelman, A., NMR Chemical Shifts of Common Laboratory Solvents as Trace Impurities. *The Journal of Organic Chemistry* **1997,** *62* (21), 7512-7515.
2. Bubb, W. A., NMR spectroscopy in the study of carbohydrates: Characterizing the structural complexity. *Concepts in Magnetic Resonance Part A* **2003,** *19A* (1), 1-19.
3. Yu, B.; van Ingen, H.; Vivekanandan, S.; Rademacher, C.; Norris, S. E.; Freedberg, D. I., More accurate 1JCH coupling measurement in the presence of 3JHH strong coupling in natural abundance. *Journal of Magnetic Resonance* **2012,** *215*, 10-22.
4. Wang, Q.; Fu, J.; Zhang, J., A facile preparation of peracylated α-aldopyranosyl chlorides with thionyl chloride and tin tetrachloride. *Carbohydrate Research* **2008,** *343* (17), 2989-2991.
5. Li, X.; Krafczyk, R.; Macosek, J.; Li, Y.-L.; Zou, Y.; Simon, B.; Pan, X.; Wu, Q.-Y.; Yan, F.; Li, S.; Hennig, J.; Jung, K.; Lassak, J.; Hu, H.-G., Resolving the [small alpha]-glycosidic linkage of arginine-rhamnosylated translation elongation factor P triggers generation of the first ArgRha specific antibody. *Chemical Science* **2016**, *7* (12), 6995-7001.
6. Rajkovic, A.; Erickson, S.; Witzky, A.; Branson, O. E.; Seo, J.; Gafken, P. R.; Frietas, M. A.; Whitelegge, J. P.; Faull, K. F.; Navarre, W.; Darwin, A. J.; Ibba, M., Cyclic Rhamnosylated Elongation Factor P Establishes Antibiotic Resistance in Pseudomonas aeruginosa. *mBio* **2015,** *6* (3), e00823.
